# Korea4K: whole genome sequences of 4,157 Koreans with 107 phenotypes derived from extensive health check-ups

**DOI:** 10.1093/gigascience/giae014

**Published:** 2024-04-16

**Authors:** Sungwon Jeon, Hansol Choi, Yeonsu Jeon, Whan-Hyuk Choi, Hyunjoo Choi, Kyungwhan An, Hyojung Ryu, Jihun Bhak, Hyeonjae Lee, Yoonsung Kwon, Sukyeon Ha, Yeo Jin Kim, Asta Blazyte, Changjae Kim, Yeonkyung Kim, Younghui Kang, Yeong Ju Woo, Chanyoung Lee, Jeongwoo Seo, Changhan Yoon, Dan Bolser, Orsolya Biro, Eun-Seok Shin, Byung Chul Kim, Seon-Young Kim, Ji-Hwan Park, Jongbum Jeon, Dooyoung Jung, Semin Lee, Jong Bhak

**Affiliations:** Korean Genomics Center (KOGIC), Ulsan National Institute of Science and Technology (UNIST), Ulsan 44919, Republic of Korea; Clinomics, Inc., Ulsan 44919, Republic of Korea; Korean Genomics Center (KOGIC), Ulsan National Institute of Science and Technology (UNIST), Ulsan 44919, Republic of Korea; Department of Biomedical Engineering, College of Information-Bio Convergence Engineering, Ulsan National Institute of Science and Technology (UNIST), Ulsan 44919, Republic of Korea; Korean Genomics Center (KOGIC), Ulsan National Institute of Science and Technology (UNIST), Ulsan 44919, Republic of Korea; Clinomics, Inc., Ulsan 44919, Republic of Korea; Korean Genomics Center (KOGIC), Ulsan National Institute of Science and Technology (UNIST), Ulsan 44919, Republic of Korea; Department of Biomedical Engineering, College of Information-Bio Convergence Engineering, Ulsan National Institute of Science and Technology (UNIST), Ulsan 44919, Republic of Korea; Department of Mathematics, Kangwon National University, Chuncheon 24341, Republic of Korea; Korean Genomics Center (KOGIC), Ulsan National Institute of Science and Technology (UNIST), Ulsan 44919, Republic of Korea; Department of Biomedical Engineering, College of Information-Bio Convergence Engineering, Ulsan National Institute of Science and Technology (UNIST), Ulsan 44919, Republic of Korea; Korean Genomics Center (KOGIC), Ulsan National Institute of Science and Technology (UNIST), Ulsan 44919, Republic of Korea; Department of Biomedical Engineering, College of Information-Bio Convergence Engineering, Ulsan National Institute of Science and Technology (UNIST), Ulsan 44919, Republic of Korea; Korean Genomics Center (KOGIC), Ulsan National Institute of Science and Technology (UNIST), Ulsan 44919, Republic of Korea; Clinomics, Inc., Ulsan 44919, Republic of Korea; Korean Genomics Center (KOGIC), Ulsan National Institute of Science and Technology (UNIST), Ulsan 44919, Republic of Korea; Department of Biomedical Engineering, College of Information-Bio Convergence Engineering, Ulsan National Institute of Science and Technology (UNIST), Ulsan 44919, Republic of Korea; Korean Genomics Center (KOGIC), Ulsan National Institute of Science and Technology (UNIST), Ulsan 44919, Republic of Korea; Department of Biomedical Engineering, College of Information-Bio Convergence Engineering, Ulsan National Institute of Science and Technology (UNIST), Ulsan 44919, Republic of Korea; Korean Genomics Center (KOGIC), Ulsan National Institute of Science and Technology (UNIST), Ulsan 44919, Republic of Korea; Department of Biomedical Engineering, College of Information-Bio Convergence Engineering, Ulsan National Institute of Science and Technology (UNIST), Ulsan 44919, Republic of Korea; Korean Genomics Center (KOGIC), Ulsan National Institute of Science and Technology (UNIST), Ulsan 44919, Republic of Korea; Department of Computer Science & Engineering (CSE), College of Information-Bio Convergence Engineering, Ulsan National Institute of Science and Technology (UNIST), Ulsan 44919, Republic of Korea; Clinomics, Inc., Ulsan 44919, Republic of Korea; Korean Genomics Center (KOGIC), Ulsan National Institute of Science and Technology (UNIST), Ulsan 44919, Republic of Korea; Department of Biomedical Engineering, College of Information-Bio Convergence Engineering, Ulsan National Institute of Science and Technology (UNIST), Ulsan 44919, Republic of Korea; Lee Gil Ya Cancer and Diabetes Institute, Gachon University, Incheon 21999, Republic of Korea; Clinomics, Inc., Ulsan 44919, Republic of Korea; Clinomics, Inc., Ulsan 44919, Republic of Korea; Korean Genomics Center (KOGIC), Ulsan National Institute of Science and Technology (UNIST), Ulsan 44919, Republic of Korea; Clinomics, Inc., Ulsan 44919, Republic of Korea; Clinomics, Inc., Ulsan 44919, Republic of Korea; Korean Genomics Center (KOGIC), Ulsan National Institute of Science and Technology (UNIST), Ulsan 44919, Republic of Korea; Department of Biomedical Engineering, College of Information-Bio Convergence Engineering, Ulsan National Institute of Science and Technology (UNIST), Ulsan 44919, Republic of Korea; Korean Genomics Center (KOGIC), Ulsan National Institute of Science and Technology (UNIST), Ulsan 44919, Republic of Korea; Department of Biomedical Engineering, College of Information-Bio Convergence Engineering, Ulsan National Institute of Science and Technology (UNIST), Ulsan 44919, Republic of Korea; Korean Genomics Center (KOGIC), Ulsan National Institute of Science and Technology (UNIST), Ulsan 44919, Republic of Korea; Department of Biomedical Engineering, College of Information-Bio Convergence Engineering, Ulsan National Institute of Science and Technology (UNIST), Ulsan 44919, Republic of Korea; Geromics Ltd., Cambridge CB1 3NF, United Kingdom; Clinomics Europe Ltd., Budapest 1094, Hungary; Department of Cardiology, Ulsan University Hospital, University of Ulsan College of Medicine, Ulsan 44033, Republic of Korea; Clinomics, Inc., Ulsan 44919, Republic of Korea; Korea Bioinformation Center, Korea Research Institute of Bioscience and Biotechnology, Daejeon 34141, Republic of Korea; Korea Bioinformation Center, Korea Research Institute of Bioscience and Biotechnology, Daejeon 34141, Republic of Korea; Korea Bioinformation Center, Korea Research Institute of Bioscience and Biotechnology, Daejeon 34141, Republic of Korea; Department of Biomedical Engineering, College of Information-Bio Convergence Engineering, Ulsan National Institute of Science and Technology (UNIST), Ulsan 44919, Republic of Korea; Korean Genomics Center (KOGIC), Ulsan National Institute of Science and Technology (UNIST), Ulsan 44919, Republic of Korea; Department of Biomedical Engineering, College of Information-Bio Convergence Engineering, Ulsan National Institute of Science and Technology (UNIST), Ulsan 44919, Republic of Korea; Korean Genomics Center (KOGIC), Ulsan National Institute of Science and Technology (UNIST), Ulsan 44919, Republic of Korea; Clinomics, Inc., Ulsan 44919, Republic of Korea; Department of Biomedical Engineering, College of Information-Bio Convergence Engineering, Ulsan National Institute of Science and Technology (UNIST), Ulsan 44919, Republic of Korea; Personal Genomics Institute (PGI), Genome Research Foundation (GRF), Osong 28160, Republic of Korea

**Keywords:** Korean Genome Project, genome, phenome, population genomics, variome

## Abstract

**Background:**

Phenome-wide association studies (PheWASs) have been conducted on Asian populations, including Koreans, but many were based on chip or exome genotyping data. Such studies have limitations regarding whole genome–wide association analysis, making it crucial to have genome-to-phenome association information with the largest possible whole genome and matched phenome data to conduct further population-genome studies and develop health care services based on population genomics.

**Results:**

Here, we present 4,157 whole genome sequences (Korea4K) coupled with 107 health check-up parameters as the largest genomic resource of the Korean Genome Project. It encompasses most of the variants with allele frequency >0.001 in Koreans, indicating that it sufficiently covered most of the common and rare genetic variants with commonly measured phenotypes for Koreans. Korea4K provides 45,537,252 variants, and half of them were not present in Korea1K (1,094 samples). We also identified 1,356 new genotype–phenotype associations that were not found by the Korea1K dataset. Phenomics analyses further revealed 24 significant genetic correlations, 14 pleiotropic associations, and 127 causal relationships based on Mendelian randomization among 37 traits. In addition, the Korea4K imputation reference panel, the largest Korean variants reference to date, showed a superior imputation performance to Korea1K across all allele frequency categories.

**Conclusions:**

Collectively, Korea4K provides not only the largest Korean genome data but also corresponding health check-up parameters and novel genome–phenome associations. The large-scale pathological whole genome–wide omics data will become a powerful set for genome–phenome level association studies to discover causal markers for the prediction and diagnosis of health conditions in future studies.

## Background

South Korea has perhaps one of the most extensive and convenient annual health check-up services. Every year, almost all Koreans aged over 40 years receive a standardized health check-up, yielding a wealth of individual clinical data [1]. In 2020, we published 1,094 whole genomes with clinical information (Korea1K) by providing all the participants with a free standard health check-up showing the value of whole genome data accompanied by clinical information mapping the genome diversity with practical applications [2]. Here, we present the second phase of the Korean Genome Project (KGP) with 4,157 sets of whole genome data, Korea4K. It is accompanied by 107 types of clinical traits that have been donated by 2,685 healthy participants who acquired the health check-up reports from the hospitals of their choice in the past years. We manually annotated thousands of donated health reports that are matched with the whole genome information. Therefore, apart from the increased number of samples, the main difference between Korea1K and Korea4K is that Korea4K’s clinical information is from very heterogeneous but fairly standard Korean health check-up centers, while Korea1K was from one very well-controlled university hospital health check-up center. This was also a testbed to assess how difficult it would be to merge data from the heterogeneous health check-up record system in a nation for a large-scale genome to phenome association analysis.

Previously, there were a few phenome-wide association studies (PheWASs) on Asian populations, but they were limited to chip- or exome-based genotyping data. A Japanese PheWAS identified the genetic links among clinical traits, complex diseases, and cell type–specific patterns [3]. Another PheWAS using 10,000 Korean cohorts’ health check-up data from multiple lab sources showed network relationships between genes and phenotypes [4]. However, none of these studies covered the entirety of genomic variation, and they have limitations on genome-wide data analyses [5, 6].

A scientific contribution of this version of KGP is that we provide extensive genome-to-phenome association information with the largest genomic and clinical data from Korea to date to estimate how many samples and clinical parameters cover the whole genomic and common phenotypic diversity of Koreans. Korea4K contains 4,157 Korean genomes from East Asian ancestry, and 2,685 of them are accompanied by 107 types of clinical information such as height, waist circumference, weight, albumin/globulin ratio, basophil, direct bilirubin, low-density lipoprotein, high-density lipoprotein, mean corpuscular volume, and total cholesterol. The rest does not contain such kind of data because the biobank does not have phenotype information, or we were not able to collect it from the participants. Korea4K extends the efforts to completely map the totality of Korean genomic diversity, which can be a useful scope reference for disease risk prediction, diagnosis, and treatments in the future for personalized medicine.

As the second phase of the KGP, Korea4K not only extends the previously reported Korea1K [2] but also includes new multiphenotypic association analyses, that is, analyses on markers that are associated with multiple phenotypes (pleiotropy), the genetic correlation between traits, and estimated causality relationship among traits through Mendelian randomization (MR) and 3-dimensional (3D) structure models for Korean-specific missense variants. Combining these 2 omics data, we provide the community with the most extensive genotype–phenotype association of healthy Korean participants. We have also applied the genomic variation data to the genotype imputation of low-frequency variants in the Korean population.

## Data Description

The goal of our project was to create a genome dataset for Korea4K, which included newly sequenced genomic data from 2,848 participants as well as 1,309 whole genome sequencing (WGS) datasets from Korea1K and public data archives. Additionally, we established a phenome dataset for Korea4K by gathering or computing 107 clinical parameters and genome data from 2,685 samples. We collected a total of 3,383 clinical datasets, including multiple time points per sample, from regular health check-ups conducted by various hospitals and clinics across Korea between 2016 and 2019. The genome and phenome datasets were produced and curated by the protocol in Materials and Methods.

## Analyses

### The largest Korean whole genome variants data: Korea4K variome

**Figure 1:**
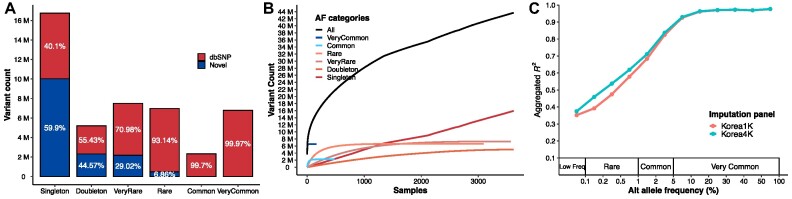
Korean variome profile and imputation evaluation using Korea4K. (A) The number of variants in the Korea4K variome is categorized by AFs among unrelated Korea4K genomes. “dbSNP” indicates the variants were reported in dbSNP database. “Novel” indicates the variants were not reported in dbSNP. Singleton, allele count = 1; doubleton, allele count = 2; very rare, allele count of >2 and allele frequency of ≤0.001; rare, allele frequency of >0.001 and allele frequency of ≤0.01; common, allele frequency of >0.01 and allele frequency of ≤0.05; very common, allele frequency of >0.05. (B) The number of discovered variants as a function of unrelated genomes. (C) Imputation performance evaluation using the Korea4K and Korea1K panels. The x-axis indicates alternative (Alt) allele frequency in the Korea4K variome. The y-axis represents the aggregated *R*^2^ values of variants. We used variants that were overlapped by imputed results across 2 panels.

A total of 64,301,272 single nucleotide variants (SNVs) and 8,776,608 indels were called against the human genome reference (hg38) from the 4,157 Korean whole genomes, including 3,071 healthy controls (Supplementary Tables S1 and S2). It contains 3,063 newly added whole genomes sequenced by Illumina next-generation sequencing (NGS) platforms (HiSeq X10 and Novaseq 6000), in addition to the previous Korea1K dataset, which was mostly generated by Illumina HiSeq X10. Using the variant data, we selected 3,617 samples with no kinship after initial sample filtering (see Materials and Methods). To exclude erroneous variants from sequencing batch effects from the heterogeneous Illumina NGS platforms and library preparation, we applied an allele balance bias measurement and finally acquired 12,713,580 erroneously called variant candidates (Supplementary Fig. S1). After additional variant filtering (see Materials and Methods), we identified 45,537,252 variants, including 42,124,137 SNVs, 36,029 double nucleotide variants (DNVs), 26,135 triple nucleotide variants (TNVs), 3,261,682 indels, and 89,269 other types of small variants from the 3,617 unrelated samples. We named this filtered Korean dataset the Korea4K variome (Fig. 1). A total of 23,689,147 variants were not present in the previous Korea1K variome. This unexpectedly large difference is likely derived from different batch effect filtering and variant calling and filtering procedures, as well as new variants from the larger sample size. Consistent with the Korea1K study [2], most variants were located in intronic or intergenic regions and rarely in splicing sites or coding regions (Supplementary Fig. S2), which is a sign of negative selection pressure in the population. Half of the total variants (21,941,879; 48.2%) were singleton or doubleton in the 3,617 unrelated samples, indicating that the Korean population’s genetic diversity is very low as the population diversity could be covered by fewer than 4,000 unrelated samples (Fig. 1A, Supplementary Table S3). Almost all the common (allele frequency of >0.01 and allele frequency of ≤0.05) and very common (allele frequency of >0.05) variants were found to be already reported in the database of single nucleotide polymorphisms (dbSNP) (99.70% and 99.97%, respectively), while more than half of the singleton and doubleton variants were newly discovered in this study (59.9% and 44.57%, respectively), indicating the new variant pool is well exhausted in the Korean population by the 3,617 samples, resulting in a large portion of individual specific novel variants in the Korean variome (Fig. 1A, Supplementary Table S3). Only 3,092 and 3,569 unrelated individuals were needed to discover all the rare (allele frequency of >0.001 and allele frequency of ≤0.01) and very rare (allele count of >2 and allele frequency of ≤0.001) variants in the Korea4K variome, respectively (Fig. 1B), indicating that the Korea4K variome includes almost all the rare and very rare variants of Korean people of East Asian ancestry. It is notable that in our previous Korea1K data, the accumulated variant number curves did not reach a plateau [2]. Regarding common variants, only 481 and 161 unrelated individuals were necessary for common and very common variants, respectively, to cover the diversity that is close to the Korea1K statistics (440 and 132 samples). Essentially, the Korea4K variome statistics indicate the saturation of population diversity detection among Koreans. However, as expected, in the case of singleton and doubleton variants, the Korea4K variant discovery curve did not reach a plateau. This is due to each individual’s novel random variants, and we will never reach a point of no novel variant discovery even with increased sample numbers.

As a practical application, we constructed a Korea4K imputation reference panel from the 3,614 unrelated whole genomes that showed a consistently better imputation performance than the Korea1K. The Korea4K panel was able to impute 198,805 more genotypes than the Korea1K panel (7,551,095 loci compared to 7,352,290) with the same dataset. Moreover, as expected, the Korea4K panel had better accuracy across all allele frequency categories than the Korea1K panel (Fig. 1C). The difference in aggregated *R*^2^ became larger for variants with allele frequency (AF) in Korea4K <0.05 than for those in Korea1K, indicating higher accuracy in rare variants (Fig. 1C). In particular, the Korea4K imputation panel improved the imputation accuracy by 6% for the rare variants group compared to Korea1K on average.

**Figure 2:**
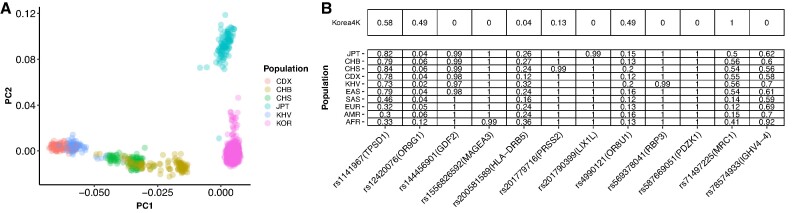
Comparison of Korea4K and 1KGP. (A) The results from PCA of Korea4K and the 1KGP set of East Asian samples. (B) Allele frequency information of Korea4K and the populations in the 1KGP for the 12 Korean-specific missense variants located in protein functional domains. AFR: African; AMR: American; CDX: Dai Chinese; CHB: Han Chinese; CHS: Southern Han Chinese; EAS: East Asians; EUR: European; JPT: Japanese; KHV: Kinh Vietnamese; KOR: Korea4K; SAS: South Asians.

As in Korea1K, the Korean population is genetically distinct from the Chinese and Japanese populations, confirmed by principal component analysis (PCA) with few outliers (Fig. 2A). We also found 62 missense variants out of 282,607 in Korea4K that had AFs significantly different from 10 populations in the 1000 Genomes Project (1KGP) from the European Bioinformatics Institute (EBI) (χ^2^ test *P* < 5 × 10^−5^ against each of the 10 populations; see Materials and Methods; Supplementary Table S4). The genes containing such Korean-specific missense variants included *LILRB3, HLA-DRB5, IGLV5-48*, and *IGHV4-4*, which are known to be associated with adaptive immunity, and *OR9G1* and *OR8U1* for olfactory receptors. Additionally, we found that 12 Korean-specific missense variants were in protein functional domains (Fig. 2B). Four of them were predicted to facilitate increased structural stability calculated in the protein 3D models built by AlphaFlod2 [7], while the other 8 variants were predicted to cause decreased stability (Supplementary Table S5).

### Whole genome–wide association study (WGWAS)

**Figure 3:**
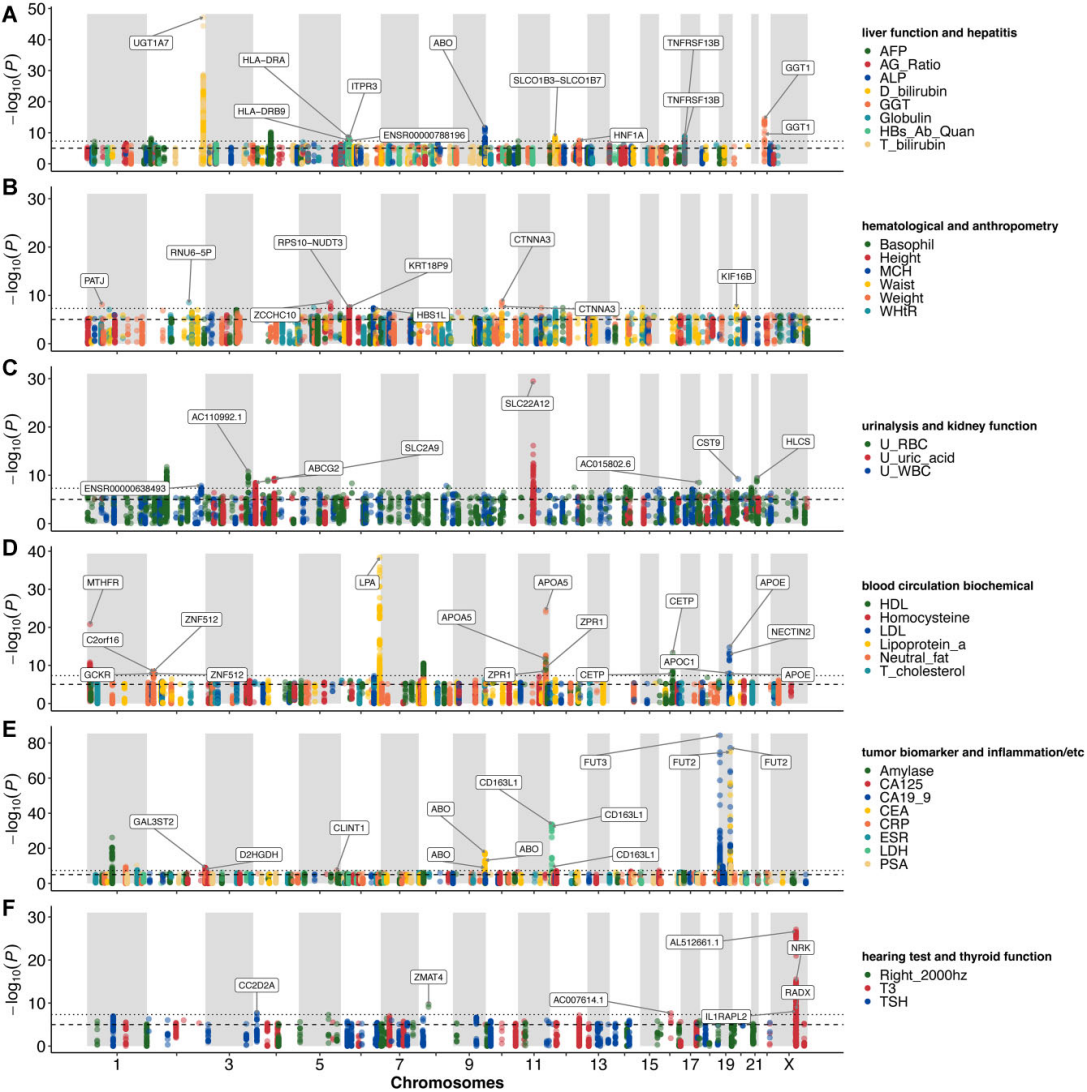
Whole genome–wide association studies in Korea4K. (A–F) Whole genome–wide association studies from 34 traits. Loci are presented only when index variants of the loci had a significant *P* value (*P* < 5 × 10^−8^) from the WGWAS. The dashed line indicates the suggestive threshold (*P* < 10^−5^). The dotted line indicates the significant threshold (*P* < 5 × 10^−8^).

Whole genome–wide association studies (WGWASs) revealed that 2,324 variants from 157 unique loci had significant associations with 34 clinical traits from 37 WGWAS target traits (*P* < 5 × 10^−8^; Fig. 3A–F, Supplementary Table S6). Among the significantly associated variants, 2,314 variants from 30 clinical traits still showed significance after false discovery rate (FDR) correction using the Benjamini–Hochberg approach (FDR < 0.05). We used 90 clinical traits from the 107 phenotypes after filtering 27 traits with a high missing rate and biased distribution for WGWASs (see Materials and Methods). Of the 90 traits, 54 were not confident in quantile–quantile (QQ) plots and were excluded from further MR and pleiotropy analyses (see Materials and Methods). Among the 2,324 WGWAS significant variants, only 85 variants (31 loci) were reported in the genome-wide association studies (GWAS) catalog database [8]. The trait with the largest number of significantly associated loci was carbohydrate antigen 19-9 (CA19-9), a cancer antigen, with 16 loci. Uric acid had the second highest number of significant loci with 14 loci.

Korea4K showed much stronger statistical power than the previous Korea1K study, identifying 1,356 new WGWAS variants (107 loci) from 28 common traits between Korea4K and Korea1K. Also, Korea4K had much lower (i.e., more significant) *P* values than Korea1K for all the commonly found association variants between the 2 datasets (Supplementary Fig. S3). Among the 107 loci containing the 1,356 new WGWAS variants, 798 Korea4K significant WGWAS variants from 73 loci had not been significant in Korea1K (Supplementary Table S6). Furthermore, 12 traits (albumin/globulin ratio, basophil, C-reactive protein, direct bilirubin, height, low-density lipoprotein, mean corpuscular volume, right hearing at 2000 Hz, thyroid stimulating hormone, total cholesterol, waist, weight) had 425 WGWAS variants that were significant uniquely in Korea4K, meaning no significant WGWAS variants from the 12 traits in Korea1K (Supplementary Table S6). For example, a missense variant, rs6431625 (*P* = 1.41 × 10^−23^, FDR = 5.23 × 10^−18^), in *UGT1A3* was found to be associated with direct bilirubin in Korea4K. It was previously reported to be associated with circulating bilirubin levels [9]. Another Korea4K-specific missense variant is rs7412 (*P* = 2.86 × 10^−14^, FDR = 1.11 × 10^−7^) in *APOE*, which is associated with low-density lipoprotein (LDL) levels. Its association with cholesterol levels has been previously well established [10]. Finding novel WGWAS variants in Korea4K was due to the increased sample size and subsequently increased variant number compared to Korea1K.

### Genetic correlation and phenotypic correlation

**Figure 4:**
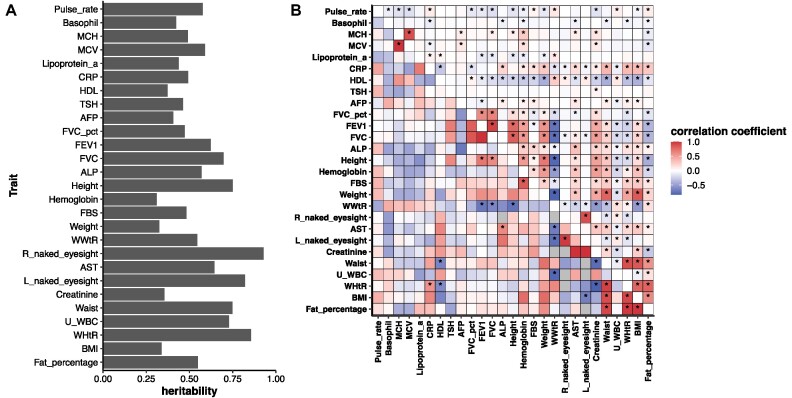
Genetic correlation and phenotypic correlation in Korea4K. (A) Genetic heritability of 27 traits that showed at least a marginal significance. (B) Genetic correlation and phenotypic correlation between the 27 traits. The upper triangle indicates phenotypic correlation coefficient (Pearson’s) and lower triangle indicates genetic correlation coefficient (rG).

We found 27 traits with significant heritability among 89 quantitative traits (Fig. 4A; the lower boundary of genetic heritability >0 with 95% confidence interval [CI]; Supplementary Table S7). A total of 24 pairs of traits showed a significant genetic correlation (GC) (FDR_GC_ < 0.05), measured as rG value, among 351 trait pairs between the 27 traits that showed significant heritability (Fig. 4, Supplementary Table S8). We found consistent results of weight–waist and body mass index (BMI)–waist pairs, showing a significant genetic correlation in the UK Biobank data with the same trend as our result (rG = 0.9, *P* = 10^−308^ in UK Biobank; rG = 0.9, *P* = 10^−308^ in UK Biobank, respectively) [11]. We identified 2,274 trait–trait relationships that had a significant phenotypic correlation (PC) (FDR_PC_ < 0.05, its 95% CI does not include 0) from trait–trait associations between 3,916 pairs of 89 quantitative traits (Fig. 4B, Supplementary Table S9). Most genetic and phenotypic correlations showed the same direction of correlation. The only 2 exceptions were waist/weight ratio (WWtR)–urine white blood cell (U_WBC) and waist–creatinine, which showed opposite directions. This trend of waist–creatinine has also been reported in a correlation database using UK Biobank data [12]. Such discrepancies between the correlation estimates are possibly derived from the shared environmental factors between a pair of traits, such as dietary habits, that overwhelm the genotypic effects [13, 14]. This proves that the phenotypic correlation is not a mere proxy for the genetic correlation, and consideration of the environmental effect is indispensable for the accurate interpretation of human phenomics [15].

### Pleiotropy and MR

**Figure 5:**
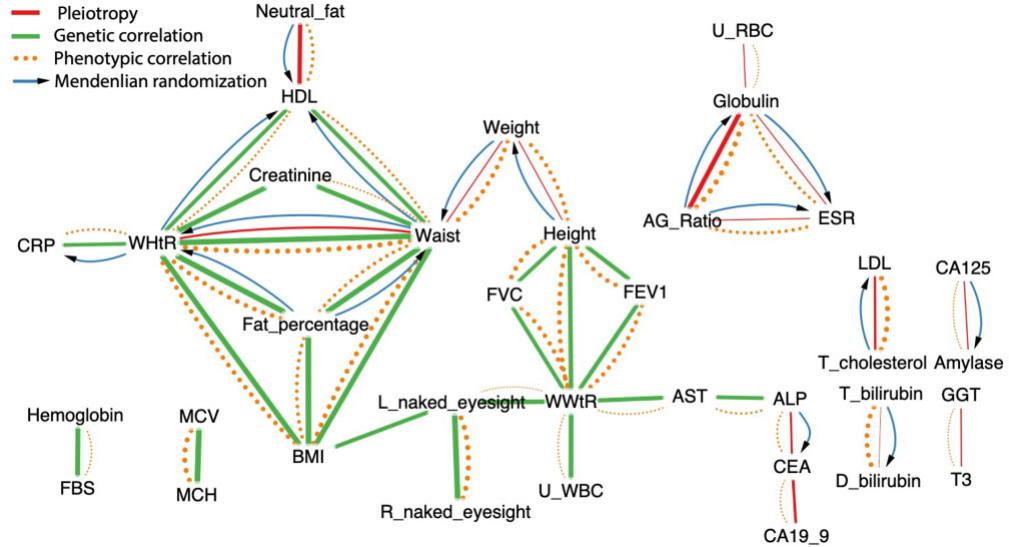
Graph visualization of genetic correlation, phenotypic correlation, pleiotropy, and Mendelian randomization. Green line indicates significant GC, and the edge thickness indicates the absolute value of the correlation coefficient. Red line indicates trait pairs that have pleiotropic variants. Dotted orange lines indicate PC, and the edge thickness indicates the absolute value of Pearson’s correlation coefficient. Blue arrow line indicates a causal relationship from MR. MR and PC were visualized only when at least 1 of the GC or pleiotropy relationships was observed between the traits.

Of the 37 WGWAS target traits, we detected 1,131 variants from 21 traits having suggestive associations (*P_GWAS_* < 10^−5^) with at least 2 traits, indicating pleiotropic variants (Fig. 5, red edges; Supplementary Table S10). We devised the Variant-Sharing Index (VSI) to measure the degree of intersection between 2 phenotypes (Table 1; see Materials and Methods). A VSI of zero signifies that 2 traits share no suggestively associated variants (SSVs), while 100 indicates they share all of them. The trait pairs with SSVs and the corresponding VSIs are listed in Table 1. Notably, we had only 1 variant, rs77913154, that was shared among 3 traits: Globulin, AG_Ratio, and ESR (Supplementary Table S10). Interestingly, we found 15 variants residing on the *SOD2P1–AC095032.2–AC095032.1* locus-forming pleiotropy between the serum amylase level and the level of CA125, a known ovarian cancer marker (Table 1, VSI = 2.3). Fourteen of the 15 variants conform to the alteration of *AMY2B* expression level, as per cis-expression quantitative trait loci (cis-eQTL) results from the GTEx Portal (ver. 8), 4 of which were associated with expression in the pancreatic tissue (see Materials and Methods). There have already been reports of hyperamylasemia in patients with ovarian cancer [16–18]. In addition to the investigation on the general pleiotropic relationship, we employed MR to detect vertical pleiotropy that can assert the direction of the phenotypic relationships [19]. This provides indirect evidence implying causality between the traits to discern spurious phenotypic associations, such as confounding and collider bias [20, 21]. We found that a total of 127 trait pairs among 1,332 pairs of the 37 WGWAS traits were estimated to have significant causal relationships (FDR < 0.05, Fig. 5, Supplementary Table S11). These findings were supported by at least 2 of 3 different MR analysis methods (IVW: 166 pairs; MRPRESSO: 139; MR-Egger: 23). Among these, 59 trait pairs showed unidirectional relationships while 68 exhibited bidirectional causal relationships (Supplementary Table S11).

**Table 1:** Pleiotropic associations and VSI

Trait 1	Trait 2	Suggestive variants in trait 1	Suggestive variants in trait 2	Shared variants	Total variants	VSI
D_bilirubin	T_bilirubin	638	632	569	701	81.2
Globulin	AG_Ratio	294	230	147	377	39
HDL	Neutral_fat	348	398	191	555	34.4
CEA	CA19_9	221	264	74	411	18
T_cholesterol	LDL	74	238	38	274	13.9
WHtR	Waist	177	100	31	246	12.6
ALP	CEA	153	221	35	339	10.3
T3	GGT	542	125	23	644	3.6
CA125	Amylase	202	466	15	653	2.3
Weight	Waist	123	100	5	218	2.3
Height	Weight	173	123	2	294	0.7
ESR	AG_Ratio	163	230	1	392	0.3
Globulin	ESR	294	163	1	456	0.2
U_RBC	Globulin	627	294	1	920	0.1

### Summary results of the 4 phenomics analyses

We summarized the results of 4 phenomics analyses (GC, PC, MR, and pleiotropy) through visualizing them in network plots (Fig. 5). In general, the identified trait–trait pairs of GC, MR, and pleiotropy analyses did not often overlap. Genetic correlation and pleiotropy were found to be exclusive of each other, even though both measures shared genetic components of 2 different traits. GC was primarily observed among body measures such as waist circumference, weight, height, and left naked eyesight. On the other hand, pleiotropy was more prevalent in the relationship between metabolites in blood such as LDL, bilirubin, or carcinoembryonic antigen (CEA). The only overlap between these two was waist-to-height ratio (WHtR)–waist (circumference), where one was derived from the other.

MR suggests a causal relationship between phenotypic correlations through mediation effect by a genotype. In our casual diagram (Fig. 5, blue arrows), alkaline phosphatase (ALP) and CEA showed potential causality, along with the shared genetic variants between them (pleiotropy near *ABO* gene). Numerous previous studies have consistently reported these markers together for diagnosing cancer and monitoring metastasis [22–24]. Similarly, CA125 and amylase also displayed causality via shared genetic variants (pleiotropy near *AMY2B* gene). We propose that CA125 and amylase might serve as complementary biomarkers for ovarian cancer, much like ALP and CEA. The biological relationships between these clinical blood measures remain unclear.

Our phenomics results also depicted distinguishable patterns of association between secondary body measures, such as WHtR, WWtR, and BMI, with other phenotypes. WHtR exhibited a causal relationship with C-reactive protein (CRP), body fat percentage, and high-density lipoprotein (HDL). The result is concordant with previous reports that body fat percentage and CRP are correlated [25, 26]. Conversely, WWtR had casual associations with measures of lung capacity (forced expiratory volume in the first second (FEV_1_) and forced vital capacity (FVC)), liver function (aspartate aminotransferase (AST)), and inflammation (U_WBC). However, WWtR has yet to prove its utility in clinical studies. BMI serves as an intermediate phenotype, sharing most of its associations with WHtR and, to a lesser extent, with WWtR via left naked eyesight. These findings suggest that the measurements reflect distinct biological mechanisms, warranting further studies. For instance, WHtR is a well-known indicator of central adiposity, which provides a better estimate of obesity and related morbidities than BMI [27].

## Discussion

Batch effect exacerbated by sequencing platform and library preparation bias is a critical problem in very large population genome association studies, especially with clinical data from heterogeneous health check-up centers. In the future, more and more diverse whole genome data with extensive clinical data will be publicly available, and it is inevitable that they will be merged for more precise whole genome-to-phenome association research. Korea4K is not an exception in that regard, and in one homogeneous population WGWAS, it was necessary to consider and factor in a great deal of sequencing and clinical data batch effects and errors. We attempted to minimize the errors by using allele balance with optimal filtering criteria and time-consuming manual checks on health reports that were donated by the participants (see Materials and Methods). The largest challenge of the Korea4K project was cleaning up heterogeneous clinical data from different health check-up centers. Another major issue was that the health check-up data heterogeneity caused reduced numbers of participants’ common traits with which to compare. Some of the health data were from past years’ health check-ups from heterogeneous hospitals throughout South Korea. This heterogeneity in location and time was not an intentional experimental design but was in order to reduce the cost of performing expensive 1-center health check-ups for the Korea4K participants. Therefore, WGWAS along with standardized and unified national and public health check-up data will greatly benefit future whole genome–wide association studies.

Although 4,157 seems like a large number, we found the sample size in this study was still not large enough to detect weak association signals. The Korea4K variome with matched phenotype information has allowed us to estimate genomic correlation across various phenotypes using GREML [28]. GREML has been reported to have higher accuracy compared to methods, such as linkage disequilibrium score regression (LDSC), using only summary statistics from GWAS [29]. For example, the minimum heritability score was 0.34 (degree of obesity) among the traits detected as statistically significant. The statistical power of our maximum 2,685 subjects and FDR < 0.05 is estimated to be 0.72 for detecting traits with heritability of 0.3 or higher (calculated from GCTA-GREML Power Calculator) [30]. This will increase to 0.97 with 4,000 subjects. In other words, phenomics analyses were limited and not powerful enough to confidently discover novel phenotypic associations with the current dataset.

Nevertheless, our findings bear important practical implications. We described the utility of secondary body measures, such as WHtR and WWtR, compared to BMI. We also elaborated on the diagnostic and prognostic value of other serum proteins, namely ALP and amylase, in conjunction with the existing cancer biomarkers. However, we plan to collect more samples for sequencing and health record data with a wider variety of health-related categories to conduct a more powerful study in the future. This will allow us to not only validate our findings but also find correlations of medical importance that were missed in the present study. While chip-based GWAS is a common approach, our study highlights the unique advantage of WGWAS in genotype–phenotype association studies. An illustrative advantage of WGWAS is its whole genome–wide, unbiased coverage of genetic variants, which allowed us to assign specific variants accounting for pleiotropy. This was not achievable with conventional methods. For example, we could identify the variants in the well-known pleiotropic relationships such as ALP-CEA by the*ABO* locus (35 variants), Neutral_Fat-HDL by the *LPL* locus (181 variants), and total cholesterol–LDL by the *TOMM40* and *APOE* loci (4 and 2 variants, respectively) (Supplementary Table S7). These loci and their corresponding trait pairs were previously reported from chip-based GWAS summary results [31, 32]. Similarly, we anticipate the fine-mapping analyses will also benefit from WGWAS, pinpointing novel genetic variants of phenotypic importance, as demonstrated in our prior work [33]. Taken together, whole genome sequencing with its genomic completeness should be a well-considered choice for future genomic association studies.

One of the main objectives of the Korea4K project was to build a genomic and phenomic reference dataset to discover unknown whole genome-to-phenome associations that can be detected from samples of healthy people. This, however, is contradictory and it limited us in discovering clear pathogenic associations because most of the participants examined in WGWAS were healthy without any severe aberrant phenotypes or diseases that could bring us clues for interesting omics analyses. Moreover, utilizing recently introduced human genome references like the T2T reference [34] and Human Pangenome reference [35], which offer broader genomic coverage or have population-specific sequences compared to the existing GRCh38 reference, could help identify additional associations that might be overlooked. Nevertheless, these new references lack functional annotations and need to be connected to previous databases such as dbSNP and the GWAS catalog.

As for the future directions, several key limitations have not been met in our current study. The first is we failed to acquire long DNA sequencing reads from the healthy participants for building a structural variation reference set for the Korean population. The second is the lack of epigenomic data from the 4,157 samples. This was mostly due to high costs for generation and computing long-read based assemblies and sequencing methylated DNA sites. The third one, which is perhaps the most relevant for the purpose of performing association studies for health care, is that we failed to acquire more rare and severe disease data from patients, accompanied by precise clinical and multiomics data. We have excluded a small number of rare disease cases, as those required a large amount of sequencing data from genome, transcriptome, and methylome to perform precise functional analyses. Large-scale pathological whole genome–wide omics data will become a powerful set for genome–phenome level association studies to detect causal markers for the prediction and diagnosis of health conditions in future studies.

## Potential Implications

The Korea4K dataset can be a valuable variome reference, as it contains matched phenome data for personalized medicine, large-scale population genome studies, and the understanding of anthropologic history in Korea. This large-scale Korean genome–phenome dataset can help identify the genetic basis for diseases and phenotypes, enabling personalized treatment plans for individuals. Analyzing the genome–phenome association dataset can also be used to develop new drugs that target specific genetic variations in the Korean population. The Korea4K dataset can also be valuable for other populations, particularly East Asians, as it can be used to identify population-specific genome–phenome patterns by comparing the population’s genome–phenome data to the Korea4K dataset. Furthermore, the Korea4K reference panel can be utilized for genotype imputation of DNA chip genotyping data for the Korean population and other East Asians.

## Materials and Methods

### Sample collection and whole genome sequencing

We collected 2,848 blood samples or already processed DNA samples from Korean individuals. A total of 1,094 WGS datasets originating from our previous study (Korea1K) and 215 WGS data from publicly available Clinical & Omics Data Archive (CODA) were added to the aforementioned dataset [2]. The genomic DNA was extracted using the DNeasy Blood & Tissue kit (Qiagen) from whole blood samples. We constructed the whole genome sequencing library from the DNA by using the TruSeq Nano DNA Sample Prep kit (Illumina) kit. Whole genome sequences of the 2,848 samples were generated by the Illumina Nova-seq 6000 platform. All the sequencing data that we used in this study had 151 bp as a read length. Average sequencing amount per sample was 27.75× (Supplementary Fig. S4).

### Joint genotype calling

Adapter contamination was trimmed using Cutadapt (RRID:SCR_011841, ver. 1.9.1) [36] with a forward adapter (′GATCGGAAGAGCACACGTCTGAACTCCAGTCAC′) and reverse adapter (′GATCGGAAGAGCGTCGTGTAGGGAAAGAGTGT′) and with a minimum read length of 50 bp after trimming (Supplementary Fig. S4). The quality of trimmed reads was screened by the FASTQC program (RRID:SCR_014583, ver. 0.11.5) We mapped the whole genome sequencing reads from 4,157 samples to the human reference genome (hg38) using BWA-mem (RRID:SCR_010910, ver. 0.7.17) with the “-M” option and alt-aware mode [37]. The mapped reads were sorted by genomic coordination using Picard (RRID:SCR_006525, ver. 2.20.3). We marked the PCR duplicates and recalibrated the base quality of the mapped reads using the MarkDuplicates and BaseRecalibrator module in Picard (RRID:SCR_006525, ver. 2.20.3), respectively. A total of 3,156 samples had a mapping depth of ≥20× (Supplementary Fig. S4). Individual genotypes were called in GVCF format by HaplotypeCaller in GATK (RRID:SCR_001876, ver. 4.1.3) with “–genotyping-mode DISCOVERY -stand-call-conf 30 -ERC GVCF” options [38]. We merged the individual genotypes to a single GVCF for each chromosome using CombineGVCFs in GATK (RRID:SCR_001876, ver. 4.1.3) [38]. We jointly genotyped the merged GVCF with the genotypeGVCF module in GATK (RRID:SCR_001876, ver. 4.1.3) [38]. Variant quality of the joint genotypes was recalibrated using the VQSR module in GATK (RRID:SCR_001876, ver. 4.1.3) [38].

### Sample and variant filtering

After joint genotyping, we filtered out a total of 540 participants with the criteria listed below using SelectVariants in GATK (RRID:SCR_001876, ver. 4.1.3) with the “–remove-unused-alternates” option to remove unused variants [38]. To explore kinship relations among the samples, we assessed Identical by Descent (IBD) using the Plink program (RRID:SCR_001757, ver. 1.90b3n) [39]. Samples with a PI_HAT value exceeding 0.05 were considered to be in a kinship relation.

1. Showing high missing genotype rate (>10%): 9 samples
2. Having a too high or low heterozygous variants ratio compared to homozygous variants per sample (3 SD): 4 samples
3. Having relatedness to other samples: 428 samples
4. Having non-Korean genetic background from PCA analysis with the 1KGP set: 7 samples
5. Reported to have a rare disease: 40 samples
6. 52 samples that became not applicable for this study

Finally, the Korea4K variome data included 3,617 participants’ genomes. To detect variants that were probably called because of a sequencing batch effect, we measured average allele balance of the genotyped alleles (the read count of the allele divided by the total read count on a locus). Then, we excluded 12,713,580 variants that had average allele balance of the loci out of the range of ± 1 × SD from a genome-wide average of allele balance to remove the sequencing batch effect (Supplementary Fig. S1). We also excluded the variants that had a genotyping rate of <0.9 for downstream variant analysis. The variants in the final variome set were annotated using Variant Effect Predictor (VEP) with Ensemble database (RRID:SCR_007931, ver. 101) [40].

### PCA with the EBI’s 1KGP genome data

The interpopulation genomic structure was evaluated by projecting the first 2 principal components determined via PCA of SNVs from both Korea4K and East Asian populations from 1KGP. We merged variants from the Korea4K and 1KGP sets and then filtered out variants with the following criteria: (i) biallelic SNVs with a minor allele frequency (MAF) <1%, (ii) biallelic SNVs with a Hardy-Weinberg equilibrium (HWE) *P* < 10^−6^, and (iii) biallelic SNVs with a missing genotype rate of >0.01. Extracted variants were linkage disequilibrium (LD) pruned using the “–indep 200 4 0.1” option in PLINK (RRID:SCR_001757, ver. 1.90b3n) [39], yielding 330,350 sites. PCA was carried out using PLINK (RRID:SCR_001757, ver. 1.90b3n) [39].

### Korean-specific missense variants

We collected allele frequency data from 10 populations (African [AFR], American [AMR], European [EUR], South Asian [SAS], East Asian [EAS], Japanese in Tokyo [JPT], Kinh Vietnamese [KHV], Han Chinese in Beijing [CHB], Han Chinese Southern [CHS], and Chinese Dai in Xishuangbanna [CDX]) from the EBI’s 1KGP database [41]. For each Korea4K variant, we compared its allele frequency to the allele frequency of all 10 populations using the χ^2^ test. We selected variants that were specific to the Korean one when the *P* value of the χ^2^ test to the 10 populations was less than 5 × 10^−5^.

### Protein structure modeling and thermodynamic stability measurement

We constructed the mutant-type (MT) protein sequences of the Korean-specific missense variants by substituting the reference protein sequences found in the Ensembl database (RRID:SCR_002344, ver. 101) [42]. We modeled the structures of the wild-type (WT) and mutant-type protein models using AlphaFold2 (ver. 2.0) with the “–max_template_data 2022-05-09 –db_preset reduced_dbs” option with default databases downloaded by AlphaFold2 [7]. We used the InterPro (RRID:SCR_006695) database [43] to determine whether a missense variant was located in the domain region within the protein sequence. We extracted the domain region from the WT and MT protein 3D models and excluded domains that had fewer than 50 amino acids. Afterward, we calculated ΔG_WT_ and ΔG_MT_ using the “Stability” command of foldX (RRID:SCR_008522) [44] to measure the protein thermodynamic stability. Finally, we measured the change in protein thermodynamic stability between the 2 models by calculating the difference between the WT and MT domain models (ΔΔG = ΔG_MT_ − ΔG_WT_).

### Imputation

We constructed an imputation reference panel of Korea4K and Korea1K sets, which includes 3,614 and 873 Korean individuals, respectively. A total of 26,210,741 and 15,649,303 autosomal biallelic variants with a missing genotype call rate of <0.1 and minor allele count >1 (not a singleton) were extracted for the Korea4K and Korea1K panels, respectively. The extracted variomes were phased into haplotype using SHAPEIT2 (ver. v2.r904) [45]. We used the same test dataset as in the previous study [2]. The phased test data were imputed using the imputation reference panel by Minimac3 (RRID:SCR_009292, ver. 2.0.1) [46]. We estimated imputation accuracies using squared Pearson’s correlation coefficients (*R*^2^) between the true genotypes and imputed genotype dosages.

### Clinical information

We collected or calculated 107 clinical parameters (93 quantitative and 14 qualitative traits; Supplementary Table S12) along with genome data from 2,685 samples among the Korea4K samples. A total of 3,383 clinical datasets (including multiple time points per sample) from regular health check-ups carried out by various hospitals and clinics throughout Korea were collected from 2,685 participants between 2016 and 2019. When a single participant had multiple clinical datasets, the most recent one was chosen for the following analysis. Out of the final unrelated 3,617 samples, 2,374 samples had clinical data available and were included in the phenomics analyses.

In the context of collecting data from over 200 diverse health care institutions, standardizing clinical information on 107 traits became imperative. We resolved discrepancies in unit measurements, such as micrograms and nanograms, for specific traits. Furthermore, certain clinical metrics, such as the estimated glomerular filtration rate (eGFR), were found to exhibit variability contingent upon variables such as ethnicity, sex, and age. To maintain consistency and ensure methodological uniformity, we enforced the adoption of a singular clinical formula for the computation of eGFR across all data samples. Such calculations were applied to 26 traits, which are shown in Supplementary Table S13. Clinical traits that exhibited values characterized by inequalities likely due to the limit of detection (e.g., <5.0 and >99) were omitted from the analytical procedures, as such values have the potential to introduce disturbances to subsequent data analyses. Likewise, values that exhibited divergent formatting conventions across distinct health care institutions (e.g., 20 and a few or 999 and many) were harmonized to conform with prevailing standard criteria observed in most samples under investigation. Also, 4 quantitative clinical traits and 12 qualitative traits were excluded from the further analysis, since the traits were missing from more than 90% of participants due to health check-up report heterogeneity, or the traits that were qualitative and biased to 1 category (more than 1:4). Standard weight was also removed from the analysis, because the trait was not an inherently correct representation of the sample’s clinical data but rather a recommended value. Three traits (hepatitis B virus antibody, antigen, and hepatitis C antibody) contained both quantitative and qualitative values. Therefore, both of the values were utilized for analysis (i.e., Hbs_Ab_Quan and Hbs_Ab_Binary). Phenotypic correlations were calculated by Pearson’s method. Benjamini–Hochberg method was used to adjust for multiple comparisons when documenting confident phenotypic correlations with FDR.

### WGWAS

SNVs and indels with a MAF <1%, HWE *P* < 10^−6^, and a missing genotype rate of >0.01 were excluded from the analysis using PLINK (ver. 1.90b3n) [39]. A total of 90 WGWASs (88 quantitative and 2 qualitative traits) were performed with a total of 3,617 individuals and 7,782,381 variants. Each WGWAS had a different number of individuals that included those who had the target clinical traits. The WGWAS was performed using linear and logistic regression under an additive genetic model with PLINK (ver. 2.00 alpha) [47] for quantitative and qualitative traits, respectively. Sex, age, age^2^ (age squared), BMI, and the top 10 principal components of SNV genotypes were included in the model as covariates. Age and BMI were chosen especially due to their known shared associations with multiple traits as previously documented by Shungin and colleagues [48], which could lead to confounding biases in the downstream interpretation of phenotypic relationships. BMI was excluded from covariates in the WGWAS for BMI itself and degree of obesity. Calculating the genomic inflation factor (λ_Median_), we found that all of the traits in the test reside below 1.1, indicating there are minimal false positives caused by gross population structure or systematic biases (Supplementary Figs. S4–S19) [49]. We rejected 53 traits from further analysis based on QQ-plot analysis (Supplementary Figs. S5–S20). We used 5 × 10^−8^ for a whole genome–wide significance threshold. The 7,782,381 variants were clumped into 466,938 loci based on LD information using PLINK (ver. 1.90b3n) with “–clump-p1 1, –clump-p2 1, –clump-r2 0.1, –clump-kb 250, and –clump-index-first” options [39]. Statistical powers of the 90 WGWASs were calculated by the R package “genpwr” under the assumption of an effect size of 0.5 and a minor allele frequency of 0.01 (Supplementary Table S14).

### Measuring heritability and genetic correlation

We calculated genetic relatedness among individuals from single-nucleotide polymorphisms (SNPs) by a genetic relationship matrix in genome-wide complex trait analysis (GCTA) (ver. 1.93.2) with “–autosome –maf 0.01 –make-grm” options [28]. We estimated the genetic heritability of 87 quantitative traits using GCTA (ver. 1.93.2) with “–reml –grm” options [28]. We estimated the GCs using the bivariate genome-based restricted maximum likelihood algorithm [50] in the GCTA (ver. 1.93.2) with “*–*reml-bivar –grm –reml-bivar-lrt-rg” options [28]. Two of the 253 trait pairs were excluded since the log-likelihood did not converge. The correction for multiple tests was done by a Benjamini–Hochberg approach when reporting confident GCs that suffice the threshold of an FDR below 0.05.

### Calculation of VSI

The VSI is a Jaccard score to measure how many pleiotropic components exist out of all significant variants from *i*th and *j*th traits, which is defined as


\begin{eqnarray*}
{\rm VSI}({\rm I,j}) = \left| {{\rm S}_{\rm i} \cap {\rm S}_{\rm j}} \right|/\left| {{\rm S}_{\rm i} \cup {\rm S}_{\rm j}} \right|
\end{eqnarray*}

where S_i_ and S_j_ denote sets of significant variants for the *i*th and *j*th traits, respectively. The VSI increases as 2 traits have more pleiotropic variants among their significant variants.

### Pleiotropic variants with tissue-specific expression regulatory function

We annotated the gene symbol of the pleiotropic variant by using the Ensemble database (ver. 101) [42]. In case of intergenic variants, we annotated the genes that were located the nearest in both directions of the variant. The single tissue eQTL data (RRID:SCR_013042, ver. 8) from the GTEx portal were used to investigate the eQTL of pleiotropic variants in Korea4K.

### Investigation of potential causal relationships between traits based on MR

We used the MR method to investigate potential causal relationships among 1,332 combinations of an exposure trait and an outcome trait among 37 clinical traits. MR is computed from the linear regression analysis between the effects of SNPs on an exposure trait and their effects on an outcome trait. We chose the SNPs with suggestive WGWAS results (*P* < 10^−5^) with exposure traits as the instrument variables. In case multiple SNPs existed in the LD block, the one with the smallest *P* value was chosen. We rejected 40 SNPs, which were detected as outliers of linear regression from MR-PRESSO software (RRID:SCR_023697, ver. 1.0) [51] with “NbDistribution=10000 and SignifThreshold=0.05” options, from further analysis. MR coefficients were computed using the chosen SNPs by 3 different methods: the inverse-variance weighted (IVW) and MR-Egger method of the TwoSampleMR package (RRID:SCR_019010, v.0.5.6) [52] and MR-PRESSO software (ver. 1.0) [51]. Finally, we selected 36 significant causal relationships that overlapped at least 2 of 3 methods (IVW, MR-Egger, and MRPRESSO). All analyses were performed with default options.

## Supplementary Material

giae014_GIGA-D-23-00109_Original_Submission

giae014_GIGA-D-23-00109_Revision_1

giae014_Response_to_Reviewer_Comments_Original_Submission

giae014_Reviewer_1_Report_Original_Submission Pui-Yan Kwok -- 6/15/2023 Reviewed

giae014_Reviewer_1_Report_Revision_1 Pui-Yan Kwok -- 12/31/2023 Reviewed

giae014_Reviewer_2_Report_Original_Submission Taras K Oleksyk, Ph.D. -- 8/31/2023 Reviewed

giae014_Reviewer_2_Report_Revision_1 Taras K Oleksyk, Ph.D. -- 12/6/2023 Reviewed

giae014_Supplemental_Files

## Data Availability

Allele frequency information of variants is publicly available under the Korea4K webpage [54]. Raw sequencing data, individual genotype information, and clinical trait data will be as easily and freely available as possible upon request and after approval from the Korean Genomics Center’s review board in UNIST. The raw sequencing data that can be distributed were uploaded to the European Genome-Phenome Archive under the study accession “EGAS00001007580.” Information about the Korean Genome Project and other data sharing can be found at the Korea4K webpage. All additional supporting data are available in the *GigaScience* repository, GigaDB [55].

## Additional Files

**Supplementary Fig. S1**. Variant batch effect of DNA sequences.

**Supplementary Fig. S2**. Variant distribution based on variant location and allele frequency category in Korea4K.

**Supplementary Fig. S3**. Power comparison of whole genome–wide association study between Korea4K and Korea1K.

**Supplementary Fig. S4**. Sequencing data quality metrics of Korea4K genomes.

**Supplementary Fig. S5**. QQ plots for the whole genome–wide association tests of the traits on the anthropometry category.

**Supplementary Fig. S6**. QQ plots for the whole genome–wide association tests of the traits on the blood circulation biochemical category.

**Supplementary Fig. S7**. QQ plots for the whole genome–wide association tests of the traits on the blood circulation physics category.

**Supplementary Fig. S8**. QQ plots for the whole genome–wide association tests of the traits on the diabetes category.

**Supplementary Fig. S9**. QQ plots for the whole genome–wide association tests of the traits on the electrolyte category.

**Supplementary Fig. S10**. QQ plots for the whole genome–wide association tests of the traits on the hearing test category.

**Supplementary Fig. S11**. QQ plots for the whole genome–wide association tests of the traits on the hematological category.

**Supplementary Fig. S12**. QQ plots for the whole genome–wide association tests of the traits on the hepatitis category.

**Supplementary Fig. S13**. QQ plots for the whole genome–wide association tests of the traits on the inflammation and etc. category.

**Supplementary Fig. S14**. QQ plots for the whole genome–wide association tests of the traits on the kidney function category.

**Supplementary Fig. S15**. QQ plots for the whole genome–wide association tests of the traits on the liver function category.

**Supplementary Fig. S16**. QQ plots for the whole genome–wide association tests of the traits on the pulmonary function category.

**Supplementary Fig. S17**. QQ plots for the whole genome–wide association tests of the traits on the thyroid function category.

**Supplementary Fig. S18**. QQ plots for the whole genome–wide association tests of the traits on the tumor biomarker category.

**Supplementary Fig. S19**. QQ plots for the whole genome–wide association tests of the traits on the urinalysis category.

**Supplementary Fig. S20**. QQ plots for the whole genome–wide association tests of the traits on the vision category.

**Supplementary Table S1**. Sample count in Korea4K.

**Supplementary Table S2**. Variant count in Korea4K before sample and variant filtering.

**Supplementary Table S3**. Variant count based on variant categories and reported to dbSNP.

**Supplementary Table S4**. Allele frequency information of populations for 62 Korean-specific missense variants.

**Supplementary Table S5**. Prediction of changes in protein thermodynamic stability according to missense variant.

**Supplementary Table S6**. List of the GWAS variants that have association significance *P* < 5E-8.

**Supplementary Table S7**. Genetic heritability measurement.

**Supplementary Table S8**. Genetic correlation measurement.

**Supplementary Table S9**. Phenotypic correlation estimation.

**Supplementary Table S10**. Pleiotropic variants.

**Supplementary Table S11**. Mendelian randomization results.

**Supplementary Table S12**. Statistics of clinical information.

**Supplementary Table S13**. Twenty-six traits with clinical calculations applied.

**Supplementary Table S14**. Statistical power measurement of 90 WGWAS tests.

## Abbreviations

1KGP: 1000 Genomes Project; 3D: 3-dimensional; AF: allele frequency; BMI: body mass index; CA19-9: carbohydrate antigen 19-9; CI: confidence interval; CODA: Clinical & Omics Data Archive; CRP: C-reactive protein; DNV: double nucleotide variant; EBI: European Bioinformatics Institute; eGFR: estimated glomerular filtration rate; FDR: false discovery rate; GC: genetic correlation; GCTA: genome-wide complex trait analysis; HDL: high-density lipoprotein; IBD: Identical by Descent; KGP: Korean Genome Project; LD: linkage disequilibrium; LDSC: linkage disequilibrium score regression; LPL: low-density lipoprotein; MR: Mendelian randomization; MT: mutant type; NGS: next-generation sequencing; PC: phenotypic correlation; PCA: principal component analysis; PheWAS: phenome-wide association study; QQ: quantile–quantile; SNP: single-nucleotide polymorphism; SNV: single nucleotide variant; SSV: suggestively associated variant; TNV: triple nucleotide variant; U_WBC: urine white blood cell; VEP: Variant Effect Predictor; VSI: Variant-Sharing Index; WGS: whole genome sequencing; WGWAS: whole genome–wide association study; WHtR: waist-to-height ratio; WT: wild type; WWtR: waist/weight ratio.
