# Supplementary material for: Korea4K: whole genome sequences of 4,157 Koreans with 107 phenotypes derived from extensive health check-ups
Source: Gigascience. 2024 Apr 16;13:giae014. doi: 10.1093/gigascience/giae014 (PMC11020240; doi:10.1093/gigascience/giae014)
Supplement: giae014_GIGA-D-23-00109_Revision_1 [file giae014_giga-d-23-00109_revision_1.pdf]

GigaScience

Korea4K: whole genome sequences of 4,157 Koreans with 107 phenotypes derived from extensive health check-ups

--Manuscript Draft--

Manuscript Number:	GIGA-D-23-00109R1
Full Title:	Korea4K: whole genome sequences of 4,157 Koreans with 107 phenotypes derived from extensive health check-ups
Article Type:	Research
Funding Information:	Ulsan National Institute of Science and Technology (1.200108.01) — Dr. Jong Hwa Bhak<br>Ulsan National Institute of Science and Technology (1.200047.01) — Dr. Jong Hwa Bhak<br>Small and Medium Business Administration (1425157253) — Dr. Jong Hwa Bhak<br>Small and Medium Business Administration (1425157301) — Dr. Jong Hwa Bhak<br>Small and Medium Business Administration (1425156792) — Dr. Jong Hwa Bhak<br>Ministry of Trade, Industry and Energy (20016225) — Dr. Jong Hwa Bhak
Abstract:	Background<br>Phenome-wide association studies (PheWASs) have been conducted on Asian populations, including Koreans, but many were based on chip or exome genotyping data. Such studies have limitations regarding whole-genome-wide association analysis, making it crucial to have genome-to-phenome association information with the largest possible whole-genome and matched phenome data to conduct further population-genome studies and develop healthcare services based on population genomics.<br>Results<br>Here, we present 4,157 whole-genome sequences (Korea4K) coupled with 107 health check-up parameters as the largest genomic resource of the Korean Genome Project. It encompasses most of the variants with allele frequency > 0.001 in Koreans, indicating that it sufficiently covered most of the common and rare genetic variants with commonly measured phenotypes for Koreans. Korea4K provides 45,537,252 variants, and half of them were not present in Korea1K (1,094 samples). We also identified 1,356 new geno-phenotype associations which were not found by the Korea1K dataset. Phenomics analyses further revealed 24 significant genetic correlations, 14 pleiotropic associations, and 127 causal relationships based on Mendelian randomization among 37 traits. In addition, the Korea4K imputation reference panel, the largest Korean variants reference to date, showed a superior imputation performance to Korea1K across all allele frequency categories.<br>Conclusions<br>Collectively, Korea4K provides not only the largest Korean genome data but also corresponding health check-up parameters and novel genome-phenome associations. The large-scale pathological whole-genome-wide omics data will become a powerful set for genome-phenome level association studies to discover causal markers for the prediction and diagnosis of health conditions in future studies.
Corresponding Author:	Jong Hwa Bhak, Ph.D.<br>Ulsan National Institute of Science and Technology<br>Ulsan, Ulsan KOREA, REPUBLIC OF
Corresponding Author Secondary Information:	
Corresponding Author's Institution:	Ulsan National Institute of Science and Technology

Powered by Editorial Manager® and ProduXion Manager® from Aries Systems Corporation

Corresponding Author's Secondary Institution:	
First Author:	Sungwon Jeon, Ph.D.
First Author Secondary Information:	
Order of Authors:	Sungwon Jeon, Ph.D.
	Hansol Choi, Ph.D.
	Yeonsu Jeon, Ph.D.
	Whan-Hyuk Choi, Ph.D.
	Hyunjoo Choi, Master of Science
	Kyungwhan An, Bachelor of Science
	Hyojung Ryu, Ph.D.
	Jihun Bhak, Bachelor of Science
	Hyeonjae Lee, Bachelor of Science
	Yoonsung Kwon, Bachelor of Science
	Sukyeon Ha, Bachelor of Science
	Yeo Jin Kim, Ph.D.
	Asta Blazyte, Master of Science
	Changjae Kim, Ph.D.
	Yeonkyung Kim, Master of Science
	Younghui Kang, Bachelor of Science
	Yeong Ju Woo, Bachelor of Science
	Chanyoung Lee, Bachelor of Science
	Jeongwoo Seo, Bachelor of Science
	Changhan Yoon, Master of Science
	Dan Bolser, Ph.D.
	Orsolya Biro, Ph.D.
	Eun-Seok Shin, M.D., Ph.D.
	Byung Chul Kim, Ph.D.
	Seon-Young Kim, Ph.D.
	Ji-Hwan Park, Ph.D.
	Jongbum Jeon, Ph.D.
	Dooyoung Jung, Ph.D.
	Semin Lee, Ph.D.
	Jong Hwa Bhak, Ph.D.
Order of Authors Secondary Information:	
Response to Reviewers:	GIGA-D-23-00109<br>Korea4K: whole genome sequences of 4,157 Koreans with 107 phenotypes derived from extensive health check-ups<br>Sungwon Jeon; Hansol Choi; Yeonsu Jeon; Whan-Hyuk Choi; Hyunjoo Choi; Kyungwhan An; Hyojung Ryu; Jihun Bhak; Hyeonjae Lee; Yoonsung Kwon; Sukyeon Ha; Yeo Jin Kim; Asta Blazyte; Changjae Kim; Yeonkyung Kim; Younghui Kang; Yeong Ju Woo; Chanyoung Lee; Jeongwoo Seo; Changhan Yoon; Dan Bolser; Orsolya Biro; Eun-Seok Shin; Byung Chul Kim; Seon-Young Kim; Ji-Hwan Park; Jongbum Jeon;

Powered by Editorial Manager® and ProduXion Manager® from Aries Systems Corporation

Dooyoung Jung; Semin Lee; Jong Hwa Bhak
GigaScience

Reviewer reports:

Reviewer #1: This manuscript describes the second phase of the Korean Genome Project (KGP) with 4,157 sets of whole-genome data (designated Korea4K). After error correction and sequencing data curation, the whole-genome sequencing (WGS) data from 3,614 unrelated were used in the analyses. They also analyzed 107 types of clinical traits from 2,685 healthy participants' health check-up reports over a 4-year period (2016-2019). They performed a range of analyses and claimed that this new data performed better than Korea1K, the first phase KGP dataset, in a number of ways. A larger Korean dataset adds to the global genome resource and provides further insights into the Korean population. However, the results are mostly descriptive and serve as a catalog without significant new insights. The results are as expected (Korea4K is a better imputation reference panel than Korea1K, new variants are identified in the population, new variants are found in association with various phenotypes, etc.) and this dataset is sufficiently large to capture all the common variants found in the homogeneous Korean population.
The authors should address several issues:

1. The use of whole genome sequencing data in GWAS. The Bonferroni correction the authors used in their analysis was that for SNP array studies. They must do a formal correction with the many more variants found in WGS data and use a statistically sound correction for their analysis. The severe penalty for multiple testing using WGS data for GWAS is why few such studies have been done. I suspect that many of the associations will not reach statistical significance after proper correction, as the dataset is quite small for most traits under study.

⇒ Thank you for your critical comments regarding the statistics on the GWAS results. As you pointed out, we agree that there should be a stricter correction. As one method, we have now employed the FDR correction (Benjamini-Hochberg) which can remove possible false positives. The FDR values for each variant are now included in Supplementary Table S6 (List of the GWAS variants which have association significance P < 5E-8). After the FDR correction, 2314 variants from 30 traits still maintained statistically significant associations (FDR <0.05). We additionally noted the number of remaining variants and traits according to different FDR cutoffs in Table R1 below. Also, the results of the FDR correction were updated in the manuscript.

Page 11: “Among the significantly associated variants, 2,314 variants from 30 clinical traits still showed significance after false discovery rate (FDR) correction using the Benjamini-Hochberg approach (FDR < 0.05).”

Table R1. Number of significantly associated variants and traits according to FDR cutoffs

FDR cutoff	Number of variants	Number of traits
FDR < 0.1	2320	31
FDR < 0.05	2314	30
FDR < 0.01	2256	24
FDR < 0.005	2193	24
FDR < 0.001	1916	18

2. The authors should use the new genome references for their variant calling (T2T reference and the Human Pangenome Reference), as the GRCh38 is no longer the gold standard, and the results will be quite different with the most up-to-date references. Using the best human genome reference will make Korea4K more valuable.

⇒ We agree that we could potentially find more genetic markers that are related to the traits in our GWAS analysis, for example, when we use the T2T reference or the

Powered by Editorial Manager® and ProduXion Manager® from Aries Systems Corporation

Human Pangenome Reference. However, there are a few considerations that limit us from using these references:

The T2T reference lacks enough annotation data which is critical. Many major genomic databases, such as dbSNP, are based on GRCh38. Thus, even if we were to use the T2T reference, we would have limitations in interpreting or validating the variants/markers we could additionally discover.

The draft Human Pangenome reference genome contains genomic sequences of 47 genetically diverse individuals, which requires a totally different bioinformatics pipeline to analyze. The bioinformatics analysis using the Human Pangenome reference is not fully established currently, which means that the validation method of the genetic markers that could be discovered should also be investigated more. Also, the Human Pangenome reference was assembled based on long-read sequencing data such as PacBio and Oxford Nanopore Technologies (ONT). As the authors of the Human Pangenome reference paper mentioned, the 1-base level of the sequencing accuracy can be an issue, which makes it hard to know if additional discoveries using the Pangenome are true signals or artifacts.

Furthermore, mapping the whole-genome sequencing reads for 4K samples and jointly genotyping the variants technically requires more than a year of time to rebuild the dataset.

We understand the importance of more precise and complete genome references for the variant calling and we appreciate your suggestion. We will expand the variant call set using the T2T and Human pangenome references in our future studies. Unfortunately, as we mentioned above, due to several technical limitations, we were not able to apply the new genome references in our current study, although we revised the manuscript to add the importance of the usage of these references.

Page 22: “Moreover, utilizing recently introduced human genome references like the T2T reference [33] and Human Pangenome reference [34], which offer broader genomic coverage or have population-specific sequences compared to the existing GRCh38 reference, could help identify additional associations that might be overlooked. Nevertheless, these new references lack functional annotations and need to be connected to previous databases such as dbSNP and the GWAS catalog.”

3. The authors should clarify how many of the participants who contributed clinical data are unrelated.

⇒ As described in the Methods, we filtered out a total of 540 individuals including 428 samples that have relatedness to other samples from 4,157 samples. Among the final unrelated 3,617 samples, 2,262 samples had clinical data. We updated the manuscript to provide a clear description of the participants who contributed to the clinical data.

Page 28: “Out of the final unrelated 3,617 samples, 2,374 samples had clinical data available and were included in the phenomics analyses.”

Reviewer #2: The authors contribute 4,157 whole-genome sequences (Korea4K) coupled with 107 health check-up parameters as the largest genomic resource of the Korean Genome Project. It has likely characterized most of the common and very common genetic variants with commonly measured phenotypes for Koreans. It also discusses its applicability not only for the Korean population but also for other East Asian populations, and possibly to other national genome projects as well.

This work makes a significant contribution of data that can be used in future genome-wide association studies in the context of the Korean population. The manuscript appears to cover a lot of ground: from methodological issues to the real-world applications of the dataset in healthcare. The authors adopt innovative methods like GREML, which have been reported to have higher accuracy compared to older

methods.

The authors are transparent about the limitations of their study, such as sample size and lack of sufficient data for rare diseases. They also acknowledge that phenomics analyses were not powerful enough for novel discoveries, indicating areas for future research. However, given the increasing importance of genomic data in healthcare and personalized medicine, the paper appears to be highly relevant.

While the paper is well formulated, there are some issues that need to be addressed before is accepted for publication.
See below:

1. You referred to the UK Biobank data for some of your analyses. Were there any limitations or caveats in comparing your dataset to the UK Biobank? What about other national genomic projects that are out there? How transferable do you think the Korea4K dataset would be to studies focusing on other populations outside East Asia?

⇒ Yes, there can be many limitations/caveats. However, please take in consideration that we cannot report the limitations precisely since we have not fully utilized the raw genomic and extensive clinical data from the UK Biobank but only the GWAS summary data in the current study.

One clear limitation is the difference in allele frequencies of reported variants between two ethnic groups in comparison due to different population genomic structures. This has been demonstrated in our PCA results (please refer to Figure 2). Even if the two populations were to have the same sample size, disparities in the allele frequencies would lead to different summary statistics (i.e., beta- and p-values). Therefore, the downstream, namely the phenomic, analysis could suffer from different resultant statistics and a fair comparison between the two independent studies could be difficult for some trait pairs.

Another possible limitation is that the current GWAS summary data provided by the UK Biobank (and other national biobanks) is based on arrays and many of the variants are imputed genotypes. Here, we have utilized the whole-genomes. We found that the GWAS-significant variants in comparison are not often overlapping due to technical biases as reported in our prior study (Jeon YS et. al., 2023, Hum Genet.). A possible solution to this would be to perform the joint genotyping of the Korean whole-genomes in conjunction with the UK Biobank whole-genomes. However, the entire process is resource-intensive and time-consuming, such that only the institutes with sufficient computing power will be able to process the data. In addition, rare variants appearing in each ethnic group will be grossly undermined.

The content and amount of phenotypic and clinical information provided are another possible limitation. Our health check-up data have been collected from multiple sources/centers such that we had to process the heterogeneous physical and digital copies of the health records and standardize them. On the other hand, all the participants in the UK Biobank were sampled in a single-centered manner with a unified procedure. Hence, our phenotypic and clinical information is much more limited than the UK Biobank's, and the method of measurements may differ for a few specific categories although we did not deeply investigate.

Furthermore, Korea4K data is not as readily accessible as the UK Biobank data. Obtaining it requires navigating through IRB processes and administrative procedures, and the legislative framework in South Korea does not currently facilitate a straightforward download process. If improved in the future, we will be able to make them more accessible. As an initial step toward enhanced accessibility, we have deposited our dataset in the European Genome-Phenome Archive (EGA) under the study accession 'EGAS00001007580' and are actively working towards providing the WGS data openly and freely.
Moreover, we have included the accession number in the manuscript to facilitate easy reference.

Page 34: "The raw sequencing data that can be distributed were uploaded to the European Genome-Phenome Archive under the study accession

‘EGAS00001007580’.”

2. Could you expand on any ethical considerations that were taken into account, especially in terms of data privacy and informed consent?

⇒ Yes. In our project, we have taken ethical considerations, particularly concerning data privacy and informed consent, very seriously. The data we generated and used in our study comes from blood or saliva donations, and we have obtained explicit, full consent forms from the participants before getting the samples. These consent forms ensure that the participants are aware of how their data will be used and that they have willingly agreed to share this data for research purposes and IRB. As a result, we can make the data of 3,839 individuals publicly available while respecting the privacy and consent of the participants.

We have expanded the ethical considerations in “Ethics, consents and permissions”:

Page 34: “The data employed in our study originates from voluntary blood or saliva donations, and we have diligently secured explicit, comprehensive consent forms from all participants prior to sample collection. These consent forms explicitly outline the intended use of their data for research purposes and underscore the voluntary nature of their participation. Furthermore, our study adheres to the ethical guidelines and regulations stipulated by the IRB. As a result, we can make the data of 3,839 individuals publicly available while respecting the privacy and consent of the participants.”

3. How was the data cleaned and preprocessed, and were there any missing data points? If so, how were these handled? What number of reads(before and after QC), and other quality metrics do the sequenced reads have? What was the average coverage across the genome? What was the read length?

⇒ We cleaned and preprocessed by trimming possible adapter contamination on the sequencing reads and made the reads have at least 50bp of read length using the Cutadapt program (ver. 1.9.1). Then we confirmed the read counts, quality, and amount of bases using the FASTQC program. The average sequencing depth was 27.75 × and initial read lengths were 151bp. However, it varied after the trimming. The number of reads, average coverage, average quality, and filtering percentage were visualized in Supplementary Figure S4 and we updated the preprocessing procedure in the method section of the manuscript.

Page 23: “All the sequencing data that we used in this study had 151bp as a read length. Average sequencing amount per sample was 20× (Supplementary Figure S4).”

Page 24: “Adapter contamination was trimmed using Cutadapt (RRID: SCR_011841, ver. 1.9.1) [35] with a forward adapter (′GATCGGAAGAGCACACGTCTGAACTCCAGTCAC′) and reverse adapter (′GATCGGAAGAGCGTCGTGTAGGGAAAGAGTGT′) and with a minimum read length of 50 bp after trimming (Supplementary Figure S4).”

Page 24: “A total of 3,156 samples had a mapping depth of ≥ 20× (Supplementary Figure S4).”

4. How did you ensure the quality of the genomic data collected from different sources such as Korea1K and public data archives?

⇒ To ensure the quality, we rigorously utilized a standardized pipeline for processing the genomic data and performed batch effect removal.

We applied the same bioinformatics analysis and QC pipelines as in the Korea1K

Powered by Editorial Manager® and ProduXion Manager® from Aries Systems Corporation

(Jeon S et. al., 2020, Sci. Adv.). For example, we ensured the same version of the programs, and the same parameters through all the WGS data when we genotyped the samples.

We collected the WGS data from multiple sources or sequenced the whole genomes at different time points, which could suffer from the batch effect. After jointly genotyping the WGS data, we tried to reduce the batch effect from the different sequencing batches. As we noted in the manuscript, we applied the allele balance-based variant filtering.

The paper mentions mitigating batch effects through allele balance and manual checks. Could you provide more details on the methodology behind these checks and their efficiency?

⇒ As for the manual checks, we filtered out the undesired samples based on the following categories:
(1) high missing genotype rate (>10%);
(2) outlying heterozygous variants ratio (3 s.d.);
(3) high relatedness;
(4) non-Korean genetic background from PCA;
(5) having a rare disease;
(6) samples no longer available.

As for the allele balance, we first measured the average allele balance of the genotyped alleles (the read count of the allele divided by the total read count on a locus). Then, we excluded 12,713,580 variants that had an average allele balance of the loci out of the range of ± 1 × standard deviation (SD) from a genome-wide average of allele balance to remove the sequencing batch effect. We confirmed that the batch effects were removed after the filtering and visualized it as PCA plots (Supplementary Figure S1).

We have now added the definition of the allele balance in our Methods:

Page 25: “To detect variants which were probably called because of a sequencing batch effect, we measured average allele balance of the genotyped alleles (the read count of the allele divided by the total read count on a locus).”

5. Could you provide more information about the control group? Was it matched for age, sex, or other variables?

⇒ In the Korea4K dataset, we do not have a specific control set. We conducted our GWAS analysis only for the quantitative clinical traits using a linear regression approach without separately categorizing the study participants into case and control groups. Age, Sex, and BMI were included as covariates in testing the significance of variants across the clinical variables of interest.

How was the sample size determined, and does it provide enough statistical power to support your conclusions?

The sample size employed for each trait was maximized by utilizing the available samples in the Korea4K. Regarding the statistical power of GWAS, we recognize the importance of ensuring an adequate sample size to obtain robust results. We calculated the statistical power and effect size based on a likelihood ratio test by the R package (“genpwr”). Out of the 90 WGWAS traits analyzed, a majority of traits (77 traits) exhibited enough statistical powers exceeding 80% under the assumption of an effect size of 0.5 and a minor allele frequency (MAF) of 0.01 (added to Supplementary Table S14). Given the sufficient statistical power for the majority of traits examined. Our study’s sample size of 4,157 individuals was appropriate for addressing the research objectives.

The detailed method is added to the manuscript:

Powered by Editorial Manager® and ProduXion Manager® from Aries Systems Corporation

Page 29: “Statistical powers of the 90 WGWAS were calculated by the R package “genpwr” under the assumption of an effect size of 0.5 and a minor allele frequency of 0.01 (Supplementary Table S14).”

6. You mentioned that the statistical power of your study will increase with more participants. Would this have implications for other national genomes that are making similar projects?

⇒ Probably yes and no. Yes, for other populations which are as homogenous as Koreans. The current study is very specific in that the population is very genetically homogenous. Koreans are probably the most homogenous population in East Asia. The diversity is even less than the Japanese archipelago (and the mainland China) because the Korean peninsula has been geographically isolated compared to other islandic populations. No, if a population is extremely heterogeneous with mixed ethnicities, the statistical power of our analysis would be much lower with the same sample size. In general, the statistical power will increase as the sample size goes up as you already know. However, we are unsure if the behavior would be exactly as our claim, and further studies are warranted.

Please elaborate on how your sensitivity analysis could apply to other populations outside Korea.

⇒ Thank you for a very interesting question. We find it very hard to answer because as mentioned above the Korean population is extremely homogeneous. It is a special population in terms of genetic and environmental diversity. Therefore, it is very difficult to estimate what kind of insight we may apply to other populations outside Korea. It could be an advantage or a disadvantage depending on the application of the population.

7. The paper acknowledges the sample size as not sufficiently large for detecting weak associations and admits that the sample size was not large enough to detect weak association signals. Have you considered statistical methods that can boost power in small samples?

⇒ We appreciate your comment. We could think of two statistical methods to improve statistical power: Meta-analysis and Gene-based association test. 1) Meta-analysis is one of the methods to boost statistical power for small samples by combining GWAS summary statistics of multiple independent studies. However, our study focuses specifically on the whole-genome-wide associations in the Korean population. Although a meta-analysis could improve the sensitivity of the weak association signals, the analysis might be able to introduce potential biases due to the genetic heterogeneity across populations. Thus, we have focused on our analysis of the Korean population. The Korean population’s genetic characteristics and their implications for clinical trait associations would provide an important aspect of our study, providing a unique and valuable. 2) Gene-based association test is another approach that increases the power to detect associations. This method aggregates the effects of multiple genetic variants within a gene to assess their collective impact. While the method is particularly advantageous for analyzing rare variants, we focused on identifying common variants associated with clinical traits in the Korean population. The application of gene-based association tests to our study could potentially yield additional insights, especially in the context of rare variant associations. We acknowledge the value of this approach and are considering its application in our subsequent study with a larger sample size.

8. Could you provide more details on the 107 clinical parameters used for the Korea4K phenome dataset? Were these parameters standardized across the different clinics and hospitals?

⇒ Yes, we standardized the parameters across the different clinics. For example,

Powered by Editorial Manager® and ProduXion Manager® from Aries Systems Corporation

discrepancies in unit measurements, such as micrograms (ug) and nanograms (ng), were unified for specific traits, posing a direct challenge to the analytical process if these variations were not duly reconciled. Also, some parameters such as e-GFR were calculated by different equations across the clinics. We re-calculated such parameters using a singular formula. More detailed methods were updated in the method section of the manuscript and Supplementary Table S13.

Page 28: “In the context of collecting data from over 200 diverse healthcare institutions, standardizing clinical information on 107 traits became imperative. We resolved discrepancies in unit measurements, such as micrograms and nanograms, for specific traits. Furthermore, certain clinical metrics, such as the estimated glomerular filtration rate (e-GFR), were found to exhibit variability contingent upon variables such as ethnicity, sex, and age. To maintain consistency and ensure methodological uniformity, we enforced the adoption of a singular clinical formula for the computation of e-GFR across all data samples. Such calculations were applied to 26 traits (Supplementary Table S13). Clinical traits that exhibited values characterized by inequalities likely due to the limit of detection (e.g., <5.0 and >99) were omitted from the analytical procedures, as such values have the potential to introduce disturbances to subsequent data analyses. Likewise, values that exhibited divergent formatting conventions across distinct healthcare institutions (e.g., 20 and a few or 999 and many) were harmonized to conform with prevailing standard criteria observed in most samples under investigation.”

9. What criteria were used for initial sample filtering, particularly for excluding kinship? Could you clarify the steps taken to identify and filter the 64,301,272 SNVs and 8,776,608 Indels? How did you correct for batch effects arising from different Illumina NGS platforms and library preparations? Did you use specialized SNV calling software, or only GATK?

⇒ We have briefly mentioned this in our method section, we filtered out total 540 samples and the detailed filtering steps are noted in the method section as well. To exclude the samples who were in the kinship relationship, we first measured IBD values between the samples using Plink program (ver. 1.90b3n) and defined the family trees based on the IBD values which showed a PI_HAT value more than 0.05. Then, we filtered out samples in a family tree to have the maximum number of the remaining samples.

We also updated the manuscript to provide detailed procedures for defining the kinship relation between samples:

Page 25: “To explore kinship relations among the samples, we assessed Identical by Descent (IBD) using the Plink program (RRID:SCR_001757, ver. 1.90b3n) [30]. Samples with a PI_HAT value exceeding 0.05 were considered to be in a kinship relation.”

About the variant calling, we did not use specialized SNV calling software. As we noted in the method section, we jointly genotyped the genotypes using only GATK 4.1.3 and identified the 64,301,272 SNVs and 8,776,608 Indels. With the jointly genotyped data, we measured allele balance of the loci. If the average allele balance of the loci was out of the range of $\pm 1 \times$ standard deviation (SD) from a genome-wide average of allele balance, the loci were treated as generated by possible batch effects due to different Illumina NGS platforms and library preparation and filtered out using an in-house script. A similar method was previously suggested by Muyas F, et. al., 2019. Following the filtering method, we excluded 12,713,580 variants and confirmed that the batch effects were removed through the PCA plots in Figure S1.

10. How were allele frequencies calculated and what considerations were made to interpret their biological significance? You mention that more than half of the singleton and doubleton variants were newly discovered. Could you elaborate on the methodology used to confirm these as novel variants?

⇒ The allele frequencies were calculated by the number of alternative alleles divided by the number of called alleles in a position. Generally, the allele frequency distribution can reflect the genomic diversity of the population. The definition we used to assign the variant as a novel variant is whether the variant was reported in the dbSNP database or not. We updated the figure legend of Figure 1 of the manuscript to provide the definition.

Page 9-10: "dbSNP indicates the variants were reported in dbSNP database. Novel indicates the variants were not reported in dbSNP."

11. The section on phenotypic correlations mentions 2,274 trait-trait relationships. How would you address the potential for population stratification affecting the results of your genetic and phenotypic correlations?

⇒ The problem of population stratification in GWAS especially arises when conducting GWAS in multi-ethnic countries or meta-analyses across multiple sources of data with mixed ancestries. Here, our study exclusively deals with samples of Korean ancestry, which means our dataset is genetically homogeneous compared to other studies. To make sure, we excluded any samples with non-Korean genetic backgrounds based on PCA analysis as we noted in the "Sample and variant filtering" section in the Methods. Also, we included PC1~10 as covariates in our regression models during GWAS to avoid any latent ancestral effects from differential ethnic subgroups as we noted in the "Whole genome-wide association study (WGWAS)" section in the Methods.

We argue that such factors may add to reducing spurious correlations introduced by population stratification. Our claim is supported by values for the genomic inflation factor (λMedian) (Supplementary Figure S4-19). As you may already know, the value of lambda below 1.1 is generally considered acceptable indicating minimal false positives caused by gross population structure (and systematic biases) (please refer to Yang et. al., 2011, Eur J Hum Genet.)

We have now included the calculation of genomic inflation factor to estimate the gross population structure (and systematic biases) in our data.

Page 29: "Calculating the genomic inflation factor (λMedian), we found that all of the traits in the test reside below 1.1 indicating there are minimal false positives caused by gross population structure or systematic biases (Supplementary Figure S4-19) [48]."

How did you account for multiple comparisons in determining significant genetic correlations, and what corrections were applied to maintain the FDR?

⇒ We see that we were insufficient in our details as to how we corrected for multiple comparisons while calculating genetic correlation. We put our best effort to avoid false discoveries by conducting Benjamini-Hochberg correction to maintain the FDR well and below 0.05. Consistently, we applied the same correction method for setting FDR for phenotypic correlation as well.

We have now added the Methods for multiple testing correction for phenotypic correlation and genetic correlation:

Page 29: "Benjamini-Hochberg method was used to adjust for multiple comparisons when documenting confident phenotypic correlations with FDR."

Page 30: "The correction for multiple tests was done by Benjamini-Hochberg approach when reporting confident GCs that suffice the threshold of FDR below 0.05."

What measures were taken to ensure that the traits considered in this section were not

subject to confounding and/or collider biases.

⇒ Thanks for pointing the important question. Confounding and collider biases are unavoidable when looking at multiple associations across numerous variables at once. First, we employed covariate adjustment to reduce confounding biases by traits that are highly correlated with other clinical variables. We focused on Age, Sex, and BMI which have previously been suggested by Shungin et al. (2015) (Shungin D et. al., 2015, Nature). Second, we incorporated Mendelian Randomization (MR). MR is a statistical method to ascertain the direction of effect and imply possible causality devoid of confounders and colliders (Mitchell RE et. al., 2023, PLOS Genetics, and Ebrahim S and Davey Smith G, 2008, Human Genetics). To raise the confidence of our claim, we utilized three independent MR methods, namely IVW, MR-Egger, and MR-PRESSO, and documented the causal relationships if at least two of three were shown significant as noted in the Methods. However, we acknowledge that our methods of MR might still be prone to a collider bias, especially due to conditioning by covariates, which was to remove confounders and increase the power (Cai S et. al., 2022, Genetic Epidemiology). However, we would like to emphasize that such bias has minimal effect on the interpretation of phenotypic associations as previously reported by Pulit et al. (2019) (Pulit SL et. al., 2019, Hum Mol Genet).

We have added our responses to your comment into the Methods and Results, respectively:

Page 29: “Age and BMI were chosen especially due to their known shared associations with multiple traits as previously documented by Shungin and colleagues which could lead to confounding biases in the downstream interpretation of phenotypic relationships [47].”

Page 16: “In addition to the investigation on the general pleiotropic relationship, we employed Mendelian Randomization (MR) to detect vertical pleiotropy that can assert the direction of the phenotypic relationships [18]. This provides indirect evidence implying causality between the traits to discern spurious phenotypic associations, such as confounding and collider bias [19, 20].”

12. In your findings, Waist-Creatine showed opposite directions for genetic and phenotypic correlations. Could you elaborate on the potential implications or causes of this discrepancy?

⇒ Thanks for raising this point so that we could put attention here for a richer discussion. We suggest the discrepancy mainly comes from the shared environmental factors between Waist and Creatinine. This is well-reviewed by Sodini et al. (2018) (Sodini SM, et. al., 2018, Genetics). Most easy-to-understand case would be that from the dietary habits of individuals. It is well-known that the “meaty diet” readily elevates the serum creatinine level as well as the waist circumference (Khodayari S et. al., 2022, BMC Research Notes, and Pimenta E et. al., 2016, J Clin Epidemiol.). Hence, the higher the meat consumption, the higher the creatinine and wider the waist will be - a positive phenotypic correlation induced by the confounding effect of meat ingestion. We argue that the environmental effect would be exaggerated considering the relatively low heritability estimate of Creatinine.

We have now added the implication in our Results section:

Page 14: “Such discrepancies between the correlation estimates are possibly derived from the shared environmental factors between a pair of traits, such as dietary habits, that overwhelm the genotypic effects [12, 13]. This proves that the phenotypic correlation is not a mere proxy for the genetic correlation and consideration on the environmental effect is indispensable for the accurate interpretation of human phenomics [14].”

13. Were there any other surprising or unexpected correlations, and what are their

	potential implications?<br><br>⇒ Yes, there were a few surprising correlations, and we would like to emphasize the following:<br><br>1)Utility of Secondary Body Measures: WHtR, WWtR, BMI<br>WHtR (Waist-to-height Ratio) and WWtR (Waist-to-weight Ratio) are secondary body measures that come from combining two bodily measures, similar to Body Mass Index (BMI). Our phenomics results also depict distinguishable patterns of association between these secondary body measures with other phenotypes. WHtR has a causal relationship with the C-reactive protein (CRP), Fat percentage, and HDL. On the other hand, WWtR showed associations with measures of lung capacity (FEV1 and FVC), liver function (AST), and inflammation (U_WBC). BMI positions as an intermediate phenotype, largely sharing its associations with WHtR and lightly with WWtR via left naked eyesight. These may reflect distinct biological mechanisms between the measurements warranting further studies. For example, WHtR is a well-known indicator of central adiposity, which serves as a better estimate obesity and related morbidities than BMI (Lee CMY et. al., 2008, J Clin Epidemiol.).<br><br>2)Complementary markers for cancer diagnosis: ALP and Amylase<br>CEA is a well-known biomarker for colon and lung cancer, while CA125 for ovarian. We found two independent causal relationships from these cancer biomarkers with other serum proteins that are seemingly irrelevant to cancerous phenotypes. Interestingly, our result suggests that alkaline phosphatase has influence on CEA. This implies that inflammation and dysfunction in the organs, such as liver or colon, precedes the alteration in the level of the cancer biomarker. It may be possible that ALP and CEA can both be used for detecting the presence of cancerous cells. Many early studies have reported their value in diagnosing cancer and monitoring metastasis in various types of cancer, including liver and colon, validating our finding (Aabo K et. al., 1986, Eur J Cancer Clin Oncol., Tartter PI et. al., 1981, Ann Surg., and Walach N et. al., 1989, J Surg Oncol.). Similarly, we could establish relationship between serum Amylase and CA125. Interestingly, we found cis-eQTLs underlying their pleiotropy are associated with the level of AMY2B expression in pancreas. Surprisingly, there have already been reports that patients with ovarian cancer manifest hyperamylasemia (Guo S et. al., 2018, Medicine, Shintani D, 2016, Eur J Gynaecol Oncol, and Zakrzewska I and Pietryńczak M, 1995). We argue that serum Amylase can be used as a complementary marker for ovarian cancer similar to ALP.<br><br>We elaborated our interesting correlations and their implications both in the Results and Discussion:<br><br>Page 18: “In our casual diagram (Figure 5, blue arrows), ALP and CEA showed potential causality, along with the shared genetic variants between them (pleiotropy near ABO gene). Numerous previous studies have consistently reported these markers together for diagnosing cancer and monitoring metastasis [21-23]. Similarly, CA125 and Amylase also displayed causality via shared genetic variants (pleiotropy near AMY2B gene). We propose that CA125 and Amylase might serve as complementary biomarkers for ovarian cancer, much like ALP and CEA. The biological relationships between these clinical blood measures remain unclear.”<br><br>Page 18: “Our phenomics results also depicted distinguishable patterns of association between secondary body measures, such as WHtR (Waist-to-Height Ratio), WWtR (Waist-to-Weight Ratio), and BMI (Body Mass Index), with other phenotypes. WHtR exhibited a causal relationship with CRP (C-reactive protein), body fat percentage, and HDL. The result is concordant with previous reports that body fat percentage and CRP are correlated [24, 25]. Conversely, WWtR had casual associations with measures of lung capacity (FEV1 and FVC), liver function (AST), and inflammation (U_WBC). However, WWtR has yet to be proven its utility in clinical studies. BMI serves as an intermediate phenotype, sharing most of its associations with WHtR and, to a lesser extent, with WWtR via left naked eyesight. These findings suggest that the measurements reflect distinct biological mechanisms, warranting further studies. For instance, WHtR is a well-known indicator of central adiposity which provides a better estimate of obesity and related morbidities than BMI [26].”

Powered by Editorial Manager® and ProduXion Manager® from Aries Systems Corporation

	Page 20: “Nevertheless, our findings bear important practical implications. We described the utility of secondary body measures, such as WHtR and WWtR, compared to BMI. We also elaborated on the diagnostic and prognostic value of other serum proteins, namely ALP and Amylase, in conjunction with the existing cancer biomarkers.”<br><br>14. You mentioned that phenomics analyses were not powerful enough for novel discoveries. Could you elaborate more on what would be needed to make them more effective?<br>⇒ Yes, we can elaborate on a few points of improvement for a more effective study in the future. The current dataset of the Korea4K includes 4,157 healthy samples with no apparent disease onset at the time of collection. Therefore, we could not see if our clinical variables, found from the phenomics analyses, have association on pathological conditions that is medically important. In future, we could use a wider variety of health-related categories to conduct a more powerful study, validating the current results with an enhanced scope which would bear invaluable medical and practical implications. Furthermore, collection of more samples for sequencing and health record data is also required for better chance of discovering new relationships. We have added the following sentence in our Discussion:<br>Page 21: “However, we plan to collect more samples for sequencing and health record data with a wider variety of health-related categories to conduct a more powerful study in the future. This will allow us to not only validate our findings but also find correlations of medical importance that were missed in the present study.”<br><br>15. For the future implications, in terms of healthcare and personalized medicine, what do you see as the most immediate applications of the Korea4K dataset?<br><br>⇒ Thank you for asking these important points. As we mentioned in "Potential Implications”, the Korea4K dataset contains both whole-genome scale genotypes and matched clinical information. Thus, the dataset can immediately be applied to discover novel genetic markers that are associated with several phenotypes, diseases, or drug responses for Korean and East Asians. As a reference panel, the expanded genotype dataset (1K to 4K) can support more accurate genotyping imputation which is essential for DNA chip-based genotyping that is still widely used for healthcare (such as genetic tests). Furthermore, the Korea4K dataset can be used as a control data set across many different studies if the proper control samples are not applicable.
**Additional Information:**	
**Question**	**Response**
Are you submitting this manuscript to a special series or article collection?	No
**Experimental design and statistics**<br><br>Full details of the experimental design and statistical methods used should be given in the Methods section, as detailed in our Minimum Standards Reporting Checklist. Information essential to interpreting the data presented should be made available in the figure legends.<br><br>Have you included all the information requested in your manuscript?	Yes

Powered by Editorial Manager® and ProduXion Manager® from Aries Systems Corporation

**Resources**<br><br>A description of all resources used, including antibodies, cell lines, animals and software tools, with enough information to allow them to be uniquely identified, should be included in the Methods section. Authors are strongly encouraged to cite Research Resource Identifiers (RRIDs) for antibodies, model organisms and tools, where possible.<br><br>Have you included the information requested as detailed in our Minimum Standards Reporting Checklist?	Yes
**Availability of data and materials**<br><br>All datasets and code on which the conclusions of the paper rely must be either included in your submission or deposited in publicly available repositories (where available and ethically appropriate), referencing such data using a unique identifier in the references and in the "Availability of Data and Materials" section of your manuscript.<br><br>Have you have met the above requirement as detailed in our Minimum Standards Reporting Checklist?	Yes

Manuscript

Click here to access/download;Manuscript;Korea4K_Manuscript_GS_202403

Korea4K: whole genome sequences of 4,157 Koreans with 107 phenotypes derived from extensive health check-ups

Authors

Sungwon Jeon[1,2,†], Hansol Choi[1,3,†], Yeonsu Jeon[1,2], Whan-Hyuk Choi[1,3,4], Hyunjoo Choi[1,3], Kyungwhan An[1,3], Hyojung Ryu[1,2], Jihun Bhak[1,3], Hyeonjae Lee[1,3], Yoonsung Kwon[1,3], Sukyeon Ha[1,5], Yeo Jin Kim[2], Asta Blazyte[1,3], Changjae Kim[2], Yeonkyung Kim[2], Younghui Kang[1,2], Yeong Ju Woo[2], Chanyoung Lee[1,3], Jeongwoo Seo[1,3], Changhan Yoon[1,3], Dan Bolser[6], Orsolya Biro[7], Eun-Seok Shin[8], Byung Chul Kim[2], Seon-Young Kim[9], Ji-Hwan Park[9], Jongbum Jeon[9], Dooyoung Jung[3], Semin Lee[1,3,*], and Jong Bhak[1,2,3,10,*]

[1]Korean Genomics Center (KOGIC), Ulsan National Institute of Science and Technology (UNIST), Ulsan 44919, Republic of Korea

[2]Clinomics Inc., Ulsan 44919, Republic of Korea

[3]Department of Biomedical Engineering, College of Information-Bio Convergence Engineering, Ulsan National Institute of Science and Technology (UNIST), Ulsan 44919, Republic of Korea

[4]Department of Mathematics, Kangwon National University, Chuncheon 24341, Republic of Korea

[5]Department of Computer Science & Engineering (CSE), College of Information-Bio Convergence Engineering, Ulsan National Institute of Science and Technology (UNIST), Ulsan 44919, Republic of Korea

[6]Geromics Ltd., 222 Mill Road, Cambridge, CB1 3NF, United Kingdom

[7]Clinomics Europe Ltd., Budapest 1094, Hungary

[8] Department of Cardiology, Ulsan University Hospital, University of Ulsan College of Medicine, Ulsan, 44033, Republic of Korea
[9]Korea Bioinformation Center, Korea Research Institute of Bioscience and Biotechnology, Daejeon 34141, Republic of Korea.
[10]Personal Genomics Institute (PGI), Genome Research Foundation (GRF), Osong, 28160, Republic of Korea
[†] These authors contributed equally to this work.
[*] Correspondence to: seminlee@unist.ac.kr (S.L.), and jongbhak@genomics.org (Jong B.
Sungwon Jeon [0000-0002-2729-9087]; Hansol Choi [0000-0002-3653-1474]; Yeonsu Jeon [0000-0003-4560-4142]; Whan-Hyuk Choi [0000-0003-1587-5320]; Hyunjoo Choi [0000-0003-2843-0419]; Kyungwhan An [0000-0001-9595-5130]; Hyojung Ryu [0000-0002-2276-850X]; Jihun Bhak [0009-0009-1965-8084]; Hyeonjae Lee [0000-0003-2683-3804]; Yoonsung Kwon [0000-0002-7242-2734]; Sukyeon Ha [0009-0007-8192-3921]; Yeo Jin Kim [0000-0001-9122-914X]; Asta Blazyte [0000-0001-7309-1482]; Changjae Kim [0000-0002-2018-1561]; Yeonkyung Kim [0000-0002-0649-916X]; Younghui Kang [0000-0002-0323-0851]; Yeong Ju Woo [0000-0002-3049-7762]; Chanyoung Lee [0000-0002-8269-3191]; Jeongwoo Seo [0000-0002-9163-4310]; Changhan Yoon [0000-0003-0243-9853]; Dan Bolser [0000-0002-3991-0859]; Orsolya Biro [0000-0002-4300-3602]; Eun-Seok Shin [0000-0002-9169-6968]; Byung Chul Kim [0000-0002-4891-9679]; Seon-Young Kim [0000-0002-1030-7730]; Ji-Hwan Park [0000-0002-6988-6239]; Jongbum Jeon [0000-0003-3533-1363]; Dooyoung Jung [0000-0002-5381-4847]; Semin Lee [0000-0002-9015-6046]; Jong Hwa Bhak [0000-0002-4228-1299].

Abstract

Background

Phenome-wide association studies (PheWASs) have been conducted on Asian populations, including Koreans, but many were based on chip or exome genotyping data. Such studies have limitations regarding whole-genome-wide association analysis, making it crucial to have genome-to-phenome association information with the largest possible whole-genome and matched phenome data to conduct further population-genome studies and develop healthcare services based on population genomics.

Results

Here, we present 4,157 whole-genome sequences (Korea4K) coupled with 107 health check-up parameters as the largest genomic resource of the Korean Genome Project. It encompasses most of the variants with allele frequency > 0.001 in Koreans, indicating that it sufficiently covered most of the common and rare genetic variants with commonly measured phenotypes for Koreans. Korea4K provides 45,537,252 variants, and half of them were not present in Korea1K (1,094 samples). We also identified 1,356 new geno-phenotype associations which were not found by the Korea1K dataset. Phenomics analyses further revealed 24 significant genetic correlations, 14 pleiotropic associations, and 127 causal relationships based on Mendelian randomization among 37 traits. In addition, the Korea4K imputation reference panel, the largest Korean variants reference to date, showed a superior imputation performance to Korea1K across all allele frequency categories.

Conclusions

Collectively, Korea4K provides not only the largest Korean genome data but also corresponding health check-up parameters and novel genome-phenome associations. The large-scale pathological

whole-genome-wide omics data will become a powerful set for genome-phenome level association studies to discover causal markers for the prediction and diagnosis of health conditions in future studies.

Keywords

Korean Genome Project, Genome, Phenome, Population genomics, Variome

Background

South Korea has perhaps one of the most extensive and convenient annual health check-up services. Every year, almost all Koreans aged over 40 receive a standardized health check-up, yielding a wealth of individual clinical data [1]. In 2020, we published 1,094 whole genomes with clinical information (Korea1K) by providing all the participants with a free standard health check-up showing the value of whole-genome data accompanied by clinical information mapping the genome diversity with practical applications [2]. Here, we present the second phase of the Korean Genome Project (KGP) with 4,157 sets of whole-genome data, Korea4K. It is accompanied by 107 types of clinical traits that have been donated by 2,685 healthy participants who acquired the health check-up reports from the hospitals of their choice in the past years. We manually annotated thousands of donated health reports that are matched with the whole-genome information. Therefore, apart from the increased number of samples, the main difference between Korea1K and Korea4K is that Korea4K's clinical information is from very heterogeneous but fairly standard Korean health check-up centers, while Korea1K was from one very well-controlled university hospital health check-up center. This was also a testbed to assess how difficult it would be to merge data from the heterogeneous health check-up record system in a nation for a large-scale genome to phenome association analysis.

Previously, there were a few phenome-wide association studies (PheWASs) on Asian populations, but they were limited to chip or exome-based genotyping data. A Japanese PheWAS identified the genetic links among clinical traits, complex diseases, and cell-type specific patterns [3]. Another PheWAS using 10,000 Korean cohorts' health check-up data from multiple lab sources showed network relationships between genes and phenotypes [4]. However, none of these studies covered the entirety of genomic variation, and they have limitations on genome-wide data analyses [5, 6].

A scientific contribution of this version of KGP is that we provide extensive genome-to-phenome association information with the largest genomic and clinical data from Korea to date to estimate how many samples and clinical parameters cover the whole genomic and common phenotypic diversity of Koreans. Korea4K contains 4,157 Korean genomes from East Asian ancestry, and 2,685 of them are accompanied by 107 types of clinical information such as height, waist circumference, weight, albumin/globulin ratio, basophil, direct bilirubin, low-density lipoprotein, high-density lipoprotein, mean corpuscular volume, and total cholesterol. The rest does not contain such kind of data because the biobank does not have phenotype information, or we were not able to collect it from the participants. Korea4K extends the efforts to completely map the totality of Korean genomic diversity, which can be a useful scope reference for disease risk prediction, diagnosis, and treatments in the future for personalized medicine.

As the second phase of the KGP, Korea4K not only extends the previously reported Korea1K [2] but also includes new multi-phenotypic association analyses, that is, analyses on markers that are associated with multiple phenotypes (pleiotropy), the genetic correlation between traits, and estimated causality relationship among traits through Mendelian randomization (MR) and 3D structure models for Korean specific missense variants. Combining these two omics data, we provide the community with the most extensive geno-phenotype association of healthy Korean participants. We have also applied the genomic variation data to the genotype imputation of low-frequency variants in the Korean population.

Data Description

The goal of our project was to create a genome dataset for Korea4K, which included newly sequenced genomic data from 2,848 participants as well as 1,309 whole-genome sequencing (WGS) datasets from Korea1K and public data archives. Additionally, we established a phenome dataset for Korea4K by gathering or computing 107 clinical parameters and genome data from 2,685 samples. We collected a total of 3,383 clinical datasets, including multiple time points per sample, from regular health checkups conducted by various hospitals and clinics across Korea between 2016 and 2019. The genome and phenome datasets were produced and curated by the protocol in Material and Methods.

Analyses

The largest Korean whole-genome variants data: Korea4K variome

A total of 64,301,272 single nucleotide variants (SNVs) and 8,776,608 Indels were called against the human genome reference (hg38) from the 4,157 Korean whole genomes, including 3,071 healthy controls (Supplementary Table S1 and S2). It contains 3,063 newly added whole genomes sequenced by Illumina next-generation sequencing (NGS) platforms (HiSeq X10 and Novaseq 6000), in addition to the previous Korea1K dataset which was mostly generated by Illumina HiSeq X10. Using the variant data, we selected 3,617 samples with no kinship after initial sample filtering (see Methods). To exclude erroneous variants from sequencing batch effects from the heterogeneous Illumina NGS platforms and library preparation, we applied an allele balance bias measurement and finally acquired 12,713,580 erroneously called variant candidates (Supplementary Fig. S1). After additional variant filtering (see Methods), we identified 45,537,252 variants including 42,124,137 SNVs, 36,029 double nucleotide variants (DNVs), 26,135 triple

nucleotide variants (TNVs), 3,261,682 indels, and 89,269 other types of small variants from the 3,617 unrelated samples. We named this filtered Korean dataset the Korea4K variome (Figure 1). A total of 23,689,147 variants were not present in the previous Korea1K variome. This unexpectedly large difference is likely derived from different batch effect filtering, and variant calling and filtering procedures, as well as new variants from the larger sample size. Consistent with the Korea1K study [2], most variants were located in intronic or intergenic regions and rarely in splicing sites or coding regions (Supplementary Fig. S2), which is a sign of negative selection pressure in the population. Half of the total variants (21,941,879; 48.2%) were singleton or doubleton in the 3,617 unrelated samples, indicating that the Korean population's genetic diversity is very low as the population diversity could be covered by fewer than 4,000 unrelated samples (Figure 1A, Supplementary Table S3). Almost all the common (allele frequency of > 0.01 and allele frequency of ≤ 0.05) and very common (allele frequency of > 0.05) variants were found to be already reported in the dbSNP database (99.70% and 99.97%, respectively), while more than half of the singleton and doubleton variants were newly discovered in this study (59.9% and 44.57%, respectively), indicating the new variant pool is well-exhausted in the Korean population by the 3,617 samples resulting in a large portion of individual specific novel variants in the Korean variome (Figure 1A, Supplementary Table S3). Only 3,092 and 3,569 unrelated individuals were needed to discover all the rare (allele frequency of > 0.001 and allele frequency of ≤ 0.01) and very rare (allele count of > 2 and allele frequency of ≤ 0.001) variants in the Korea4K variome, respectively (Figure 1B) indicating that the Korea4K variome includes almost all the rare and very rare variants of Korean people of East Asian ancestry. It is notable that in our previous Korea1K data, the accumulated variant number curves did not reach a plateau [2]. Regarding common variants, only 481 and 161 unrelated individuals were necessary for common and very common

variants, respectively, to cover the diversity which is close to the Korea1K statistics (440 and 132 samples). Essentially, the Korea4K variome statistics indicate the saturation of population diversity detection among Koreans. However, as expected, in the case of singleton and doubleton variants, the Korea4K variant discovery curve did not reach a plateau. This is due to each individual’s novel random variants and we will never reach a point of no novel variant discovery even with increased sample numbers.

As a practical application, we constructed a Korea4K imputation reference panel from the 3,614 unrelated whole-genomes that showed a consistently better imputation performance than the Korea1K. The Korea4K panel was able to impute 198,805 more genotypes than the Korea1K panel (7,551,095 loci compared to 7,352,290) with the same dataset. Moreover, as expected, the Korea4K panel had better accuracy across all allele frequency categories than the Korea1K panel (Figure 1C). The difference in aggregated R^2 became larger for variants with allele frequency (AF) in Korea4K < 0.05 than for those in Korea1K, indicating higher accuracy in rare variants (Figure 1C). In particular, the Korea4K imputation panel improved the imputation accuracy by 6% for the rare variants group compared to Korea1K on average.

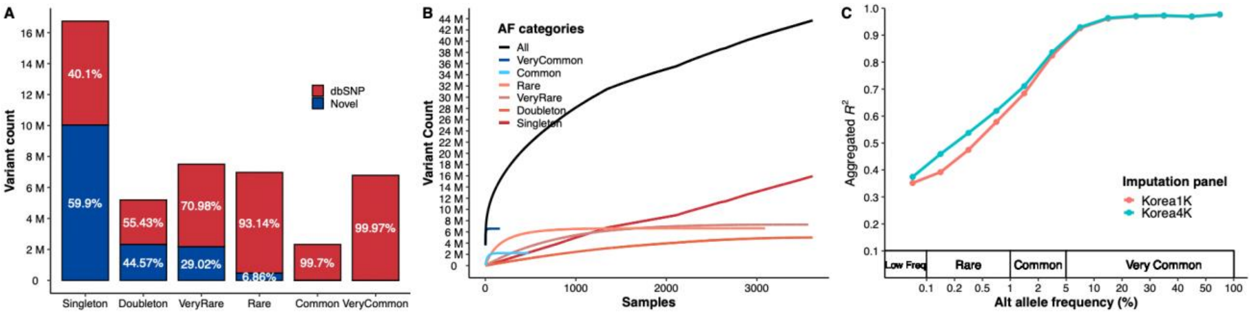

Figure 1 Korean variome profile and imputation evaluation using Korea4K (A) The number of variants in the Korea4K variome is categorized by allele frequencies (AFs) among unrelated Korea4K genomes. “dbSNP” indicates the variants were reported in dbSNP database. “Novel”

indicates the variants were not reported in dbSNP. Singleton, allele count = 1; doubleton, allele count = 2; very rare, allele count of > 2 and allele frequency of ≤ 0.001; rare, allele frequency of > 0.001 and allele frequency of ≤ 0.01; common, allele frequency of > 0.01 and allele frequency of ≤ 0.05; very common, allele frequency of > 0.05. **(B)** The number of discovered variants as a function of unrelated genomes. **(C)** Imputation performance evaluation using the Korea4K and Korea1K panels. The X-axis indicates alternative (Alt) allele frequency in the Korea4K variome. The Y-axis represents the aggregated R^2 values of variants. We used variants that were overlapped by imputed results across two panels.

As in Korea1K, the Korean population is genetically distinct from the Chinese and Japanese populations, confirmed by principal component analysis (PCA) with few outliers (Figure 2A). We also found 62 missense variants out of 282,607 in Korea4K that had AFs significantly different from ten populations in the 1000 genome project (1KGP) from European Bioinformatics Institute (EBI), Cambridge, UK (Chi-squared test $P < 5 \times 10^{-5}$ against each of the ten populations, see Methods; Supplementary Table S4). The genes containing such Korean-specific missense variants included *LILRB3*, *HLA-DRB5*, *IGLV5-48*, and *IGHV4-4* that are known to be associated with adaptive immunity, and *OR9G1* and *OR8U1* for olfactory receptors. Additionally, we found that twelve Korean-specific missense variants were in protein functional domains (Figure 2B). Four of them were predicted to facilitate increased structural stability calculated in the protein 3D models built by AlphaFlod2 [7], while the other eight variants were predicted to cause decreased stability (Supplementary Table S5).

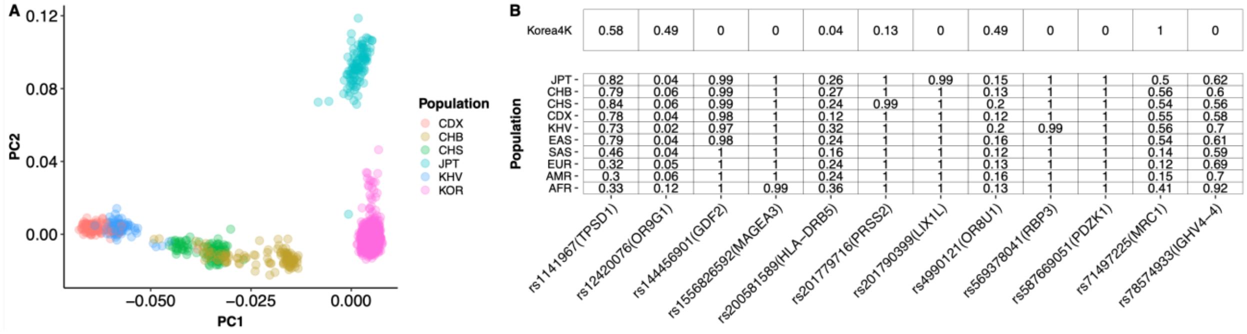

Figure 2 Comparison of Korea4K and 1KGP (A) The results from principal component analysis of Korea4K and the 1KGP set of East Asian samples. **(B)** Allele frequency information of Korea4K and the populations in the 1KGP for the twelve Korean-specific missense variants located in protein functional domains. KOR: Korea4K; CDX: Dai Chinese; CHB: Han Chinese; CHS: Southern Han Chinese; JPT: Japanese; KHV: Kinh Vietnamese; EAS: East Asians; SAS: South Asians; EUR: European; AMR: American; AFR: African.

Whole-genome-wide association study (WGWAS)

Whole-genome-wide association studies (WGWASs) revealed that 2,324 variants from 157 unique loci had significant associations with 34 clinical traits from 37 WGWAS target traits ($P < 5 \times 10^{-8}$; Figure 3A-F, Supplementary Table S6). Among the significantly associated variants, 2,314 variants from 30 clinical traits still showed significance after false discovery rate (FDR) correction using the Benjamini-Hochberg approach (FDR < 0.05). We used 90 clinical traits from the 107 phenotypes after filtering 27 traits with a high missing rate and biased distribution for WGWASs (see Methods). Of the 90 traits, 54 were not confident in Quantile-Quantile plots and were excluded from further Mendelian randomization and pleiotropy analyses (see Methods). Among the 2,324

WGWAS significant variants, only 85 variants (31 loci) were reported in the GWAS catalog database [8]. The trait with the largest number of significantly associated loci was carbohydrate antigen 19-9 (CA19-9), a cancer antigen, with sixteen loci. Uric acid had the second highest number of significant loci with fourteen loci.

Korea4K showed much stronger statistical power than the previous Korea1K study, identifying 1,356 new WGWAS variants (107 loci) from 28 common traits between Korea4K and Korea1K. Also, Korea4K had much lower (i.e., more significant) *P*-values than Korea1K for all the commonly found association variants between the two datasets (Supplementary Fig. S3). Among the 107 loci containing the 1,356 new WGWAS variants, 798 Korea4K significant WGWAS variants from 73 loci had not been significant in Korea1K (Supplementary Table S6). Furthermore, twelve traits (albumin/globulin ratio, basophil, C-reactive protein, direct bilirubin, height, low-density lipoprotein, mean corpuscular volume, right hearing at 2000hz, thyroid stimulating hormone, total cholesterol, waist, weight) had 425 WGWAS variants that were significant uniquely in Korea4K, meaning no significant WGWAS variants from the twelve traits in Korea1K (Supplementary Table S6). For example, a missense variant, rs6431625 ($P = 1.41 \times 10^{-23}$, FDR = 5.23×10^{-18}), in *UGT1A3* was found to be associated with direct bilirubin in Korea4K. It was previously reported to be associated with circulating bilirubin levels [9]. Another Korea4K-specific missense variant is rs7412 ($P = 2.86 \times 10^{-14}$, FDR = 1.11×10^{-7}) in *APOE* which is associated with low-density lipoprotein (LDL) levels. Its association with cholesterol levels has been previously well-established [10]. Finding novel WGWAS variants in Korea4K was due to the increased sample size and subsequently increased variant number compared to Korea1K.

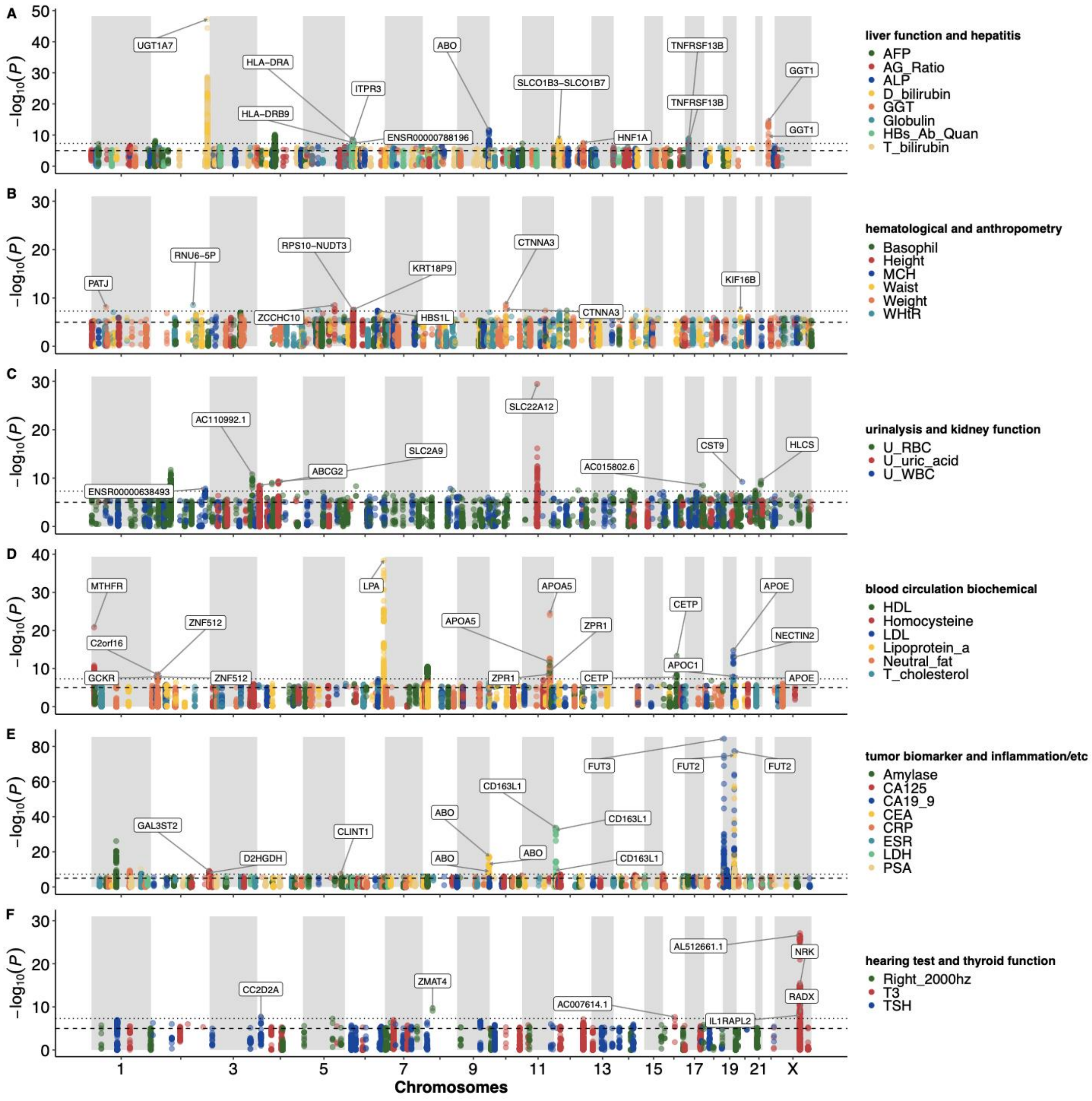

Figure 3 Whole-genome-wide association studies in Korea4K. (A-F) Whole-genome-wide association studies from 34 traits. Loci are presented only when index variants of the loci had significant *P*-value ($P < 5 \times 10^{-8}$) from the WGWAS. The dashed line indicates the suggestive threshold ($P < 10^{-5}$). The dotted line indicates the significant threshold ($P < 5 \times 10^{-8}$).

Genetic correlation (GC) and phenotypic correlation (PC)

We found 27 traits with significant heritability among 89 quantitative traits (Figure 4A; the lower boundary of genetic heritability > 0 with 95% confidence interval; Supplementary Table S7). A total of 24 pairs of traits showed a significant genetic correlation ($FDR_{GC} < 0.05$), measured as rG value, among 351 trait pairs between the 27 traits that showed significant heritability (Figure 4, Supplementary Table S8). We found consistent results of Weight-Waist and body mass index (BMI)-Waist pairs, showing a significant genetic correlation in the UK Biobank data with the same trend as our result (rG = 0.9, $P = 10^{-308}$ in UK Biobank; rG = 0.9, $P = 10^{-308}$ in UK Biobank, respectively) [11]. We identified 2,274 trait-trait relationships that had significant phenotypic correlation ($FDR_{PC} < 0.05$, its 95% CI does not include 0) from trait-trait associations between 3,916 pairs of 89 quantitative traits (Figure 4B, Supplementary Table S9). Most genetic and phenotypic correlations showed the same direction of correlation. The only two exceptions were waist/weight ratio (WWtR) – Urine white blood cell (U_WBC) and Waist-Creatinine which showed opposite directions. This trend of Waist-Creatinine has also been reported in a correlation database using UK-biobank data [12]. Such discrepancies between the correlation estimates are possibly derived from the shared environmental factors between a pair of traits, such as dietary habits, that overwhelm the genotypic effects [13, 14]. This proves that the phenotypic correlation is not a mere proxy for the genetic correlation and consideration on the environmental effect is indispensable for the accurate interpretation of human phenomics [15].

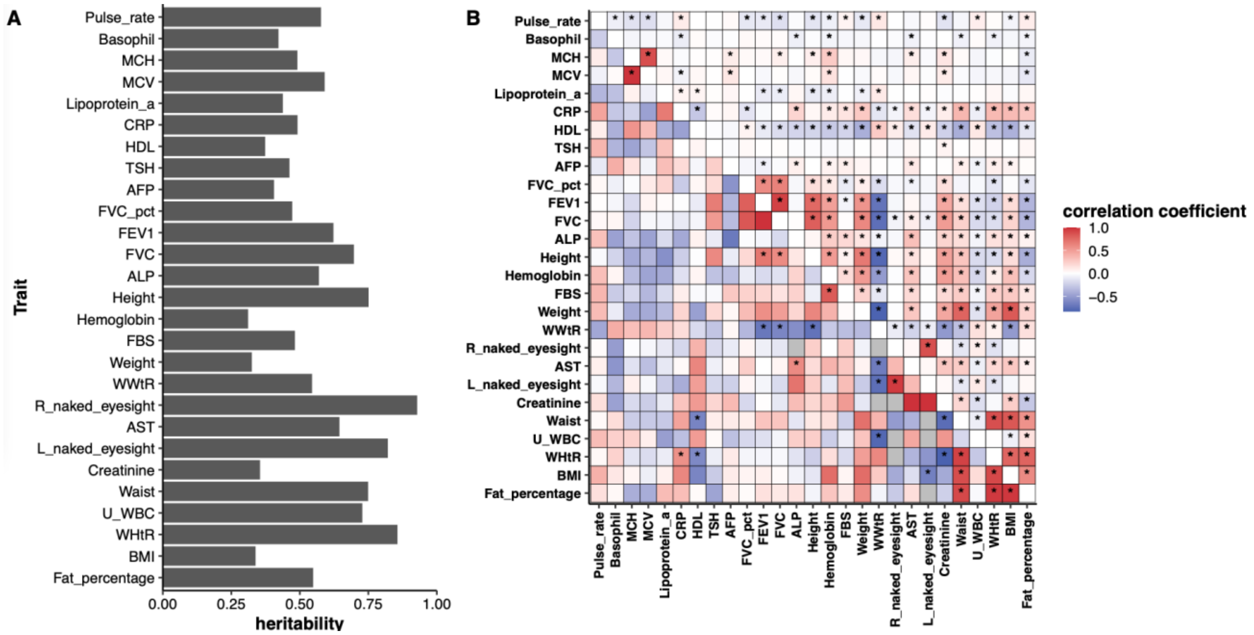

Figure 4 Genetic correlation and Phenotypic correlation in Korea4K. (A) Genetic heritability of 27 traits that showed at least a marginal significance. **(B)** Genetic correlation and phenotypic correlation between the 27 traits. The upper triangle indicates phenotypic correlation coefficient (Pearson's) and lower triangle indicates genetic correlation coefficient (rG).

Pleiotropy and Mendelian randomization (MR)

Out of the 37 WGWAS target traits, we detected 1,131 variants from 21 traits having suggestive associations ($P_{GWAS} < 10^{-5}$) with at least two traits, indicating pleiotropic variants (Figure 5, red edges; Supplementary Table S10). We devised the Variant-Sharing Index (VSI) to measure the degree of intersection between two phenotypes (Table 1; see Methods). A VSI of zero signifies that two traits share no suggestively associated variants (SSVs), while 100 indicates they share all of them. The trait pairs with SSVs and the corresponding VSIs are listed in Table1. Notably, we had only one variant, rs77913154, that was shared among three traits: Globulin, AG_Ratio, and ESR (Supplementary Table S10). Interestingly, we found fifteen variants residing on *SOD2P1*-

AC095032.2-AC095032.1 locus forming pleiotropy between the serum amylase level and the level of CA125, a known ovarian cancer marker (Table 1, VSI=2.3). Fourteen variants of the fifteen variants conform to the alteration of *AMY2B* expression level, as per cis-eQTL results from the GTEx Portal (ver. 8), four of which were associated with expression in the pancreatic tissue (see Methods). There have already been reports of hyperamylasemia in patients with ovarian cancer [16-18]. In addition to the investigation on the general pleiotropic relationship, we employed Mendelian Randomization (MR) to detect vertical pleiotropy that can assert the direction of the phenotypic relationships [19]. This provides indirect evidence implying causality between the traits to discern spurious phenotypic associations, such as confounding and collider bias [20, 21]. We found a total of 127 trait pairs among 1,332 pairs of the 37 WGWAS traits were estimated to have significant causal relationships (FDR < 0.05, Figure 5, Supplementary Table S11). These findings were supported by at least two of three different Mendelian randomization (MR) analysis methods (IVW: 166 pairs; MRPRESSO: 139; MR-Egger: 23). Among these, 59 trait pairs showed unidirectional relationships while 68 exhibited bidirectional causal relationships (Supplementary Table S11).

Table 1 Pleiotropic associations and Variant-Sharing Index (VSI)

Trait1	Trait2	Suggestive variants in trait1	Suggestive variants in trait2	Shared variants	Total variants	VSI
D_bilirubin	T_bilirubin	638	632	569	701	81.2
Globulin	AG_Ratio	294	230	147	377	39
HDL	Neutral_fat	348	398	191	555	34.4
CEA	CA19_9	221	264	74	411	18
T_cholesterol	LDL	74	238	38	274	13.9
WHtR	Waist	177	100	31	246	12.6
ALP	CEA	153	221	35	339	10.3
T3	GGT	542	125	23	644	3.6
CA125	Amylase	202	466	15	653	2.3
Weight	Waist	123	100	5	218	2.3
Height	Weight	173	123	2	294	0.7
ESR	AG_Ratio	163	230	1	392	0.3
Globulin	ESR	294	163	1	456	0.2
U_RBC	Globulin	627	294	1	920	0.1

Summary results of the four phenomics analyses

We summarized the results of four phenomics analyses (Genetic correlation, Phenotypic correlation, Mendelian randomization, and pleiotropy) through visualizing them in network plots (Figure5). In general, the identified trait-trait pairs of genetic correlation, Mendelian randomization, and pleiotropy analyses did not often overlap. Genetic correlation and pleiotropy are found to be exclusive of each other, even though both measures having shared genetic components of two different traits. GC was primarily observed among body measures such as waist circumference, weight, height, and left naked eyesight. On the other hand, pleiotropy was more prevalent on the relationship between metabolites in blood such as LDL, bilirubin, or CEA. The only overlap between these two was WHtR (Waist-to-Height Ratio)-Waist (circumference), where one is derived from the other.

MR suggests a causal relationship between phenotypic correlations through meditation effect by a genotype. In our casual diagram (Figure 5, blue arrows), ALP and CEA showed potential causality, along with the shared genetic variants between them (pleiotropy near *ABO* gene). Numerous previous studies have consistently reported these markers together for diagnosing cancer and monitoring metastasis [22-24]. Similarly, CA125 and Amylase also displayed causality via shared genetic variants (pleiotropy near *AMY2B* gene). We propose that CA125 and Amylase might serve as complementary biomarkers for ovarian cancer, much like ALP and CEA. The biological relationships between these clinical blood measures remain unclear.

Our phenomics results also depicted distinguishable patterns of association between secondary body measures, such as WHtR (Waist-to-Height Ratio), WWtR (Waist-to-Weight Ratio), and BMI (Body Mass Index), with other phenotypes. WHtR exhibited a causal relationship with CRP (C-reactive protein), body fat percentage, and HDL. The result is concordant with previous reports that body fat percentage and CRP are correlated [25, 26]. Conversely, WWtR had casual associations with measures of lung capacity (FEV1 and FVC), liver function (AST), and inflammation (U_WBC). However, WWtR has yet to be proven its utility in clinical studies. BMI serves as an intermediate phenotype, sharing most of its associations with WHtR and, to a lesser extent, with WWtR via left naked eyesight. These findings suggest that the measurements reflect distinct biological mechanisms, warranting further studies. For instance, WHtR is a well-known indicator of central adiposity which provides a better estimate of obesity and related morbidities than BMI [27].

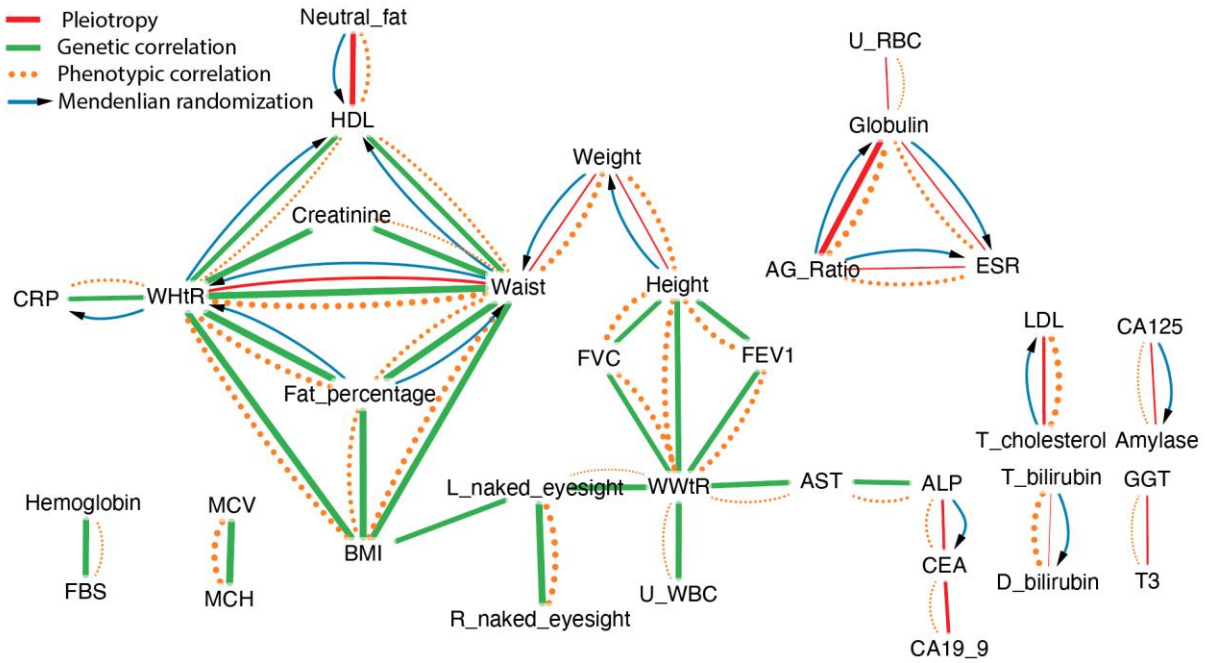

Figure 5 Graph visualization of genetic correlation, phenotypic correlation, pleiotropy, and Mendelian randomization. Green line indicates significant genetic correlation (GC), and the edge thickness indicates the absolute value of the correlation coefficient. Red line indicates trait pairs that have pleiotropic variants. Dotted orange lines indicate phenotypic correlation (PC), and the edge thickness indicates the absolute value of Pearson's correlation coefficient. Blue arrow line indicates a causal relationship from Mendelian randomization (MR). MR and PC were visualized only when at least one of GC or Pleiotropy relationships was observed between the traits.

Discussion

Batch effect exacerbated by sequencing platform and library preparation bias is a critical problem in very large population genome association studies, especially with clinical data from heterogeneous health check-up centers. In the future, more and more diverse whole-genome data with extensive clinical data will be publicly available, and it is inevitable that they will be merged

for more precise whole genome-to-phenome association research. Korea4K is not an exception in that regard, and in one homogeneous population WGWAS, it was necessary to consider and factor in a great deal of sequencing and clinical data batch effects and errors. We attempted to minimize the errors by using allele balance with optimal filtering criteria and time-consuming manual checks on health reports that were donated by the participants (see Methods). The largest challenge of Korea4K project was cleaning up heterogeneous clinical data from different health check-up centers. Another major issue was that the health check-up data heterogeneity caused reduced numbers of participants' common traits with which to compare. Some of the health data were from past years' health check-ups from heterogeneous hospitals throughout South Korea. This heterogeneity in location and time was not an intentional experimental design but was in order to reduce the cost of performing expensive one-center health check-ups for the Korea4K participants. Therefore, WGWAS along with standardized and unified national and public health check-up data will greatly benefit future whole-genome-wide association studies.

Although 4,157 seems like a large number, we found the sample size in this study was still not large enough to detect weak association signals. The Korea4K variome with matched phenotype information has allowed us to estimate genomic correlation across various phenotypes using GREML [28]. GREML has been reported to have higher accuracy compared to methods, such as linkage disequilibrium score regression (LDSC), using only summary statistics from GWAS [29]. For example, the minimum heritability score was 0.34 (Degree of obesity) among the traits detected as statistically significant. The statistical power of our maximum 2,685 subjects and FDR < 0.05 is estimated to be 0.72 for detecting traits with heritability of 0.3 or higher (Calculated from GCTA-GREML Power Calculator) [30]. This will increase to 0.97 with 4,000 subjects. In other

words, phenomics analyses were limited and not powerful enough to confidently discover novel phenotypic associations with the current dataset.

Nevertheless, our findings bear important practical implications. We described the utility of secondary body measures, such as WHtR and WWtR, compared to BMI. We also elaborated on the diagnostic and prognostic value of other serum proteins, namely ALP and Amylase, in conjunction with the existing cancer biomarkers. However, we plan to collect more samples for sequencing and health record data with a wider variety of health-related categories to conduct a more powerful study in the future. This will allow us to not only validate our findings but also find correlations of medical importance that were missed in the present study. While chip-based GWAS is a common approach, our study highlights the unique advantage of WGWAS (whole-genome-wide association) in genotype-phenotype association studies. An illustrative advantage of WGWAS is its whole-genome-wide, unbiased coverage of genetic variants, which allowed us to assign specific variants accounting for pleiotropy. This was not achievable with conventional methods. For example, we could identify the variants in the well-known pleiotropic relationships such as ALP-CEA by *ABO* locus (35 variants), Neutral_Fat-HDL by *LPL* locus (181 variants) and Total cholesterol-LDL by *TOMM40*, and *APOE* locus (4 and 2 variants, respectively) (Supplementary Table S7). These loci and their corresponding trait pairs were previously reported from chip-based GWAS summary results [31, 32]. Similarly, we anticipate the fine-mapping analyses will also benefit from WGWAS, pinpointing novel genetic variants of phenotypic importance, as demonstrated in our prior work [33]. Taken together, whole-genome sequencing with its genomic completeness should be a well-considered choice for future genomic association studies.

One of the main objectives of the Korea4K project was to build a genomic and phenomic reference dataset to discover unknown whole-genome to phenome associations that can be detected from samples of healthy people. This, however, is contradictory and it limited us in discovering clear pathogenic associations because most of the participants examined in WGWAS were healthy without any severe aberrant phenotypes or diseases that could bring us clues for interesting omics analyses. Moreover, utilizing recently introduced human genome references like the T2T reference [34] and Human Pangenome reference [35], which offer broader genomic coverage or have population-specific sequences compared to the existing GRCh38 reference, could help identify additional associations that might be overlooked. Nevertheless, these new references lack functional annotations and need to be connected to previous databases such as dbSNP and the GWAS catalog.

As for the future directions, there are several key limitations that have not been met in our current study. The first is we failed to acquire long DNA sequencing reads from the healthy participants for building a structural variation reference set for the Korean population. The second is the lack of epigenomic data from the 4,157 samples. This was mostly due to high costs for generation and computing long-read based assemblies and sequencing methylated DNA sites. The third one, which is perhaps the most relevant for the purpose of performing association studies for healthcare is that we failed to acquire more rare and severe disease data from patients, accompanied by precise clinical and multiomics data. We have excluded a small number of rare disease cases, as those required a large amount of sequencing data from genome, transcriptome, and methylome to perform precise functional analyses. Large-scale pathological whole-genome-wide omics data will become a powerful set for genome-phenome level association studies to detect causal markers for the prediction and diagnosis of health conditions in future studies.

Potential Implications

The Korea4K dataset can be a valuable variome reference, as it contains matched phenome data for personalized medicine, large-scale population genome studies, and the understanding of anthropologic history in Korea. This large-scale Korean genome-phenome dataset can help identify genetic basis for diseases and phenotypes, enabling personalized treatment plans for individuals. Analyzing the genome-phenome association dataset can also be used to develop new drugs that target specific genetic variations in the Korean population. The Korea4K dataset can also be valuable for other populations, particularly East Asians, as it can be used to identify population-specific genome-phenome patterns by comparing the population's genome-phenome data to the Korea4K dataset. Furthermore, the Korea4K reference panel can be utilized for genotype imputation of DNA chip genotyping data for the Korean population and other East Asians.

Materials and Methods

Sample collection and whole-genome sequencing

We collected 2,848 blood samples or already processed DNA samples from Korean individuals. A total of 1,094 whole-genome sequencing (WGS) datasets originating from our previous study (Korea1K) and 215 WGS data from publicly available Clinical & Omics Data Archive (CODA) were added to the aforementioned dataset [2]. The genomic DNA was extracted using the DNeasy Blood & Tissue kit (Qiagen) from whole blood samples. We constructed the whole-genome sequencing library from the DNA by using the TruSeq Nano DNA Sample Prep kit (Illumina) kit. Whole-genome sequences of the 2,848 samples were generated by the Illumina Nova-seq 6000

platform. All the sequencing data that we used in this study had 151bp as a read length. Average sequencing amount per sample was 27.75× (Supplementary Fig. S4).

Joint genotype calling

Adapter contamination was trimmed using Cutadapt (RRID:SCR_011841, ver. 1.9.1) [36] with a forward adapter (′GATCGGAAGAGCACACGTCTGAACTCCAGTCAC′) and reverse adapter (′GATCGGAAGAGCGTCGTGTAGGGAAAGAGTGT′) and with a minimum read length of 50 bp after trimming (Supplementary Fig. S4). The quality of trimmed reads were screened by FASTQC program (RRID:SCR_014583, ver. 0.11.5) We mapped the whole-genome sequencing reads from 4,157 samples to the human reference genome (hg38) using BWA-mem (RRID:SCR_010910, ver. 0.7.17) with the '-M' option and alt-aware mode [37]. The mapped reads were sorted by genomic coordination using Picard (RRID:SCR_006525, ver. 2.20.3). We marked the PCR-duplicates and recalibrated the base quality of the mapped reads using the MarkDuplicates and BaseRecalibrator module in Picard (RRID:SCR_006525, ver. 2.20.3), respectively. A total of 3,156 samples had a mapping depth of ≥ 20× (Supplementary Fig. S4). Individual genotypes were called in GVCF format by HaplotypeCaller in GATK (RRID:SCR_001876, ver. 4.1.3) with '--genotyping-mode DISCOVERY -stand-call-conf 30 -ERC GVCF' options [38]. We merged the individual genotypes to a single GVCF for each chromosome using CombineGVCFs in GATK (RRID:SCR_001876, ver. 4.1.3) [38]. We jointly genotyped the merged GVCF with the genotypeGVCF module in GATK (RRID:SCR_001876, ver.4.1.3) [38]. Variant quality of the joint genotypes was recalibrated using the VQSR module in GATK (RRID:SCR_001876, ver. 4.1.3) [38].

Sample and variant filtering

After joint genotyping, we filtered out a total of 540 participants on the criteria that are listed below using SelectVariants in GATK (RRID:SCR_001876, ver. 4.1.3) with '--remove-unused-alternates' option to remove unused variants [38]. To explore kinship relations among the samples, we assessed Identical by Descent (IBD) using the Plink program (RRID:SCR_001757, ver. 1.90b3n) [39]. Samples with a PI_HAT value exceeding 0.05 were considered to be in a kinship relation.

1. showing high missing genotype rate (>10%): nine samples
2. having too high or low heterozygous variants ratio compared to homozygous variants per sample (3 s.d.): four samples
3. having relatedness to other samples: 428 samples
4. having non-Korean genetic background from PCA analysis with 1KGP set: seven samples
5. reported to have a rare disease: 40 samples
6. 52 samples who became not applicable for this study

Finally, the Korea4K variome data included 3,617 participants' genomes. To detect variants which were probably called because of a sequencing batch effect, we measured average allele balance of the genotyped alleles (the read count of the allele divided by the total read count on a locus). Then, we excluded 12,713,580 variants that had average allele balance of the loci out of the range of ± 1 × standard deviation (SD) from a genome-wide average of allele balance to remove the sequencing batch effect (Supplementary Fig.S1). We also excluded the variants which had a genotyping rate of < 0.9 for downstream variant analysis. The variants in the final variome set were annotated using Variant Effect Predictor (VEP) with Ensemble database (RRID:SCR_007931, ver. 101) [40].

Principal Component Analysis (PCA) with the EBI's 1KGP genome data

The interpopulation genomic structure was evaluated by projecting the first two PCs determined via PCA of SNVs from both Korea4K and East Asian populations from 1KGP. We merged variants from the Korea4K and 1KGP sets and then filtered out variants with the following criteria: (i) biallelic SNVs with a MAF < 1%; (ii) biallelic SNVs with an HWE $P < 10^{-6}$; (iii) biallelic SNVs with a missing genotype rate of > 0.01. Extracted variants were LD pruned using " --indep 200 4 0.1" option in PLINK (RRID:SCR_001757, ver. 1.90b3n) [39], yielding 330,350 sites. PCA was carried out using PLINK (RRID:SCR_001757, ver. 1.90b3n) [39].

Korean-specific missense variants

We collected allele frequency data from ten populations (African (AFR), American (AMR), European (EUR), South Asian (SAS), East Asian (EAS), Japanese in Tokyo (JPT), Kinh Vietnamese (KHV), Han Chinese in Beijing (CHB), Han Chinese Southern (CHS), and Chinese Dai in Xishuangbanna (CDX)) from EBI's 1KGP database [41]. For each Korea4K variant, we compared its allele frequency to the allele frequency of all of the ten populations using the Chi-squared test. We selected variants that were specific to the Korean when the *P*-value of the Chi-squared test to the ten populations was less than 5×10^{-5}.

Protein structure modeling and thermodynamic stability measurement

We constructed the mutant-type (MT) protein sequences of the Korean-specific missense variants by substituting the reference protein sequences found in the Ensembl database (RRID:SCR_002344, ver. 101) [42]. We modeled the structures of the wild-type (WT) and mutant-type protein models using AlphaFold2 (ver. 2.0) with the '--max_template_data 2022-05-09 --

db_preset reduced_dbs' option with default databases downloaded by AlphaFold2 [7]. We used the InterPro (RRID:SCR_006695) database [43] to determine whether a missense variant was located in the domain region within the protein sequence. We extracted the domain region from the WT and MT protein 3D models and excluded domains that had less than 50 amino acids. Afterwards, we calculated ΔG_{WT} and ΔG_{MT} using the 'Stability' command of foldX (RRID:SCR_008522) [44] to measure the protein thermodynamic stability. Finally, we measured the change in protein thermodynamic stability between the two models by calculating the difference between the WT and MT domain models ($\Delta\Delta G = \Delta G_{MT} - \Delta G_{WT}$).

Imputation

We constructed an imputation reference panel of Korea4K and Korea1K sets which includes 3,614, and 873 Korean individuals, respectively. A total of 26,210,741 and 15,649,303 autosomal biallelic variants with a missing genotype call rate of < 0.1 and minor allele count > 1 (not a singleton) were extracted for the Korea4K and Korea1K panel, respectively. The extracted variomes were phased into haplotype using SHAPEIT2 (ver. v2.r904) [45]. We used the same test dataset as in the previous study [2]. The phased test data was imputed using the imputation reference panel by Minimac3 (RRID:SCR_009292, ver. 2.0.1) [46]. We estimated imputation accuracies using squared Pearson's correlation coefficients (R^2) between the true genotypes and imputed genotype dosages.

Clinical information

We collected or calculated 107 clinical parameters (93 quantitative and 14 qualitative traits; Supplementary Table S12) along with genome data from 2,685 samples among the Korea4K

samples. A total of 3,383 clinical datasets (including multiple time points per sample) from regular health checkups carried out by various hospitals and clinics throughout Korea were collected from 2,685 participants between 2016 and 2019. When a single participant had multiple clinical datasets, the most recent one was chosen for the following analysis. Out of the final unrelated 3,617 samples, 2,374 samples had clinical data available and were included in the phenomics analyses.

In the context of collecting data from over 200 diverse healthcare institutions, standardizing clinical information on 107 traits became imperative. We resolved discrepancies in unit measurements, such as micrograms and nanograms, for specific traits. Furthermore, certain clinical metrics, such as the estimated glomerular filtration rate (e-GFR), were found to exhibit variability contingent upon variables such as ethnicity, sex, and age. To maintain consistency and ensure methodological uniformity, we enforced the adoption of a singular clinical formula for the computation of e-GFR across all data samples. Such calculations were applied to 26 traits which are shown in Supplementary Table S13. Clinical traits that exhibited values characterized by inequalities likely due to the limit of detection (e.g., < 5.0 and > 99) were omitted from the analytical procedures, as such values have the potential to introduce disturbances to subsequent data analyses. Likewise, values that exhibited divergent formatting conventions across distinct healthcare institutions (e.g., 20 and a few or 999 and many) were harmonized to conform with prevailing standard criteria observed in most samples under investigation. Also, four quantitative clinical traits and 12 qualitative traits were excluded from the further analysis, since the traits were missing from more than 90% of participants due to health check-up reports heterogeneity, or the traits that were qualitative and biased to one category (more than 1:4). Standard Weight was also removed from the analysis, because the trait was not an inherently correct representation of the sample's clinical data but rather a recommended value. Three traits (Hepatitis B virus antibody,

antigen, and hepatitis C antibody) contained both quantitative and qualitative values. Therefore, both of the values were utilized for analysis, i.e, Hbs_Ab_Quan and Hbs_Ab_Binary. Phenotypic correlations were calculated by Pearson's method. Benjamini-Hochberg method was used to adjust for multiple comparisons when documenting confident phenotypic correlations with FDR.

Whole genome-wide association study (WGWAS)

SNVs and indels with a MAF <1%, HWE $P < 10^{-6}$, and a missing genotype rate of > 0.01 were excluded from the analysis using PLINK (ver. 1.90b3n) [39]. A total of 90 WGWAS (88 quantitative and 2 qualitative traits) were performed with a total of 3,617 individuals and 7,782,381 variants. Each WGWAS had a different number of individuals that included those who had the target clinical traits. The WGWAS was performed using linear and logistic regression under an additive genetic model with PLINK (ver. 2.00 alpha) [47] for quantitative and qualitative traits, respectively. Sex, age, age^2 (age squared), body mass index (BMI), and the top ten principal components of SNV genotypes were included in the model as covariates. Age and BMI were chosen especially due to their known shared associations with multiple traits as previously documented by Shungin and colleagues which could lead to confounding biases in the downstream interpretation of phenotypic relationships [48]. BMI was excluded from covariates in the WGWAS for BMI itself and degree of obesity. Calculating the genomic inflation factor (λ_{Median}), we found that all of the traits in the test reside below 1.1 indicating there are minimal false positives caused by gross population structure or systematic biases (Supplementary Fig. S4-19) [49]. We rejected 53 traits from further analysis based on QQ-plot analysis (Supplementary Fig. S5-S20). We used 5×10^{-8} for a whole-genome-wide significance threshold. The 7,782,381 variants were clumped into 466,938 loci based on linkage disequilibrium (LD) information using PLINK (ver. 1.90b3n)

with '--clump-p1 1, --clump-p2 1, --clump-r2 0.1, --clump-kb 250, and --clump-index-first' options [39]. Statistical powers of the 90 WGWAS were calculated by the R package "genpwr" under the assumption of an effect size of 0.5 and a minor allele frequency of 0.01 (Supplementary Table S14).

Measuring heritability and genetic correlation

We calculated genetic relatedness among individuals from SNPs by genetic relationship matrix (GRM) in genome-wide complex trait analysis (GCTA) (ver. 1.93.2) with ' –autosome –maf 0.01 –make-grm' options [28]. We estimated the genetic heritability of 87 quantitative traits using GCTA (ver. 1.93.2) with '–reml –grm' options [28]. We estimated the genetic correlations (GC) using the bivariate genome-based restricted maximum likelihood (GREML) algorithm [50] in the GCTA (ver. 1.93.2) with '–reml-bivar –grm –reml-bivar-lrt-rg' options [28]. Two of the 253 trait pairs were excluded since the log-likelihood did not converge. The correction for multiple tests was done by Benjamini-Hochberg approach when reporting confident GCs that suffice the threshold of FDR below 0.05.

Calculation of Variant Sharing Index (VSI)

The variant sharing index (VSI) is a Jaccard score to measure how many pleiotropic components exist out of all significant variants from i-th and j-th traits, which is defined as

$VSI(I,j)= |S_i \cap S_j| / |S_i \cup S_j|$

where S_i and S_j denote sets of significant variants for the i-th and j-th traits, respectively. The VSI increases as two traits have more pleiotropic variants among their significant variants.

Pleiotropic variants with tissue-specific expression regulatory function

We annotated the gene symbol of the pleiotropic variant by using Ensemble database (ver. 101) [42]. In case of intergenic variants, we annotated the genes which were located the nearest in both directions of the variant. The single tissue eQTL data (RRID: SCR_013042, ver. 8) from the GTEx portal were used to investigate the eQTL of pleiotropic variants in Korea4K.

Investigation of potential causal relationships between traits based on Mendelian randomization (MR)

We used the Mendelian randomization method to investigate potential causal relationships among 1,332 combinations of an exposure trait and an outcome trait among 37 clinical traits. MR is computed from the linear regression analysis between the effects of SNPs on an exposure trait and their effects on an outcome trait. We chose the SNPs with suggestive WGWAS results (P-value < 10^{-5}) with exposure traits as the instrument variables. In case multiple SNPs existed in the LD block, the one with the smallest P-value was chosen. We rejected 40 SNPs, which were detected as outliers of linear regression from MR-PRESSO software (RRID: SCR_023697, ver. 1.0) [51] with 'NbDistribution=10000 and SignifThreshold=0.05' options, from further analysis. MR coefficients were computed using the chosen SNPs by three different methods: the Inverse-variance weighted (IVW) and MR-Egger method of TwoSampleMR package (RRID:SCR_019010, v.0.5.6) [52] and MR-PRESSO software (ver. 1.0) [51]. Finally, we selected 36 significant causal relationships that overlapped at least two of three methods (IVW, MR_Egger, and MRPRESSO). All analyses were performed with default options.

Acknowledgments

We appreciate all participants and Ulsan citizens who supported this project. We also thank Ju Yeon Park, Sangryoul Han, Jungae Shim, Nayoung Kim, Seung Gu Park, Byoung-Chul Kim, Jungeun Kim, Neung-hwa Park, Suan Cho, and Yeshin Park for supporting this project. We thank Young-woong Lee, Information Technology team in UNIST for supporting the data uploading to EGA. This work was supported by Biodatafarm computing infrastructure funded by the Ulsan metropolitan city government. We thank the Korea Institute of Science and Technology Information (KISTI) for providing us with the Korea Research Environment Open NETwork (KREONET). We thank our collaborators in NCSRD, KRISS, and C.-G. Kim. The Genotype-Tissue Expression (GTEx) Project was supported by the Common Fund of the Office of the Director of the National Institutes of Health, and by NCI, NHGRI, NHLBI, NIDA, NIMH, and NINDS. The data used for the analyses described in this manuscript were obtained from the GTEx Portal on 11/25/2021. This work was supported by the U-K BRAND Research Fund (1.200108.01) of UNIST (Ulsan National Institute of Science & Technology). This work was supported by the Research Project Funded by Ulsan City Research Fund (1.200047.01) of UNIST. This work was supported by the Promotion of Innovative Businesses for Regulation-Free Special Zones funded by the Ministry of SMEs and Startups (MSS, Korea) (1425157301 and 1425156792). This work was also supported by the Establishment of Demonstration Infrastructure for Regulation-Free Special Zones funded by the Ministry of SMEs and Startups (MSS, Korea) (1425157253). This research was also supported by the Technology Innovation Program (20016225, Development and Dissemination on National Standard Reference Data) funded by the Ministry of Trade, Industry & Energy (MOTIE, Korea). The biospecimens for this study were provided by Ulsan Medical Center and the Biobanks of Gyeongsang National University Hospital, Chungbuk National University

Hospital (18-27, 20-04), and Kyung Hee University Hospital (2018-4, 2019-4, 2019-6), the members of the National Biobank of Korea, which is supported by the Ministry of Health, Welfare and Family Affairs. 215 whole-genome-seq data used in this study were provided by the Clinical & Omics Data Archive (CODA), CODA accession number S000680 [53]. We thank Jaesu Bhak and Maryana Bhak for editing the manuscript.

Author contributions

S.J., Hansol C., Y. J., W.C. and Jong B. wrote the manuscript. S.J., Hansol C., Y. J., Hyunjoo C., K.A., H. R., Jihun. B., H. L., Yoonsung. K., S. H., C.L., and J. S. conducted the data analysis. C. K., Yeonkyung K., Younghui K., and Y. J. W. performed wet-lab experiments. S. J., Yeo Jin K., B. C. K., S.L., and Jong B. designed the study. S.J., Hansol C., Y.J. W.C., A.B., C.Y., D. B., O. B., E. S., S. K., J. P., J. J., D. J., S. L., and Jong B. revised the manuscript. S. L. and Jong B. jointly supervised the study.

Ethics, consents, and permissions

Sample collection and sequencing were approved by the Institutional Review Board (IRB) of the Ulsan National Institute of Science and Technology (UNISTIRB-15-19-A and UNISTIRB-16-13-C). The data employed in our study originates from voluntary blood or saliva donations, and we have diligently secured explicit, comprehensive consent forms from all participants prior to sample collection. These consent forms explicitly outline the intended use of their data for research purposes and underscore the voluntary nature of their participation. Furthermore, our study adheres to the ethical guidelines and regulations stipulated by the IRB. As a result, we can make the data of 3,839 individuals publicly available while respecting the privacy and consent of the participants.

Competing interests

S.J., Y. J., H. R., Y.J.K., C.K, Yeonkyung K., Younghui K., Y. J. W., and B. C. K. are employees and Jong B. is the CEO of Clinomics Inc. The authors declare no other competing interests.

Data Availability

Allele frequency information of variants is publicly available under the Korea4K webpage [54]. Raw sequencing data, individual genotype information, and clinical trait data will be as easily and freely available as possible upon request and after approval from the Korean Genomics Center's review board in UNIST. The raw sequencing data that can be distributed were uploaded to the European Genome-Phenome Archive under the study accession 'EGAS00001007580'. Information about the Korean Genome Project and other data sharing can be found at the Korea4K webpage All additional supporting data are available in the *GigaScience* repository, GigaDB [55].

Additional Files

Supplementary Fig. S1. Variants batch effect of DNA sequences.

Supplementary Fig. S2. Variants distribution based on variant location and allele frequency category in Korea4K.

Supplementary Fig. S3. Power comparison of whole-genome-wide association study between Korea4K and Korea1K.

Supplementary Fig. S4. Sequencing data quality metrics of Korea4K genomes.

Supplementary Fig. S5. QQplots for the whole-genome-wide association tests of the traits on the anthropometry category.

Supplementary Fig. S6. QQplots for the whole-genome-wide association tests of the traits on blood circulation biochemical category.

Supplementary Fig. S7. QQplots for the whole-genome-wide association tests of the traits on blood circulation physics category.

Supplementary Fig. S8. QQplots for the whole-genome-wide association tests of the traits on diabetes category.

Supplementary Fig. S9. QQplots for the whole-genome-wide association tests of the traits on electrolyte category.

Supplementary Fig. S10. QQplots for the whole-genome-wide association tests of the traits on hearing test category.

Supplementary Fig. S11. QQplots for the whole-genome-wide association tests of the traits on hematological category.

Supplementary Fig. S12. QQplots for the whole-genome-wide association tests of the traits on hepatitis category.

Supplementary Fig. S13. QQplots for the whole-genome-wide association tests of the traits on inflammation and etc category.

Supplementary Fig. S14. QQplots for the whole-genome-wide association tests of the traits on kidney function category.

Supplementary Fig. S15. QQplots for the whole-genome-wide association tests of the traits on liver function category.

Supplementary Fig. S16. QQplots for the whole-genome-wide association tests of the traits on pulmonary function category.

Supplementary Fig. S17. QQplots for the whole-genome-wide association tests of the traits on thyroid function category.

Supplementary Fig. S18. QQplots for the whole-genome-wide association tests of the traits on tumor biomarker category.

Supplementary Fig. S19. QQplots for the whole-genome-wide association tests of the traits on urinalysis category.

Supplementary Fig. S20. QQplots for the whole-genome-wide association tests of the traits on vision category.

Supplementary Table S1. Sample count in Korea4K.

Supplementary Table S2. Variant count in Korea4K before sample and variant filtering.

Supplementary Table S3. Variant count based on variant categories and reported to dbSNP.

Supplementary Table S4. Allele frequency information of populations for 62 Korean-specific missense variants

Supplementary Table S5. Prediction of changes in protein thermodynamic stability according to missense variant

Supplementary Table S6. List of the GWAS variants which have association significance $P< 5E-8$

Supplementary Table S7. Genetic heritability measurement

Supplementary Table S8. Genetic correlation measurement

Supplementary Table S9. Phenotypic correlation estimation

Supplementary Table S10. Pleiotropic variants

Supplementary Table S11. Mendelian randomization results

Supplementary Table S12. Statistics of clinical information

Supplementary Table S13. 26 traits with clinical calculations applied.

Supplementary Table S14. Statistical power measurement of 90 WGWAS tests

References

1. Song SO, Jung CH, Song YD, Park CY, Kwon HS, Cha BS, et al. Background and data configuration process of a nationwide population-based study using the korean national health insurance system. Diabetes Metab J. 2014;38 5:395-403. doi:10.4093/dmj.2014.38.5.395.
2. Jeon S, Bhak Y, Choi Y, Jeon Y, Kim S, Jang J, et al. Korean Genome Project: 1094 Korean personal genomes with clinical information. Sci Adv. 2020;6 22 doi:ARTN eaaz783510.1126/sciadv.aaz7835.
3. Kanai M, Akiyama M, Takahashi A, Matoba N, Momozawa Y, Ikeda M, et al. Genetic analysis of quantitative traits in the Japanese population links cell types to complex human diseases. Nat Genet. 2018;50 3:390-+. doi:10.1038/s41588-018-0047-6.
4. Choe EK, Shivakumar M, Verma A, Verma SS, Choi SH, Kim JS, et al. Leveraging deep phenotyping from health check-up cohort with 10,000 Korean individuals for phenome-wide association study of 136 traits. Sci Rep-Uk. 2022;12 1 doi:ARTN 193010.1038/s41598-021-04580-2.
5. Van Hout CV, Tachmazidou I, Backman JD, Hoffman JD, Liu D, Pandey AK, et al. Exome sequencing and characterization of 49,960 individuals in the UK Biobank. Nature. 2020;586 7831:749-+. doi:10.1038/s41586-020-2853-0.
6. Jiang LD, Zheng ZL, Qi T, Kemper KE, Wray NR, Visscher PM, et al. A resource-efficient tool for mixed model association analysis of large-scale data. Nat Genet. 2019;51 12:1749-+. doi:10.1038/s41588-019-0530-8.
7. Jumper J, Evans R, Pritzel A, Green T, Figurnov M, Ronneberger O, et al. Highly accurate protein structure prediction with AlphaFold. Nature. 2021;596 7873:583-+. doi:10.1038/s41586-021-03819-2.
8. Buniello A, MacArthur JAL, Cerezo M, Harris LW, Hayhurst J, Malangone C, et al. The NHGRI-EBI GWAS Catalog of published genome-wide association studies, targeted arrays and summary statistics 2019. Nucleic Acids Res. 2019;47 D1:D1005-D12. doi:10.1093/nar/gky1120.
9. Seyed Khoei N, Jenab M, Murphy N, Banbury BL, Carreras-Torres R, Viallon V, et al. Circulating bilirubin levels and risk of colorectal cancer: serological and Mendelian randomization analyses. BMC Med. 2020;18 1:229. doi:10.1186/s12916-020-01703-w.
10. Chang MH, Yesupriya A, Ned RM, Mueller PW and Dowling NF. Genetic variants associated with fasting blood lipids in the U.S. population: Third National Health and Nutrition Examination Survey. BMC Med Genet. 2010;11:62. doi:10.1186/1471-2350-11-62.
11. UK Biobank. http://www.nealelab.is/uk-biobank. Accessed 3 April 2022.

12. Canela-Xandri O, Rawlik K and Tenesa A. An atlas of genetic associations in UK Biobank. Nat Genet. 2018;50 11:1593-9. doi:10.1038/s41588-018-0248-z.
13. Khodayari S, Sadeghi O, Safabakhsh M and Mozaffari-Khosravi H. Meat consumption and the risk of general and central obesity: the Shahedieh study. BMC Res Notes. 2022;15 1:339. doi:10.1186/s13104-022-06235-5.
14. Pimenta E, Jensen M, Jung D, Schaumann F, Boxnick S and Truebel H. Effect of Diet on Serum Creatinine in Healthy Subjects During a Phase I Study. J Clin Med Res. 2016;8 11:836-9. doi:10.14740/jocmr2738w.
15. Sodini SM, Kemper KE, Wray NR and Trzaskowski M. Comparison of Genotypic and Phenotypic Correlations: Cheverud's Conjecture in Humans. Genetics. 2018;209 3:941-8. doi:10.1534/genetics.117.300630.
16. Guo S, Lv HT, Yan L and Rong FN. Hyperamylasemia may indicate the presence of ovarian carcinoma A case report. Medicine. 2018;97 49 doi:ARTN e13520
10.1097/MD.0000000000013520.
17. Shintani D, Yoshida H, Imai Y and Fujiwara K. Acute pancreatitis induced by paclitaxel and carboplatin therapy in an ovarian cancer patient. Eur J Gynaecol Oncol. 2016;37 2:286-7.
18. Zakrzewska I and Pietryńczak M. The activity of alpha-amylase and its salivary isoenzymes in serum and urine of patients with neoplastic diseases of female reproductive organs. Roczniki Akademii Medycznej w Bialymstoku (1995). 1996;41 2:492-8.
19. Hemani G, Bowden J and Davey Smith G. Evaluating the potential role of pleiotropy in Mendelian randomization studies. Hum Mol Genet. 2018;27 R2:R195-R208. doi:10.1093/hmg/ddy163.
20. Pulit SL, Stoneman C, Morris AP, Wood AR, Glastonbury CA, Tyrrell J, et al. Meta-analysis of genome-wide association studies for body fat distribution in 694 649 individuals of European ancestry. Hum Mol Genet. 2019;28 1:166-74. doi:10.1093/hmg/ddy327.
21. Ebrahim S and Davey Smith G. Mendelian randomization: can genetic epidemiology help redress the failures of observational epidemiology? Hum Genet. 2008;123 1:15-33. doi:10.1007/s00439-007-0448-6.
22. Aabo K, Pedersen H and Kjaer M. Carcinoembryonic antigen (CEA) and alkaline phosphatase in progressive colorectal cancer with special reference to patient survival. Eur J Cancer Clin Oncol. 1986;22 2:211-7. doi:10.1016/0277-5379(86)90033-7.
23. Tartter PI, Slater G, Gelernt I and Aufses AH, Jr. Screening for liver metastases from colorectal cancer with carcinoembryonic antigen and alkaline phosphatase. Ann Surg. 1981;193 3:357-60. doi:10.1097/00000658-198103000-00019.
24. Walach N, Guterman A, Zaidman JL, Kaufman S and Scharf S. Leukocyte alkaline phosphatase and carcinoembryonic antigen in breast cancer patients: clinical correlation with the markers. J Surg Oncol. 1989;40 2:85-7. doi:10.1002/jso.2930400205.
25. Forouhi NG, Sattar N and McKeigue PM. Relation of C-reactive protein to body fat distribution and features of the metabolic syndrome in Europeans and South Asians. Int J Obes Relat Metab Disord. 2001;25 9:1327-31. doi:10.1038/sj.ijo.0801723.
26. Lim S, Jang HC, Lee HK, Kimm KC, Park C and Cho NH. The relationship between body fat and C-reactive protein in middle-aged Korean population. Atherosclerosis. 2006;184 1:171-7. doi:10.1016/j.atherosclerosis.2005.04.003.

27. Lee CM, Huxley RR, Wildman RP and Woodward M. Indices of abdominal obesity are better discriminators of cardiovascular risk factors than BMI: a meta-analysis. J Clin Epidemiol. 2008;61 7:646-53. doi:10.1016/j.jclinepi.2007.08.012.
28. Yang JA, Lee SH, Goddard ME and Visscher PM. GCTA: A Tool for Genome-wide Complex Trait Analysis. Am J Hum Genet. 2011;88 1:76-82. doi:10.1016/j.ajhg.2010.11.011.
29. Zhang YL, Cheng YS, Jiang W, Ye YX, Lu QS and Zhao HY. Comparison of methods for estimating genetic correlation between complex traits using GWAS summary statistics. Brief Bioinform. 2021;22 5 doi:ARTN bbaa44210.1093/bib/bbaa442.
30. Visscher PM, Hemani G, Vinkhuyzen AA, Chen GB, Lee SH, Wray NR, et al. Statistical power to detect genetic (co)variance of complex traits using SNP data in unrelated samples. PLoS Genet. 2014;10 4:e1004269. doi:10.1371/journal.pgen.1004269.
31. Li J, Gui L, Wu C, He Y, Zhou L, Guo H, et al. Genome-wide association study on serum alkaline phosphatase levels in a Chinese population. BMC Genomics. 2013;14:684. doi:10.1186/1471-2164-14-684.
32. Middelberg RP, Ferreira MA, Henders AK, Heath AC, Madden PA, Montgomery GW, et al. Genetic variants in LPL, OASL and TOMM40/APOE-C1-C2-C4 genes are associated with multiple cardiovascular-related traits. BMC Med Genet. 2011;12:123. doi:10.1186/1471-2350-12-123.
33. Jeon Y, Jeon S, Choi WH, An K, Choi H, Kim BC, et al. Genome-wide analyses of early-onset acute myocardial infarction identify 29 novel loci by whole genome sequencing. Hum Genet. 2023;142 2:231-43. doi:10.1007/s00439-022-02495-0.
34. Nurk S, Koren S, Rhie A, Rautiainen M, Bzikadze AV, Mikheenko A, et al. The complete sequence of a human genome. Science. 2022;376 6588:44-53. doi:10.1126/science.abj6987.
35. Liao WW, Asri M, Ebler J, Doerr D, Haukness M, Hickey G, et al. A draft human pangenome reference. Nature. 2023;617 7960:312-24. doi:10.1038/s41586-023-05896-x.
36. Martin M. Cutadapt removes adapter sequences from high-throughput sequencing reads. EMBnet journal. 2011;17 1:10-2.
37. Li H and Durbin R. Fast and accurate short read alignment with Burrows-Wheeler transform. Bioinformatics. 2009;25 14:1754-60. doi:10.1093/bioinformatics/btp324.
38. Poplin R, Ruano-Rubio V, DePristo MA, Fennell TJ, Carneiro MO, Van der Auwera GA, et al. Scaling accurate genetic variant discovery to tens of thousands of samples. BioRxiv. 2018:201178.
39. Purcell S, Neale B, Todd-Brown K, Thomas L, Ferreira MAR, Bender D, et al. PLINK: A tool set for whole-genome association and population-based linkage analyses. Am J Hum Genet. 2007;81 3:559-75. doi:10.1086/519795.
40. McLaren W, Gil L, Hunt SE, Riat HS, Ritchie GR, Thormann A, et al. The ensembl variant effect predictor. Genome biology. 2016;17 1:1-14.
41. Genomes Project C, Abecasis GR, Altshuler D, Auton A, Brooks LD, Durbin RM, et al. A map of human genome variation from population-scale sequencing. Nature. 2010;467 7319:1061-73. doi:10.1038/nature09534.
42. Cunningham F, Allen JE, Allen J, Alvarez-Jarreta J, Amode MR, Armean IM, et al. Ensembl 2022. Nucleic Acids Res. 2022;50 D1:D988-D95. doi:10.1093/nar/gkab1049.

43. Blum M, Chang HY, Chuguransky S, Grego T, Kandasaamy S, Mitchell A, et al. The InterPro protein families and domains database: 20 years on. Nucleic Acids Res. 2021;49 D1:D344-D54. doi:10.1093/nar/gkaa977.
44. Delgado J, Radusky LG, Cianferoni D and Serrano L. FoldX 5.0: working with RNA, small molecules and a new graphical interface. Bioinformatics. 2019;35 20:4168-9. doi:10.1093/bioinformatics/btz184.
45. McCarthy S, Das S, Kretzschmar W, Delaneau O, Wood AR, Teumer A, et al. A reference panel of 64,976 haplotypes for genotype imputation. Nat Genet. 2016;48 10:1279-83. doi:10.1038/ng.3643.
46. Das S, Forer L, Schonherr S, Sidore C, Locke AE, Kwong A, et al. Next-generation genotype imputation service and methods. Nat Genet. 2016;48 10:1284-7. doi:10.1038/ng.3656.
47. Chang CC, Chow CC, Tellier LCAM, Vattikuti S, Purcell SM and Lee JJ. Second-generation PLINK: rising to the challenge of larger and richer datasets. Gigascience. 2015;4 doi:ARTN 710.1186/s13742-015-0047-8.
48. Shungin D, Winkler TW, Croteau-Chonka DC, Ferreira T, Locke AE, Magi R, et al. New genetic loci link adipose and insulin biology to body fat distribution. Nature. 2015;518 7538:187-96. doi:10.1038/nature14132.
49. Yang J, Weedon MN, Purcell S, Lettre G, Estrada K, Willer CJ, et al. Genomic inflation factors under polygenic inheritance. Eur J Hum Genet. 2011;19 7:807-12. doi:10.1038/ejhg.2011.39.
50. Lee S, Yang J, Goddard M, Visscher P and Wray N. Estimation of pleiotropy between complex diseases using SNP-derived genomic relationships and restricted maximum likelihood. Bioinformatics. 2012;28 19:2540-2.
51. Verbanck M, Chen CY, Neale B and Do R. Detection of widespread horizontal pleiotropy in causal relationships inferred from Mendelian randomization between complex traits and diseases. Nat Genet. 2018;50 5:693-+. doi:10.1038/s41588-018-0099-7.
52. Hemani G, Zhengn J, Elsworth B, Wade KH, Haberland V, Baird D, et al. The MR-Base platform supports systematic causal inference across the human phenome. Elife. 2018;7 doi:ARTN e3440810.7554/eLife.34408.
53. CODA. http://coda.nih.go.kr. Accessed October 2018.
54. Korea4K Genomes. http://koreangenome.org/Korea4K_Genomes. Accessed 13 March 2024.
55. Jeon S, Choi H, Jeon Y, Choi W, Choi H, An K, et al. Supporting data for "Korea4K: whole genome sequences of 4,157 Koreans with 107 phenotypes derived from extensive health check-ups". GigaScience Database. 2024. doi:dx.doi.org/10.5524/102507.

Figure1

Click here to access/download;Figure;Figure1.pdf

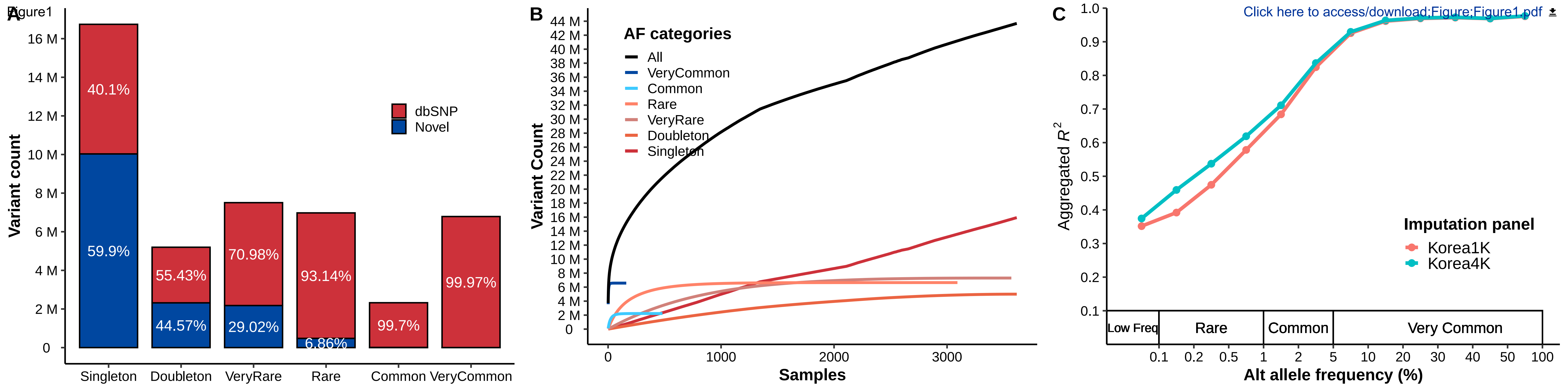

Click here to access/download;Figure;Figure2.pdf

Figure2

A

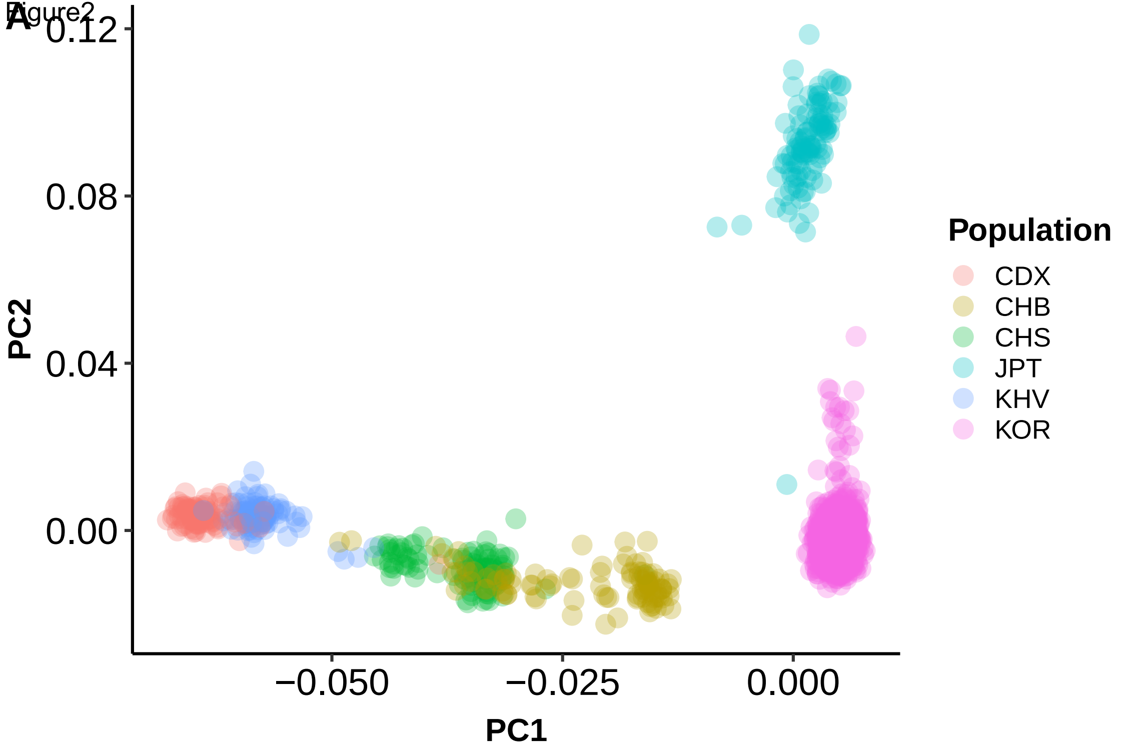

B

Population	rs1141967(TPSD1)	rs12420076(OR9G1)	rs14456901(GDF2)	rs155826592(MAGEA3)	rs200581589(HLA−DRB5)	rs201779716(PRSS2)	rs201790399(LIX1L)	rs4990121(OR8U1)	rs569378041(RBP3)	rs587669051(PDZK1)	rs71497225(MRC1)	rs78574933(IGHV4−4)
Korea4K	0.58	0.49	0	0	0.04	0.13	0	0.49	0	0	1	0
JPT	0.82	0.04	0.99	1	0.26	1	0.99	0.15	1	1	0.5	0.62
CHB	0.79	0.06	0.99	1	0.27	1	1	0.13	1	1	0.56	0.6
CHS	0.84	0.06	0.99	1	0.24	0.99	1	0.2	1	1	0.54	0.56
CDX	0.78	0.04	0.98	1	0.12	1	1	0.12	1	1	0.55	0.58
KHV	0.73	0.02	0.97	1	0.32	1	1	0.2	0.99	1	0.56	0.7
EAS	0.79	0.04	0.98	1	0.24	1	1	0.16	1	1	0.54	0.61
SAS	0.46	0.04	1	1	0.16	1	1	0.12	1	1	0.14	0.59
EUR	0.32	0.05	1	1	0.24	1	1	0.13	1	1	0.12	0.69
AMR	0.3	0.06	1	1	0.24	1	1	0.16	1	1	0.15	0.7
AFR	0.33	0.12	1	0.99	0.36	1	1	0.13	1	1	0.41	0.92

Figure3 Click here to access/download;Figure;Figure3.pdf

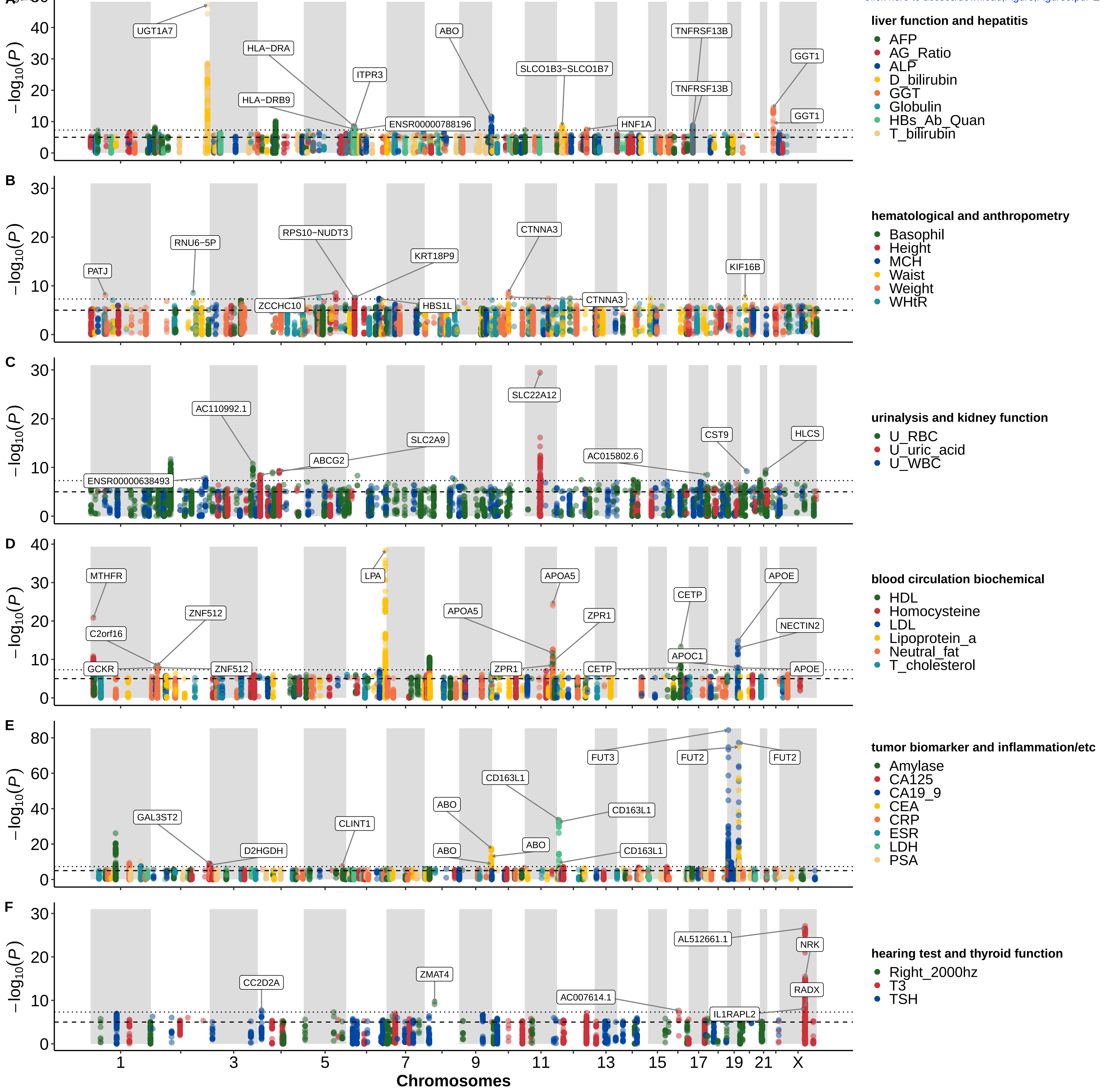

Figure4

Click here to access/download;Figure;Figure4.pdf

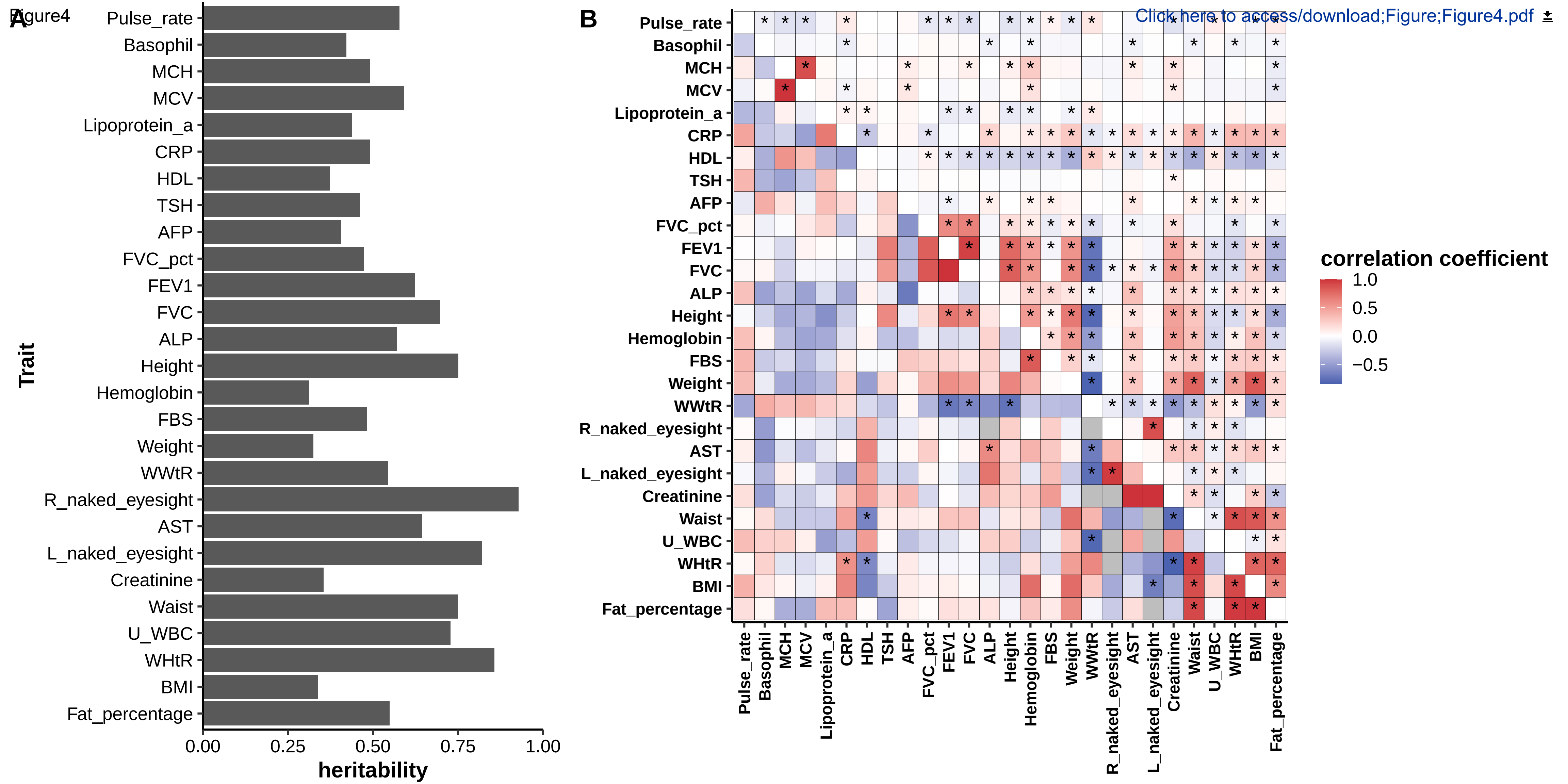

Figure5

Click here to access/download;Figure;Figure5.pdf

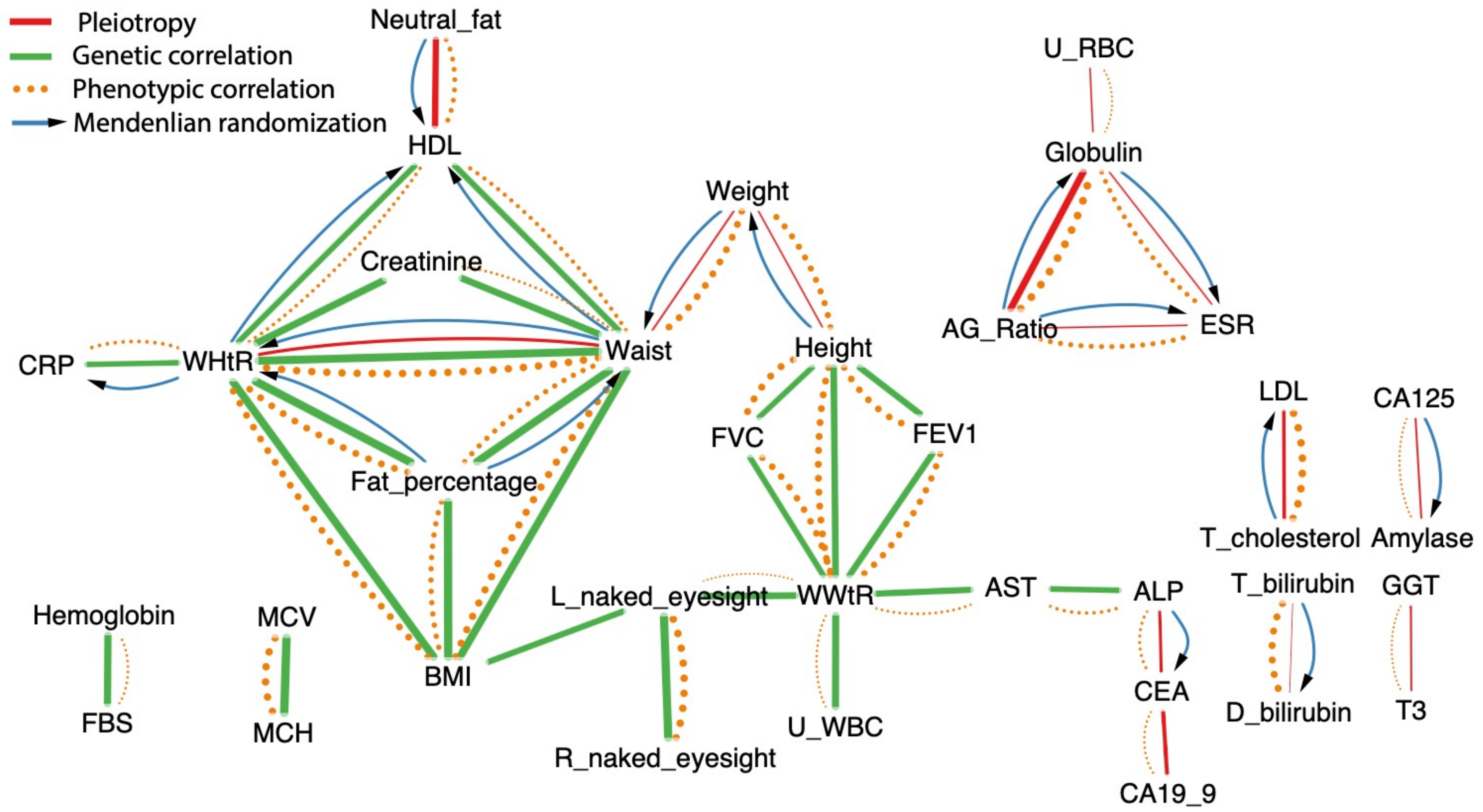

Supplementary Table S1

Click here to access/download
Supplementary Material
Korea4K_TableS1_Supplementary_Material.xlsx

Supplementary Table S2

Click here to access/download
Supplementary Material
Korea4K_TableS2_Supplementary_Material.xlsx

Supplementary Table S3

Click here to access/download
Supplementary Material
Korea4K_TableS3_Supplementary_Material.xlsx

Supplementary Table S4

Click here to access/download
Supplementary Material
Korea4K_TableS4_Supplementary_Material.xlsx

Supplementary Table S5

Click here to access/download
Supplementary Material
Korea4K_TableS5_Supplementary_Material.xlsx

Supplementary Table S6

Click here to access/download
Supplementary Material
Korea4K_TableS6_Supplementary_Material.xlsx

Supplementary Table S7

Click here to access/download
Supplementary Material
Korea4K_TableS7_Supplementary_Material.xlsx

Supplementary Table S8

Click here to access/download

Supplementary Material

Korea4K_TableS8_Supplementary_Material.xlsx

Supplementary Table S9

Click here to access/download
Supplementary Material
Korea4K_TableS9_Supplementary_Material.xlsx

Supplementary Table S10

Click here to access/download
Supplementary Material
Korea4K_TableS10_Supplementary_Material.xlsx

Supplementary Table S11

Click here to access/download
Supplementary Material
Korea4K_TableS11_Supplementary_Material.xlsx

Supplementary Table S12

Click here to access/download
Supplementary Material
Korea4K_TableS12_Supplementary_Material.xlsx

Supplementary Table S13

Click here to access/download
Supplementary Material
Korea4K_TableS13_Supplementary_Material.xlsx

Supplementary Table S14

Click here to access/download
Supplementary Material
Korea4K_TableS14_Supplementary_Material.xlsx

Supplementary Figures

Click here to access/download
Supplementary Material
Korea4K_Figures_Supplementary_Material.docx

Cover Letter

Click here to access/download;Personal Cover;Korea4K_Coverletter_GS_20230425.docx

Dear Editor,

We would like to submit our manuscript entitled "**Korea4K: whole genome sequences of 4,157 Koreans with 107 phenotypes derived from extensive health check-ups**" as a *Research Article* in *GigaScience*.

Since we reported Korea1K (1,094 Korean genomes with 79 clinical traits) in 2020 (Jeon et al., *Sci Adv*. 2020), we have pursued a more comprehensive study based on a larger cohort of Koreans (4,157 whole genomes with 107 clinical traits) as the second phase of the Korean Genome Project (KGP).

Here, we found that only around 4,000 whole genomes (Korea4K) were sufficient to cover the genomic diversity of the Korean population with East Asian ancestry by analyzing the statistics of common and rare SNP variants. We also present the Korea4K variome database as a part of the KGP, which could be a resource for a large-scale population genomics analysis of diverse ethnic groups in association with human evolution and diseases.

The major difference between Korea1K and Korear4K is not only in the sample size but also in the number of clinical traits derived from extensively curated reports covering the most common health check-up parameters. With the greater number of samples and clinical traits, we were able to identify 1,356 new associations between genotypes and phenotypes, which had not been detected in Korea1K. Furthermore, we performed genetic correlation, pleiotropy, and Mendelian randomization analyses to map the variome with the clinical traits from common health check-ups. We also confirmed that Korea4K, compared to Korea1K, could improve quality as a reference panel for genotype imputation.

As our study provides a possibly useful resource for exploring the relationship between the genome and the phenome, and the variome data will be publicly available as open as possible, we believe that this manuscript fits the scope of *GigaScience*.

All study participants provided informed consent, and the study design was approved by the appropriate ethics review board.

S.J., Y. J., H. R., Y.J.K., C.K, Yeonkyung K., Younghui K., Y. J. W., and B. C. K. are employees and Jong B. is the CEO of Clinomics Inc. The authors declare no other competing interests.

We confirm that all authors have approved the manuscript for submission and the content of the manuscript has not been published, or submitted for publication elsewhere.

We would like to suggest the following reviewers:

- Tim Hubbard, Ph.D., Professor of Bioinformatics and Head of Department, Department of Medical & Molecular Genetics at King's College London, tim.hubbard@kcl.ac.uk
- Masao Nagasaki, Ph.D., Professor at the Center for Genomic Medicine, Kyoto University, nagasaki@genome.med.kyoto-u.ac.jp

Thank you for your consideration.

Sincerely,
Jong Bhak, Ph.D.

Korean Genomics Center
Ulsan National Institute of Science and Technology
Ulsan 44919, Republic of Korea
Email: jongbhak@genomics.org
Tel: +82 (0)10 4644 6754

 Click here to access/download;Personal Cover;Korea4K_Coverletter_revision1_20231129.docx

Re: “**Korea4K: whole genome sequences of 4,157 Koreans with 107 phenotypes derived from extensive health check-ups**”

Dear *Giga Science* Editor,

We would like to express our appreciation for the constructive feedback provided by the reviewers and editorial team. Please find our revision note and a revised version of our manuscript “**Korea4K: whole genome sequences of 4,157 Koreans with 107 phenotypes derived from extensive health check-ups”.**

We have checked and tried to accommodate all the critical points and suggestions from the reviewers and you.

As a significant addition, we have clarified the methods of sequencing data and clinical data preprocessing. Also, we have investigated the results of the phenomics analyses and published literature and extended additional discussion points in the discussion sections. We have also been uploading the sequencing raw data of 4K samples to the European Genome-Phenome Archive (EGA) during the revision to make the scientific community access our data conveniently. Despite the very large size of the whole data (~120TB), we think we can finish the data upload on time.

Our manuscript has been modified in the abstract, results, discussion, and method to better represent the changes made according to all the reviewers’ criticism and suggestions.

We think that these revisions address the concerns raised during the initial review and significantly enhance the scientific quality of the manuscript. Our study contributes to the understanding of the relationship between the genome and the phenome, providing a valuable resource for the scientific community.

Thank you very much for your consideration.

Sincerely,
Jong Bhak, Ph.D.

Korean Genomics Center
Ulsan National Institute of Science and Technology
Ulsan 44919, Republic of Korea
Email: jongbhak@genomics.org
Tel: +82 (0)10 4644 6754
