# Supplementary material for: Korea4K: whole genome sequences of 4,157 Koreans with 107 phenotypes derived from extensive health check-ups
Source: Gigascience. 2024 Apr 16;13:giae014. doi: 10.1093/gigascience/giae014 (PMC11020240; doi:10.1093/gigascience/giae014)
Supplement: giae014_Supplemental_Files [file giae014_supplemental_files.zip › Korea4K_Figures_Supplementary_Material.docx]

**Supplementary figures**


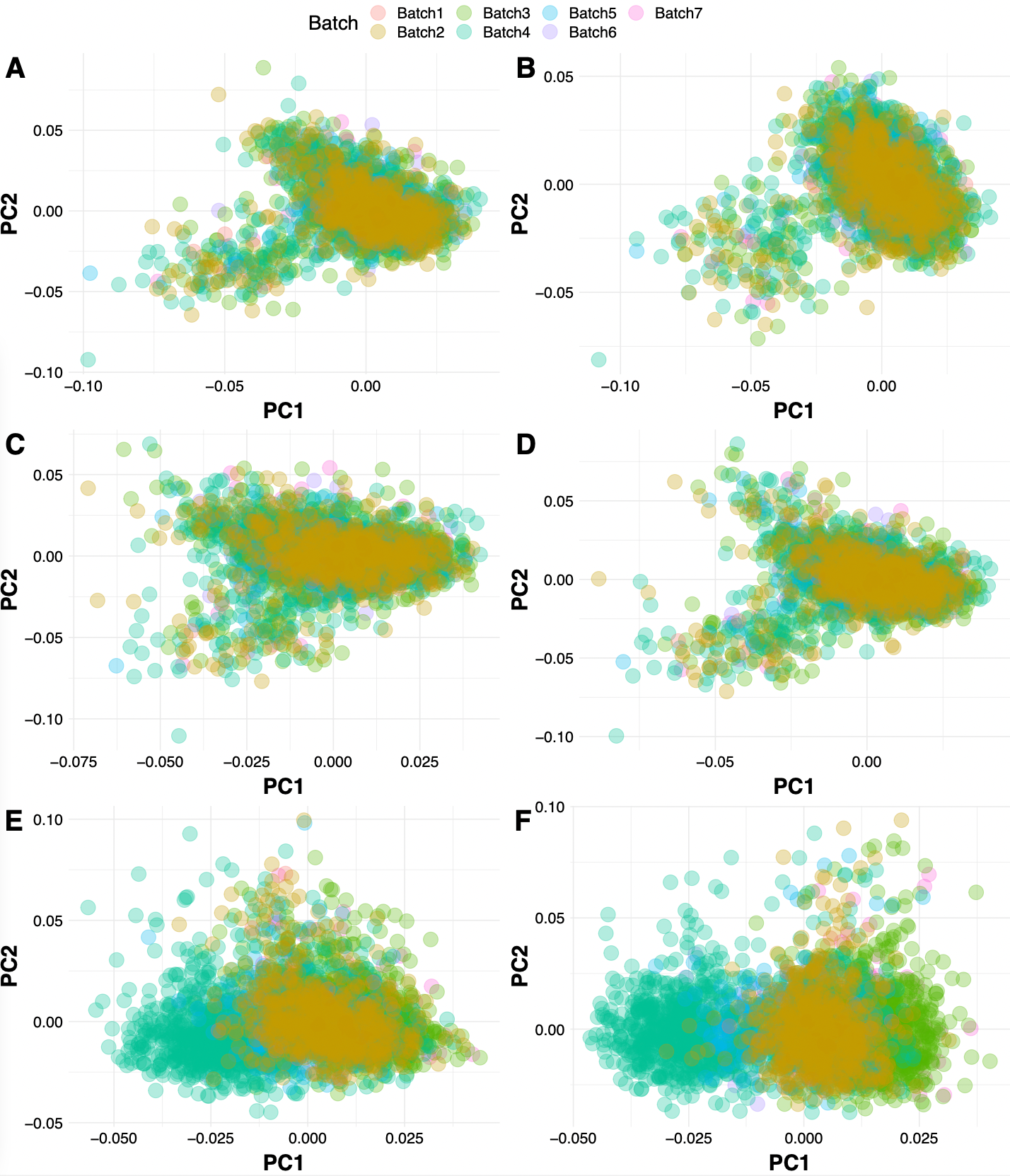


**Supplementary Fig. S1.** Variants batch effect of DNA sequences. Principal component analysis (PCA) plots using variants in Korea4K set based on different allele balance (AB) standard deviation (SD) filtering criteria. (A) cutoff of average AB ± 0.25 × SD (B) cutoff of average AB ± 0.5 × SD (C) cutoff of average AB ± 0.75 × SD (D) cutoff of average AB ± 1.0 × SD (E) cutoff of average AB ± 1.5 × SD (F) cutoff of average AB ± 2.0 × SD

**Supplementary Fig. S2.** Variants distribution based on variant location and allele frequency category in Korea4K (A) Variants counts and (B) proportions of the number of variants based on allele frequency categories. IGR: inter-genic region except for 5’ and 3’ Flank variants; UTR: untranslated region. Singleton, allele count = 1; doubleton, allele count = 2; very rare, allele count of > 2 and allele frequency of ≤ 0.001; rare, allele frequency of > 0.001 and allele frequency of ≤ 0.01; common, allele frequency of > 0.01 and allele frequency of ≤ 0.05; very common, allele frequency of > 0.05.

**Supplementary Fig. S3.** Power comparison of whole-genome-wide association study between Korea4K and Korea1K. X-axis indicates the fold change of beta value or odd ratio of Korea4K compared to Korea1K. Y-axis indicates the fold change of -log_10_(*P*) value of Korea4K compared to Korea1K.

**Supplementary Fig. S4.** Mapping depth distribution of Korea4K genomes.


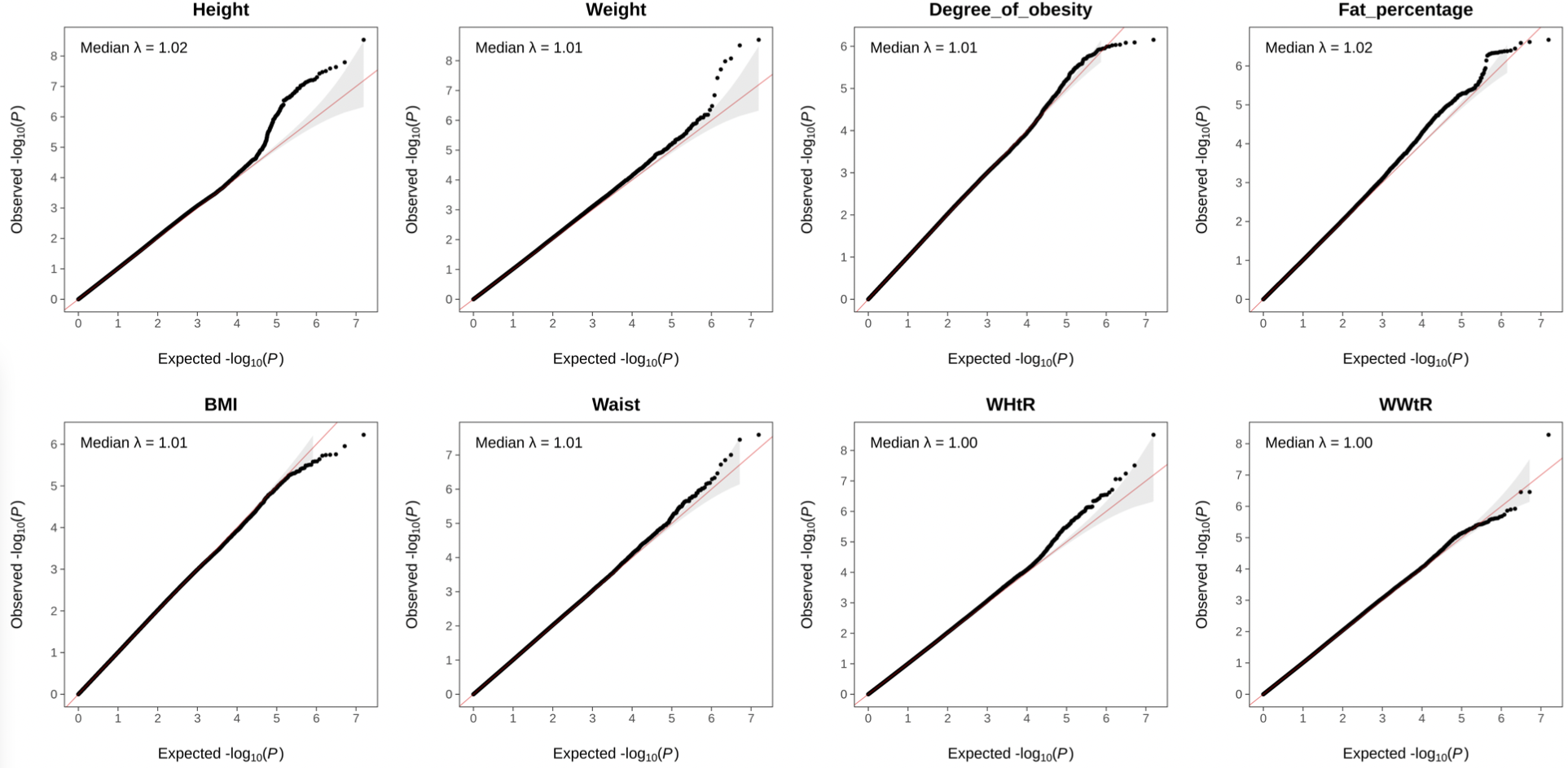


**Supplementary Fig. S5.** QQplots for the whole-genome-wide association tests of the traits on the anthropometry category. X-axis indicates the expected -log_10_ *P*-value. Y-axis indicates observed -log_10_ *P*-value.


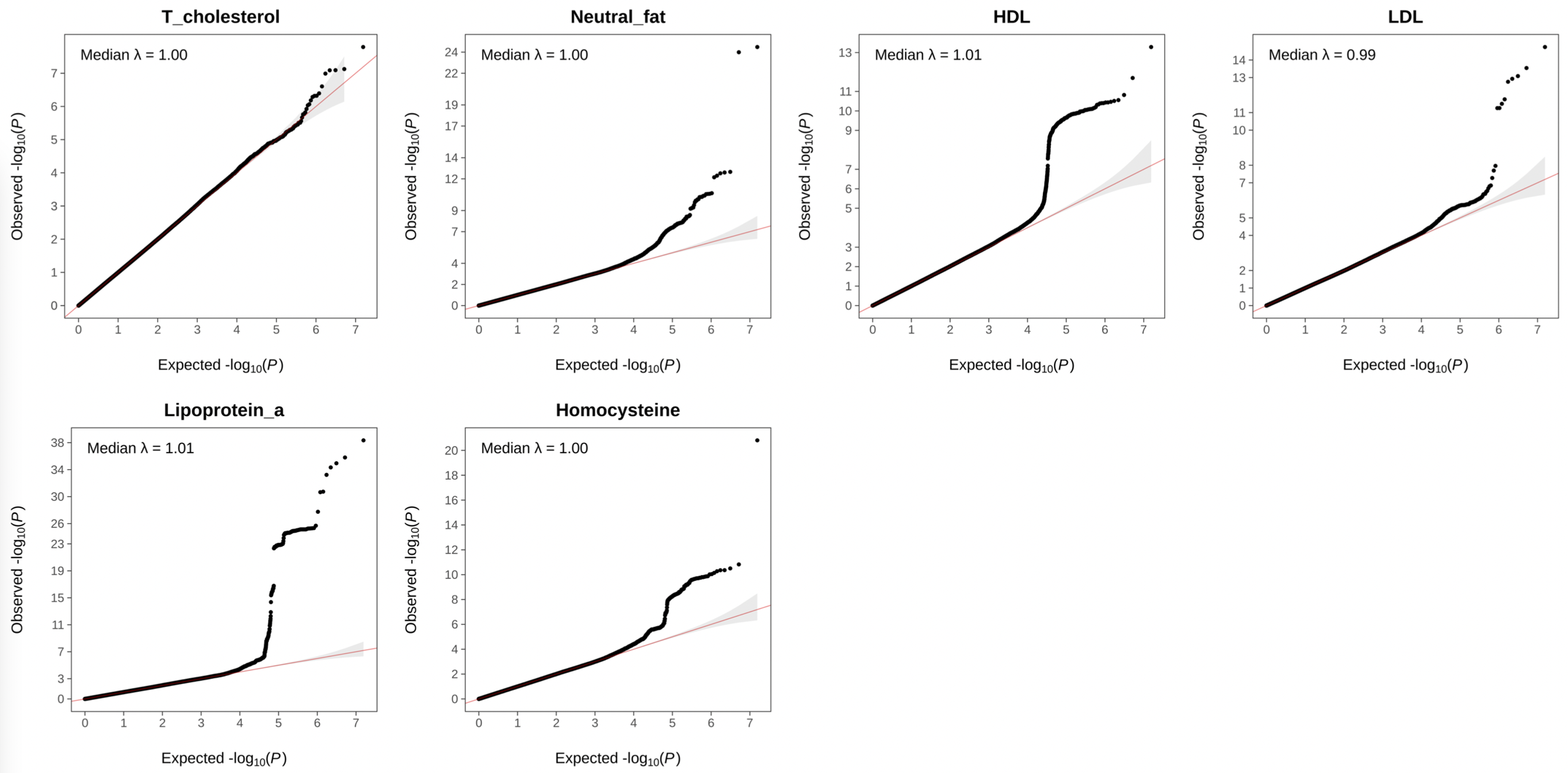


**Supplementary Fig. S6.** QQplots for the whole-genome-wide association tests of the traits on blood circulation biochemical category. X-axis indicates the expected -log_10_ *P*-value. Y-axis indicates observed -log_10_ *P*-value.


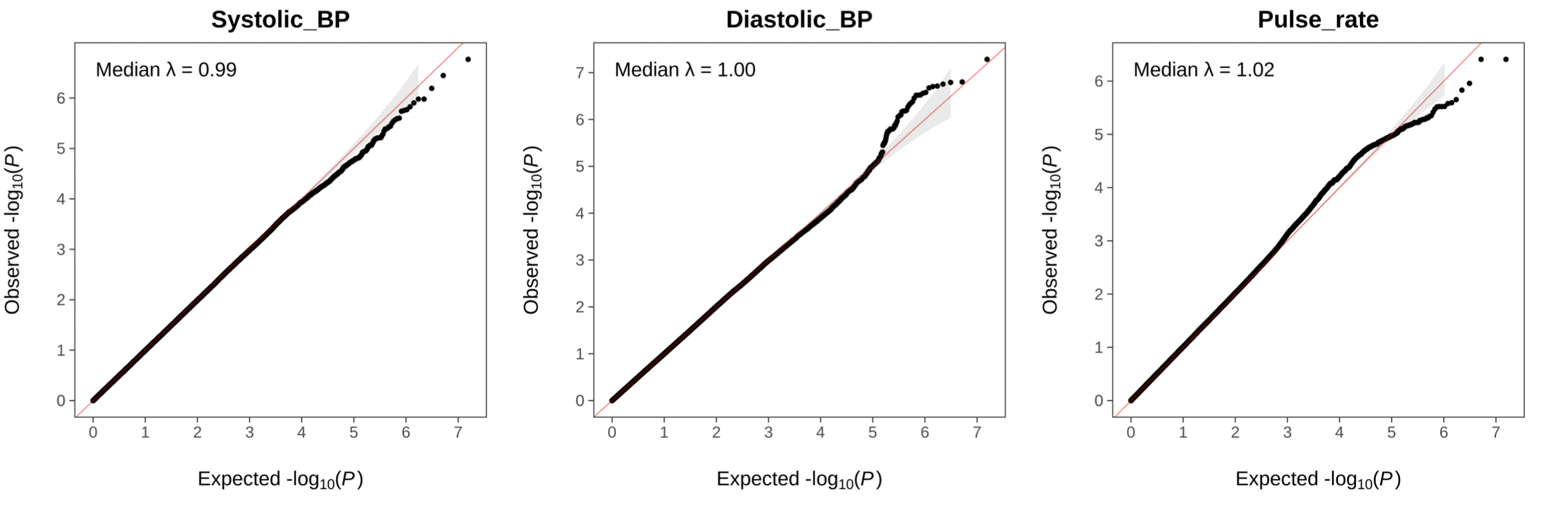


**Supplementary Fig. S7.** QQplots for the whole-genome-wide association tests of the traits on blood circulation physics category. X-axis indicates the expected -log_10_ *P*-value. Y-axis indicates observed -log_10_ *P*-value.


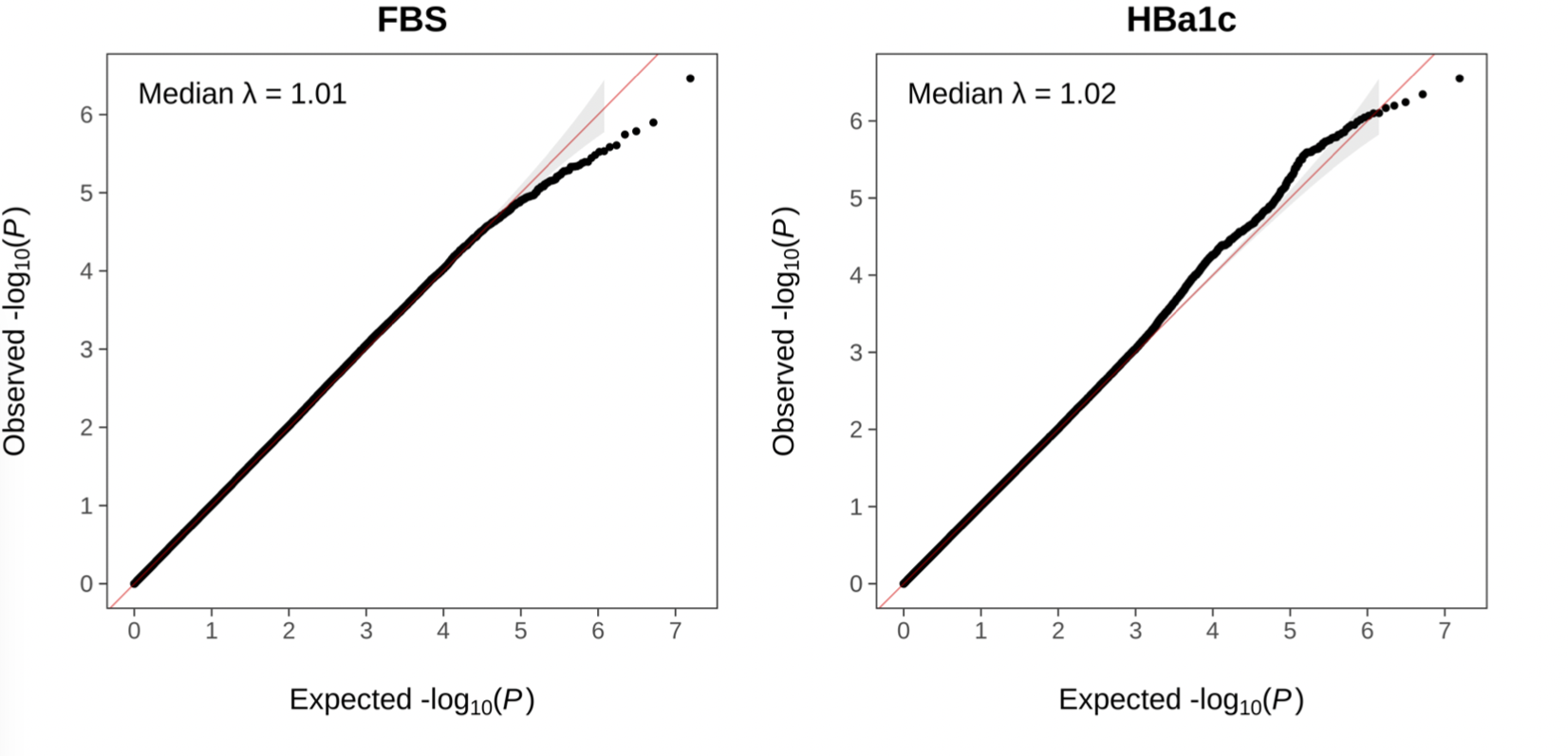


**Supplementary Fig. S8.** QQplots for the whole-genome-wide association tests of the traits on diabetes category. X-axis indicates the expected -log_10_ *P*-value. Y-axis indicates observed -log_10_ *P*-value.


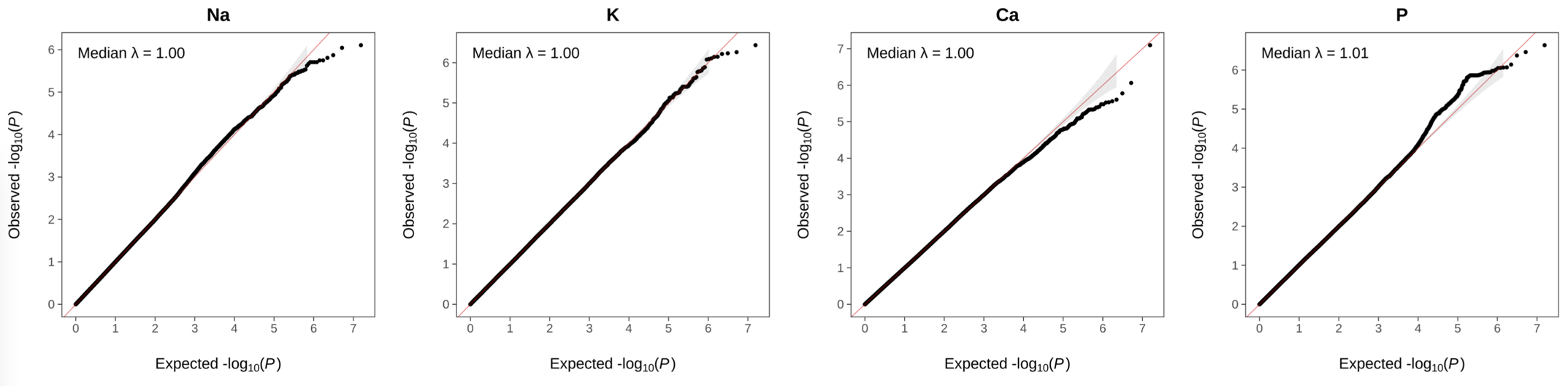


**Supplementary Fig. S9.** QQplots for the whole-genome-wide association tests of the traits on electrolyte category. X-axis indicates the expected -log_10_ *P*-value. Y-axis indicates observed -log_10_ *P*-value.


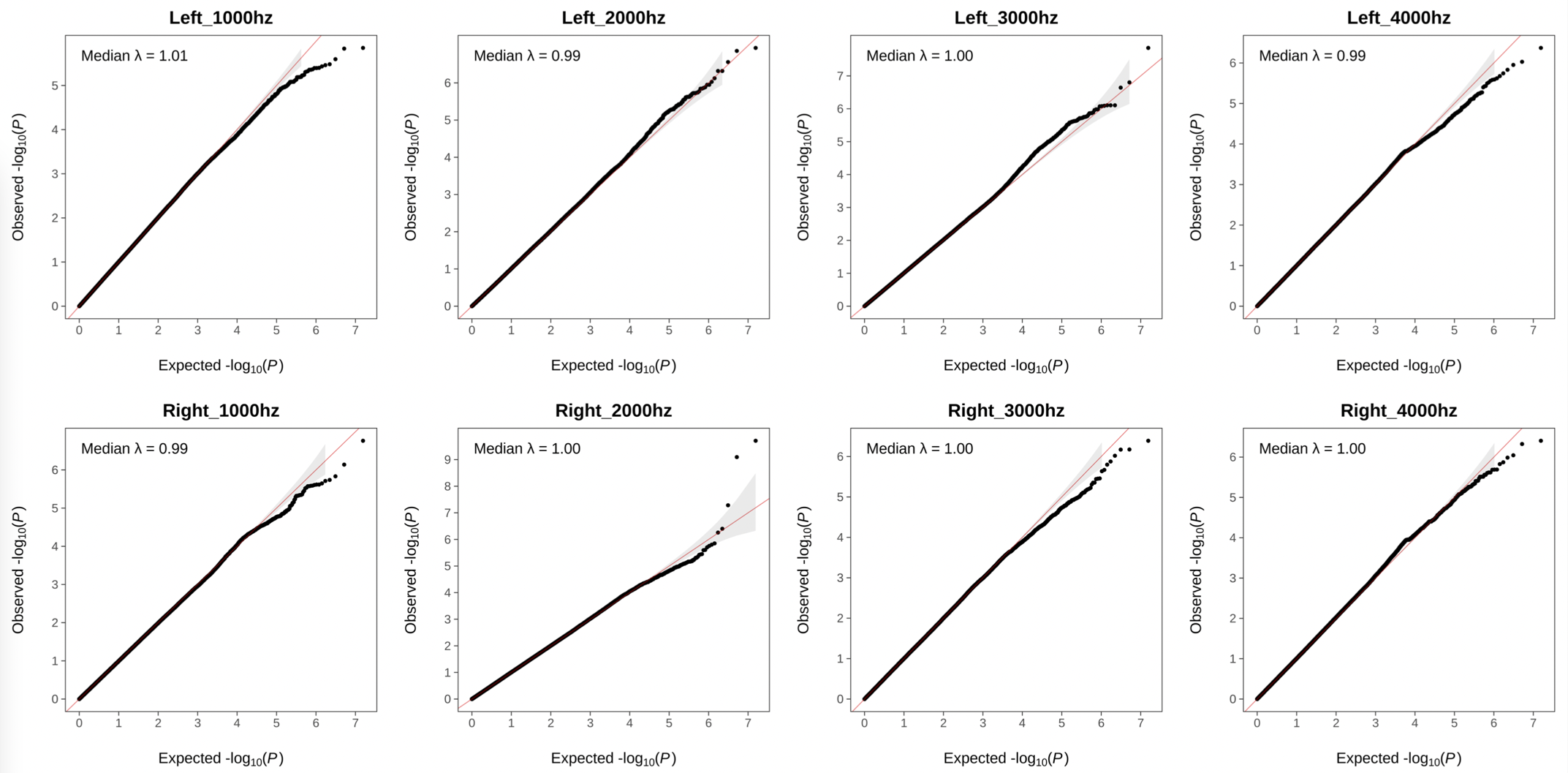


**Supplementary Fig. S10.** QQplots for the whole-genome-wide association tests of the traits on hearing test category. X-axis indicates the expected -log_10_ *P*-value. Y-axis indicates observed -log_10_ *P*-value.

**Supplementary Fig. S11.** QQplots for the whole-genome-wide association tests of the traits on hematological category. X-axis indicatesthe expected -log_10_ *P*-value. Y-axis indicates observed -log_10_ *P*-value.


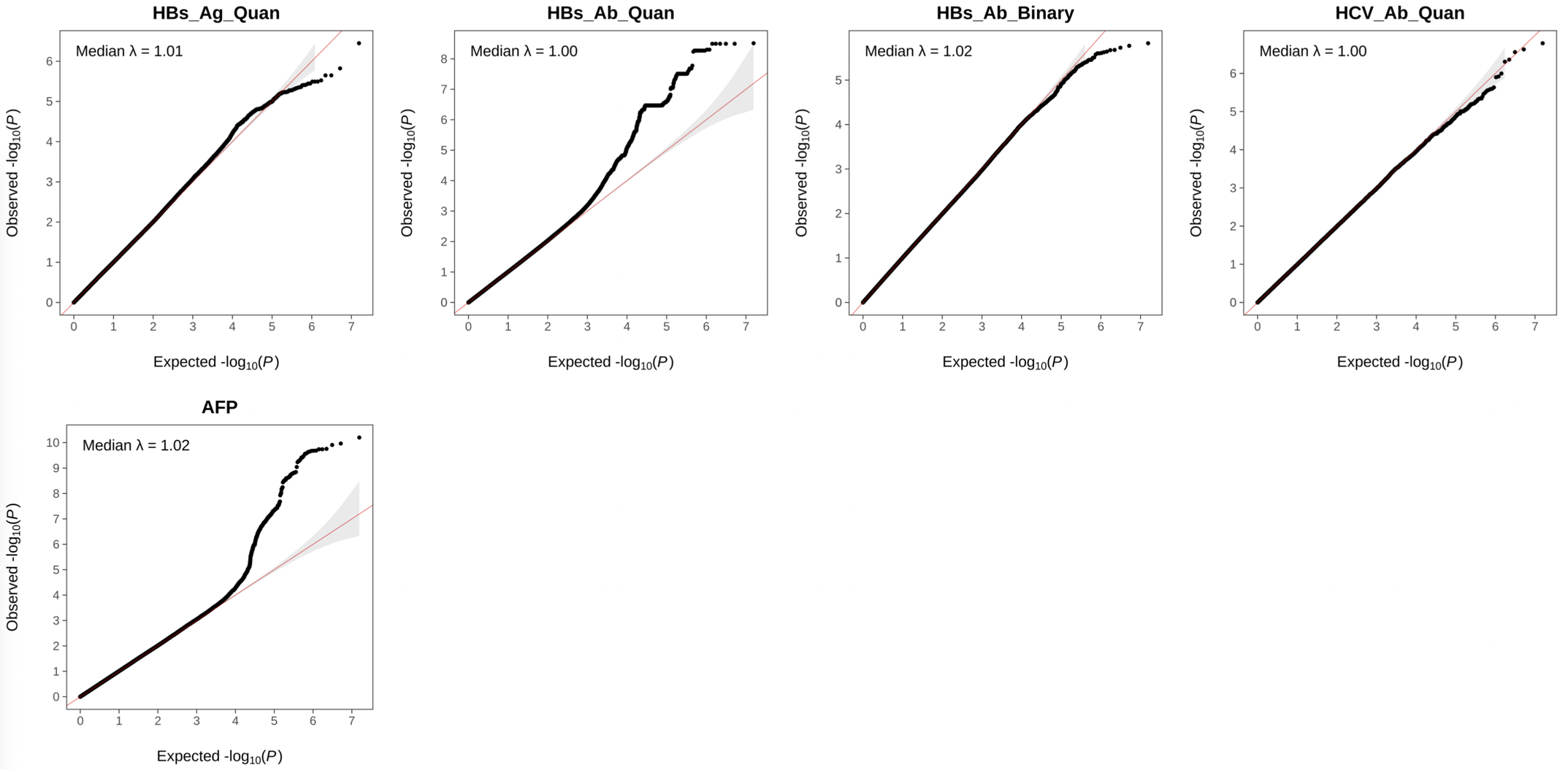


**Supplementary Fig. S12.** QQplots for the whole-genome-wide association tests of the traits on hepatitis category. X-axis indicates the expected -log_10_ *P*-value. Y-axis indicates observed -log_10_ *P*-value.


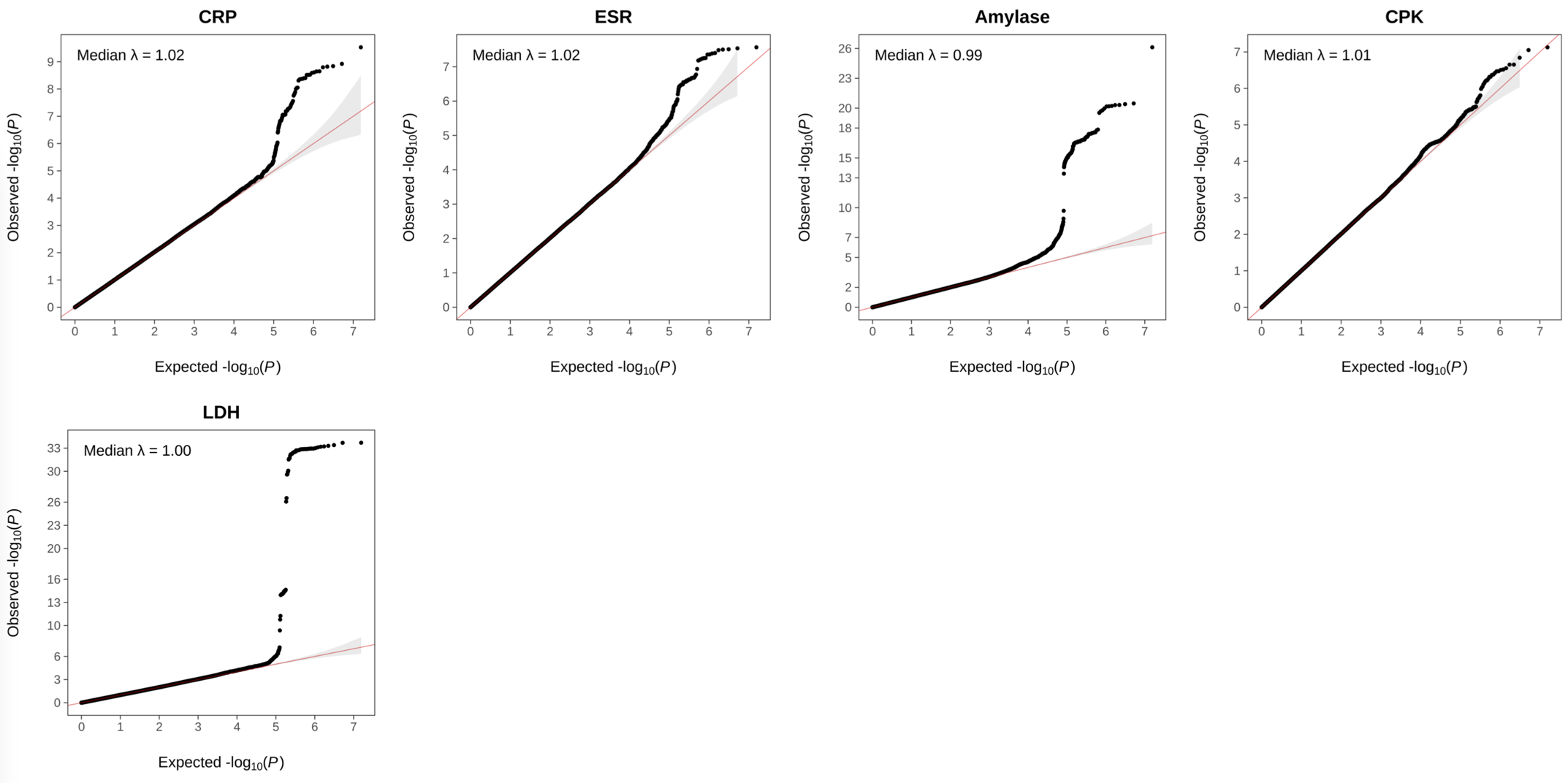


**Supplementary Fig. S13.** QQplots for the whole-genome-wide association tests of the traits on inflammation and etc category. X-axis indicates the expected -log_10_ *P*-value. Y-axis indicates observed -log_10_ *P*-value.


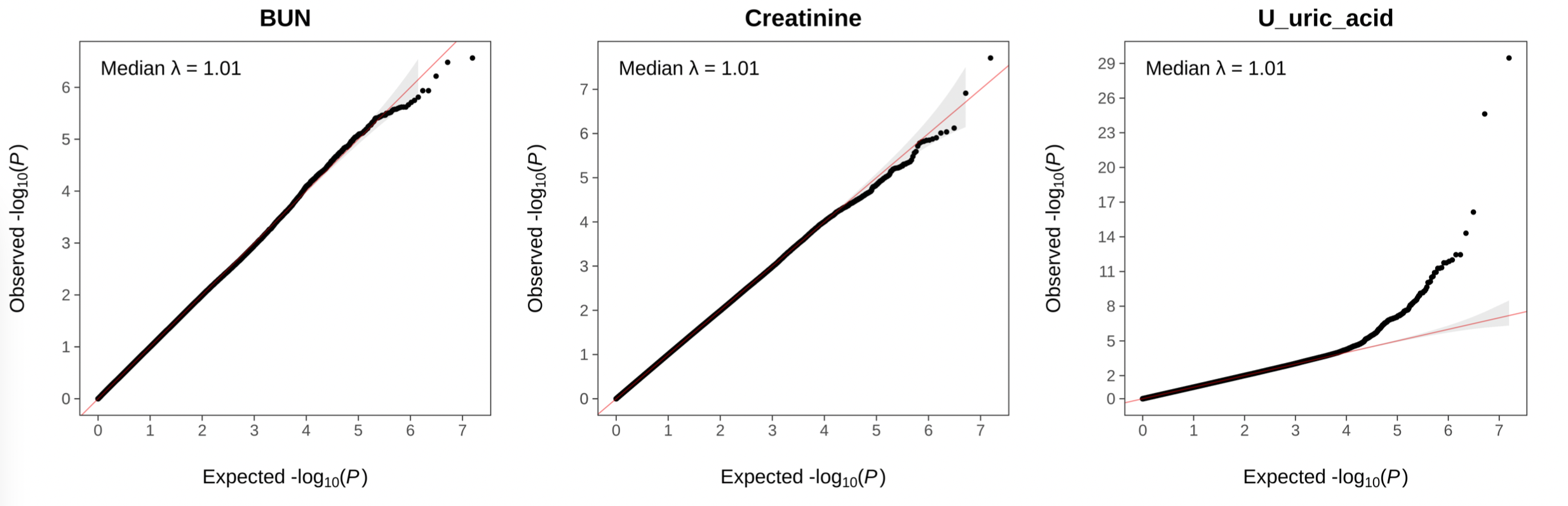


**Supplementary Fig. S14.** QQplots for the whole-genome-wide association tests of the traits on kidney function category. X-axis indicates the expected -log_10_ *P*-value. Y-axis indicates observed -log_10_ *P*-value.


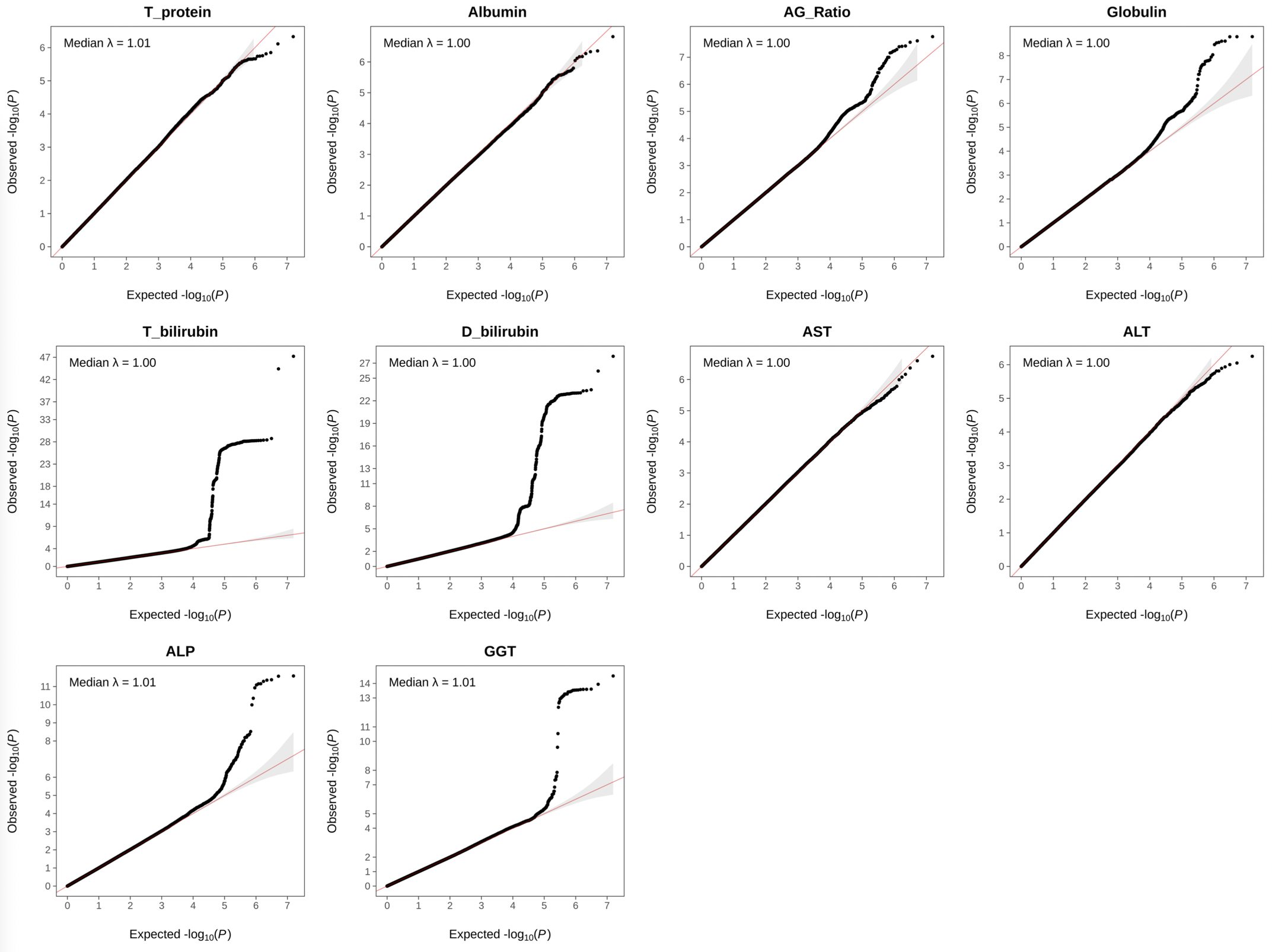


**Supplementary Fig. S15.** QQplots for the whole-genome-wide association tests of the traits on liver function category. X-axis indicates the expected -log_10_ *P*-value. Y-axis indicates observed -log_10_ *P*-value.


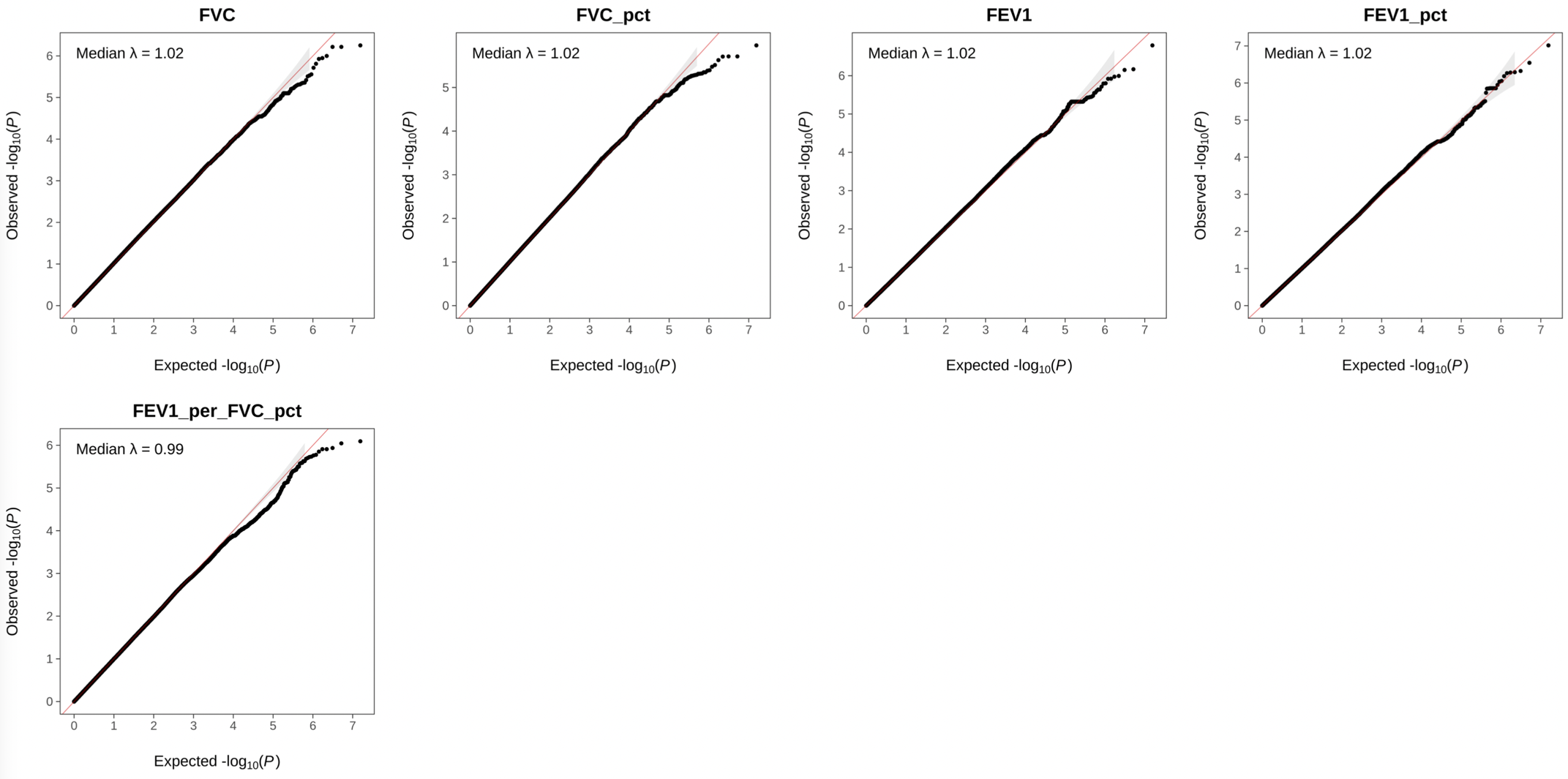


**Supplementary Fig. S16.** QQplots for the whole-genome-wide association tests of the traits on pulmonary function category. X-axis indicates the expected -log_10_ *P*-value. Y-axis indicates observed -log_10_ *P*-value.


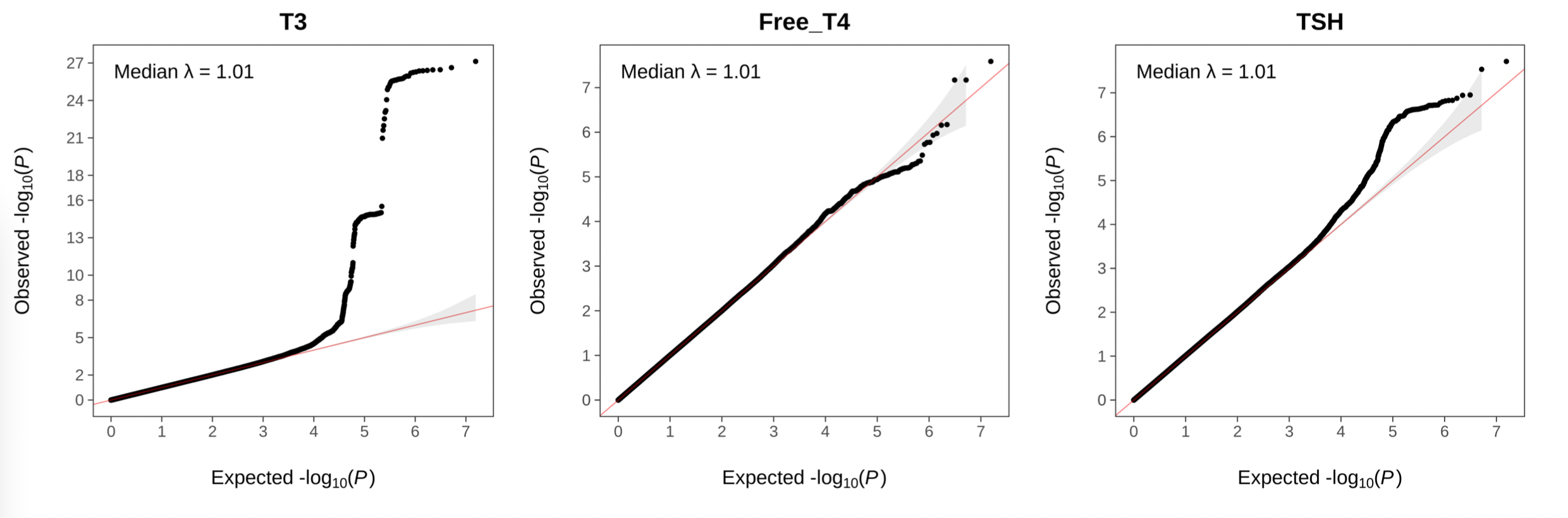


**Supplementary Fig. S17.** QQplots for the whole-genome-wide association tests of the traits on thyroid function category. X-axis indicates expected -log_10_ *P*-value. Y-axis indicates observed -log_10_ *P*-value.


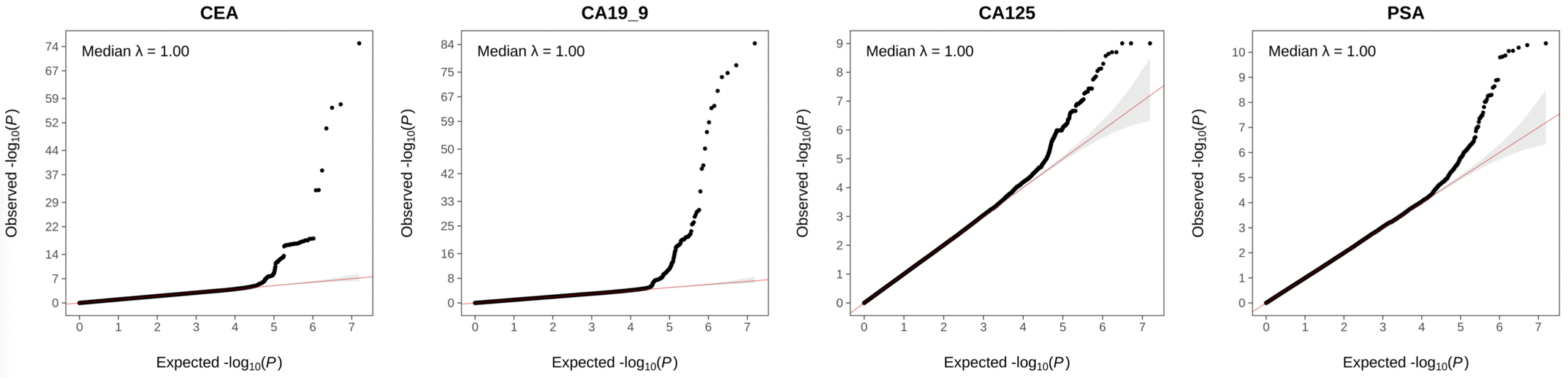


**Supplementary Fig. S18.** QQplots for the whole-genome-wide association tests of the traits on tumor biomarker category. X-axis indicates the expected -log_10_ *P*-value. Y-axis indicates observed -log_10_ *P*-value.


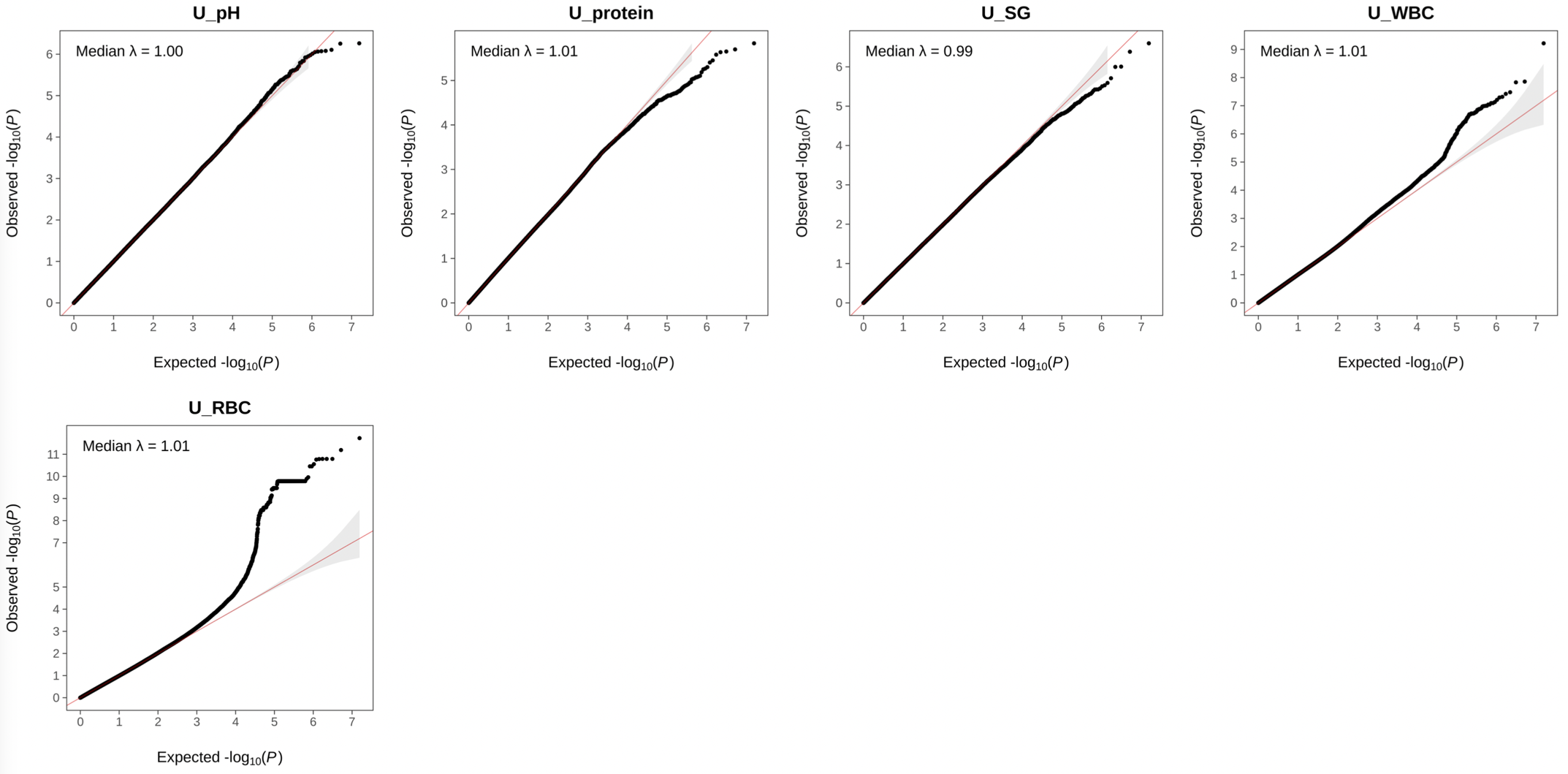


**Supplementary Fig. S19.** QQplots for the whole-genome-wide association tests of the traits on the urinalysis category. X-axis indicates the expected -log_10_ *P*-value. Y-axis indicates observed -log_10_ *P*-value.


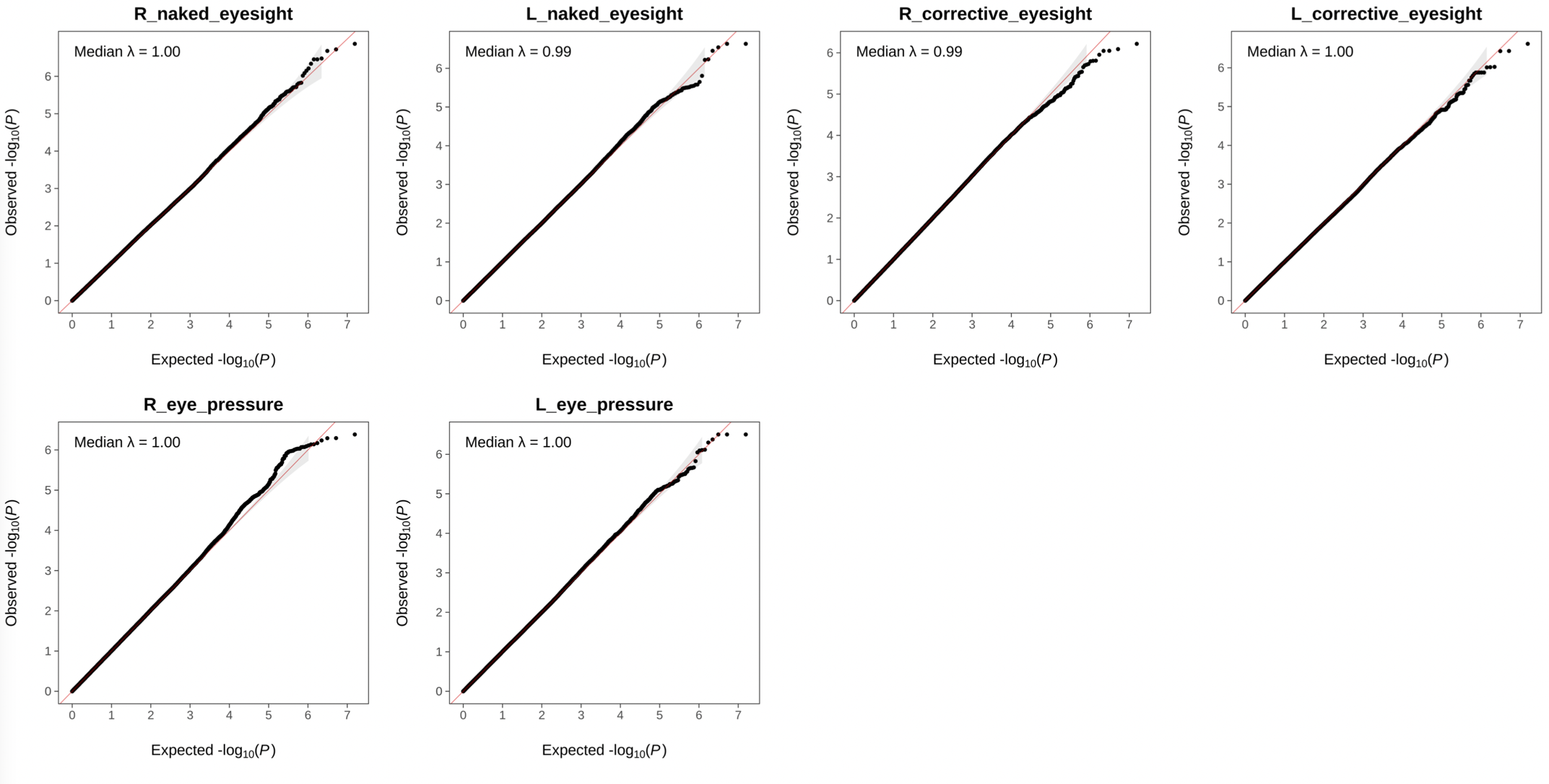


**Supplementary Fig. S20.** QQplots for the whole-genome-wide association tests of the traits on vision category. X-axis indicates the expected -log_10_ *P*-value. Y-axis indicates observed -log_10_ *P*-value.
