# Supplementary material for: Korea4K: whole genome sequences of 4,157 Koreans with 107 phenotypes derived from extensive health check-ups
Source: Gigascience. 2024 Apr 16;13:giae014. doi: 10.1093/gigascience/giae014 (PMC11020240; doi:10.1093/gigascience/giae014)
Supplement: giae014_Response_to_Reviewer_Comments_Original_Submission [file giae014_response_to_reviewer_comments_original_submission.pdf]

Author's Response To Reviewer Comments

GIGA-D-23-00109
Korea4K: whole genome sequences of 4,157 Koreans with 107 phenotypes derived from extensive health check-ups
Sungwon Jeon; Hansol Choi; Yeonsu Jeon; Whan-Hyuk Choi; Hyunjoo Choi; Kyungwhan An; Hyojung Ryu; Jihun Bhak; Hyeonjae Lee; Yoonsung Kwon; Sukyeon Ha; Yeo Jin Kim; Asta Blazyte; Changjae Kim; Yeonkyung Kim; Younghui Kang; Yeong Ju Woo; Chanyoung Lee; Jeongwoo Seo; Changhan Yoon; Dan Bolser; Orsolya Biro; Eun-Seok Shin; Byung Chul Kim; Seon-Young Kim; Ji-Hwan Park; Jongbum Jeon; Dooyoung Jung; Semin Lee; Jong Hwa Bhak
GigaScience

Reviewer reports:

Reviewer #1: This manuscript describes the second phase of the Korean Genome Project (KGP) with 4,157 sets of whole-genome data (designated Korea4K). After error correction and sequencing data curation, the whole-genome sequencing (WGS) data from 3,614 unrelated were used in the analyses. They also analyzed 107 types of clinical traits from 2,685 healthy participants' health check-up reports over a 4-year period (2016-2019). They performed a range of analyses and claimed that this new data performed better than Korea1K, the first phase KGP dataset, in a number of ways. A larger Korean dataset adds to the global genome resource and provides further insights into the Korean population. However, the results are mostly descriptive and serve as a catalog without significant new insights. The results are as expected (Korea4K is a better imputation reference panel than Korea1K, new variants are identified in the population, new variants are found in association with various phenotypes, etc.) and this dataset is sufficiently large to capture all the common variants found in the homogeneous Korean population.
The authors should address several issues:

1. The use of whole genome sequencing data in GWAS. The Bonferroni correction the authors used in their analysis was that for SNP array studies. They must do a formal correction with the many more variants found in WGS data and use a statistically sound correction for their analysis. The severe penalty for multiple testing using WGS data for GWAS is why few such studies have been done. I suspect that many of the associations will not reach statistical significance after proper correction, as the dataset is quite small for most traits under study.

⇒ Thank you for your critical comments regarding the statistics on the GWAS results. As you pointed out, we agree that there should be a stricter correction. As one method, we have now employed the FDR correction (Benjamini-Hochberg) which can remove possible false positives. The FDR values for each variant are now included in Supplementary Table S6 (List of the GWAS variants which have association significance P < 5E-8). After the FDR correction, 2314 variants from 30 traits still maintained statistically significant associations (FDR <0.05). We additionally noted the number of remaining variants and traits according to different FDR cutoffs in Table R1 below. Also, the results of the FDR correction were updated in the manuscript.

Page 11: "Among the significantly associated variants, 2,314 variants from 30 clinical traits still showed significance after false discovery rate (FDR) correction using the Benjamini-Hochberg approach (FDR < 0.05)."

Table R1. Number of significantly associated variants and traits according to FDR cutoffs

FDR cutoff	Number of variants	Number of traits
FDR < 0.10	2320	31
FDR < 0.05	2314	30
FDR < 0.01	2256	24
FDR < 0.005	2193	24

FDR < 0.001 1916 18

2. The authors should use the new genome references for their variant calling (T2T reference and the Human Pangenome Reference), as the GRCh38 is no longer the gold standard, and the results will be quite different with the most up-to-date references. Using the best human genome reference will make Korea4K more valuable.

⇒ We agree that we could potentially find more genetic markers that are related to the traits in our GWAS analysis, for example, when we use the T2T reference or the Human Pangenome Reference. However, there are a few considerations that limit us from using these references:

The T2T reference lacks enough annotation data which is critical. Many major genomic databases, such as dbSNP, are based on GRCh38. Thus, even if we were to use the T2T reference, we would have limitations in interpreting or validating the variants/markers we could additionally discover.

The draft Human Pangenome reference genome contains genomic sequences of 47 genetically diverse individuals, which requires a totally different bioinformatics pipeline to analyze. The bioinformatics analysis using the Human Pangenome reference is not fully established currently, which means that the validation method of the genetic markers that could be discovered should also be investigated more. Also, the Human Pangenome reference was assembled based on long-read sequencing data such as PacBio and Oxford Nanopore Technologies (ONT). As the authors of the Human Pangenome reference paper mentioned, the 1-base level of the sequencing accuracy can be an issue, which makes it hard to know if additional discoveries using the Pangenome are true signals or artifacts.

Furthermore, mapping the whole-genome sequencing reads for 4K samples and jointly genotyping the variants technically requires more than a year of time to rebuild the dataset.

We understand the importance of more precise and complete genome references for the variant calling and we appreciate your suggestion. We will expand the variant call set using the T2T and Human pangenome references in our future studies. Unfortunately, as we mentioned above, due to several technical limitations, we were not able to apply the new genome references in our current study, although we revised the manuscript to add the importance of the usage of these references.

Page 22: “Moreover, utilizing recently introduced human genome references like the T2T reference [33] and Human Pangenome reference [34], which offer broader genomic coverage or have population-specific sequences compared to the existing GRCh38 reference, could help identify additional associations that might be overlooked. Nevertheless, these new references lack functional annotations and need to be connected to previous databases such as dbSNP and the GWAS catalog.”

3. The authors should clarify how many of the participants who contributed clinical data are unrelated.

⇒ As described in the Methods, we filtered out a total of 540 individuals including 428 samples that have relatedness to other samples from 4,157 samples. Among the final unrelated 3,617 samples, 2,262 samples had clinical data. We updated the manuscript to provide a clear description of the participants who contributed to the clinical data.

Page 28: “Out of the final unrelated 3,617 samples, 2,374 samples had clinical data available and were included in the phenomics analyses.”

Reviewer #2: The authors contribute 4,157 whole-genome sequences (Korea4K) coupled with 107 health check-up parameters as the largest genomic resource of the Korean Genome Project. It has likely

characterized most of the common and very common genetic variants with commonly measured phenotypes for Koreans. It also discusses its applicability not only for the Korean population but also for other East Asian populations, and possibly to other national genome projects as well.

This work makes a significant contribution of data that can be used in future genome-wide association studies in the context of the Korean population. The manuscript appears to cover a lot of ground: from methodological issues to the real-world applications of the dataset in healthcare. The authors adopt innovative methods like GREML, which have been reported to have higher accuracy compared to older methods.

The authors are transparent about the limitations of their study, such as sample size and lack of sufficient data for rare diseases. They also acknowledge that phenomics analyses were not powerful enough for novel discoveries, indicating areas for future research. However, given the increasing importance of genomic data in healthcare and personalized medicine, the paper appears to be highly relevant.

While the paper is well formulated, there are some issues that need to be addressed before is accepted for publication.
See below:

1. You referred to the UK Biobank data for some of your analyses. Were there any limitations or caveats in comparing your dataset to the UK Biobank? What about other national genomic projects that are out there? How transferable do you think the Korea4K dataset would be to studies focusing on other populations outside East Asia?

⇒ Yes, there can be many limitations/caveats. However, please take in consideration that we cannot report the limitations precisely since we have not fully utilized the raw genomic and extensive clinical data from the UK Biobank but only the GWAS summary data in the current study.

One clear limitation is the difference in allele frequencies of reported variants between two ethnic groups in comparison due to different population genomic structures. This has been demonstrated in our PCA results (please refer to Figure 2). Even if the two populations were to have the same sample size, disparities in the allele frequencies would lead to different summary statistics (i.e., beta- and p-values). Therefore, the downstream, namely the phenomic, analysis could suffer from different resultant statistics and a fair comparison between the two independent studies could be difficult for some trait pairs.

Another possible limitation is that the current GWAS summary data provided by the UK Biobank (and other national biobanks) is based on arrays and many of the variants are imputed genotypes. Here, we have utilized the whole-genomes. We found that the GWAS-significant variants in comparison are not often overlapping due to technical biases as reported in our prior study (Jeon YS et. al., 2023, Hum Genet.). A possible solution to this would be to perform the joint genotyping of the Korean whole-genomes in conjunction with the UK Biobank whole-genomes. However, the entire process is resource-intensive and time-consuming, such that only the institutes with sufficient computing power will be able to process the data. In addition, rare variants appearing in each ethnic group will be grossly undermined.

The content and amount of phenotypic and clinical information provided are another possible limitation. Our health check-up data have been collected from multiple sources/centers such that we had to process the heterogeneous physical and digital copies of the health records and standardize them. On the other hand, all the participants in the UK Biobank were sampled in a single-centered manner with a unified procedure. Hence, our phenotypic and clinical information is much more limited than the UK Biobank's, and the method of measurements may differ for a few specific categories although we did not deeply investigate.

Furthermore, Korea4K data is not as readily accessible as the UK Biobank data. Obtaining it requires navigating through IRB processes and administrative procedures, and the legislative framework in South Korea does not currently facilitate a straightforward download process. If improved in the future, we will be able to make them more accessible. As an initial step toward enhanced accessibility, we have deposited our dataset in the European Genome-Phenome Archive (EGA) under the study accession 'EGAS00001007580'

and are actively working towards providing the WGS data openly and freely.
Moreover, we have included the accession number in the manuscript to facilitate easy reference.

Page 34: "The raw sequencing data that can be distributed were uploaded to the European Genome-Phenome Archive under the study accession 'EGAS00001007580'."

2. Could you expand on any ethical considerations that were taken into account, especially in terms of data privacy and informed consent?

⇒ Yes. In our project, we have taken ethical considerations, particularly concerning data privacy and informed consent, very seriously. The data we generated and used in our study comes from blood or saliva donations, and we have obtained explicit, full consent forms from the participants before getting the samples. These consent forms ensure that the participants are aware of how their data will be used and that they have willingly agreed to share this data for research purposes and IRB. As a result, we can make the data of 3,839 individuals publicly available while respecting the privacy and consent of the participants.

We have expanded the ethical considerations in "Ethics, consents and permissions":

Page 34: "The data employed in our study originates from voluntary blood or saliva donations, and we have diligently secured explicit, comprehensive consent forms from all participants prior to sample collection. These consent forms explicitly outline the intended use of their data for research purposes and underscore the voluntary nature of their participation. Furthermore, our study adheres to the ethical guidelines and regulations stipulated by the IRB. As a result, we can make the data of 3,839 individuals publicly available while respecting the privacy and consent of the participants."

3. How was the data cleaned and preprocessed, and were there any missing data points? If so, how were these handled? What number of reads(before and after QC), and other quality metrics do the sequenced reads have? What was the average coverage across the genome? What was the read length?

⇒ We cleaned and preprocessed by trimming possible adapter contamination on the sequencing reads and made the reads have at least 50bp of read length using the Cutadapt program (ver. 1.9.1). Then we confirmed the read counts, quality, and amount of bases using the FASTQC program. The average sequencing depth was 27.75 × and initial read lengths were 151bp. However, it varied after the trimming. The number of reads, average coverage, average quality, and filtering percentage were visualized in Supplementary Figure S4 and we updated the preprocessing procedure in the method section of the manuscript.

Page 23: "All the sequencing data that we used in this study had 151bp as a read length. Average sequencing amount per sample was 20× (Supplementary Figure S4)."

Page 24: "Adapter contamination was trimmed using Cutadapt (RRID: SCR_011841, ver. 1.9.1) [35] with a forward adapter ('GATCGGAAGAGCACACGTCTGAACTCCAGTCAC') and reverse adapter ('GATCGGAAGAGCGTCGTGTAGGGAAAGAGTGT') and with a minimum read length of 50 bp after trimming (Supplementary Figure S4)."

Page 24: "A total of 3,156 samples had a mapping depth of ≥ 20× (Supplementary Figure S4)."

4. How did you ensure the quality of the genomic data collected from different sources such as Korea1K and public data archives?

⇒ To ensure the quality, we rigorously utilized a standardized pipeline for processing the genomic data and

performed batch effect removal.

We applied the same bioinformatics analysis and QC pipelines as in the Korea1K (Jeon S et. al., 2020, Sci. Adv.). For example, we ensured the same version of the programs, and the same parameters through all the WGS data when we genotyped the samples.

We collected the WGS data from multiple sources or sequenced the whole genomes at different time points, which could suffer from the batch effect. After jointly genotyping the WGS data, we tried to reduce the batch effect from the different sequencing batches. As we noted in the manuscript, we applied the allele balance-based variant filtering.

The paper mentions mitigating batch effects through allele balance and manual checks. Could you provide more details on the methodology behind these checks and their efficiency?

⇒ As for the manual checks, we filtered out the undesired samples based on the following categories:
(1) high missing genotype rate (>10%);
(2) outlying heterozygous variants ratio (3 s.d.);
(3) high relatedness;
(4) non-Korean genetic background from PCA;
(5) having a rare disease;
(6) samples no longer available.

As for the allele balance, we first measured the average allele balance of the genotyped alleles (the read count of the allele divided by the total read count on a locus). Then, we excluded 12,713,580 variants that had an average allele balance of the loci out of the range of ± 1 × standard deviation (SD) from a genome-wide average of allele balance to remove the sequencing batch effect. We confirmed that the batch effects were removed after the filtering and visualized it as PCA plots (Supplementary Figure S1).

We have now added the definition of the allele balance in our Methods:

Page 25: "To detect variants which were probably called because of a sequencing batch effect, we measured average allele balance of the genotyped alleles (the read count of the allele divided by the total read count on a locus)."

5. Could you provide more information about the control group? Was it matched for age, sex, or other variables?

⇒ In the Korea4K dataset, we do not have a specific control set. We conducted our GWAS analysis only for the quantitative clinical traits using a linear regression approach without separately categorizing the study participants into case and control groups. Age, Sex, and BMI were included as covariates in testing the significance of variants across the clinical variables of interest.

How was the sample size determined, and does it provide enough statistical power to support your conclusions?

The sample size employed for each trait was maximized by utilizing the available samples in the Korea4K. Regarding the statistical power of GWAS, we recognize the importance of ensuring an adequate sample size to obtain robust results. We calculated the statistical power and effect size based on a likelihood ratio test by the R package ("genpwr"). Out of the 90 WGWAS traits analyzed, a majority of traits (77 traits) exhibited enough statistical powers exceeding 80% under the assumption of an effect size of 0.5 and a minor allele frequency (MAF) of 0.01 (added to Supplementary Table S14). Given the sufficient statistical power for the majority of traits examined. Our study's sample size of 4,157 individuals was appropriate for addressing the research objectives.

The detailed method is added to the manuscript:

Page 29: “Statistical powers of the 90 WGWAS were calculated by the R package “genpwr” under the assumption of an effect size of 0.5 and a minor allele frequency of 0.01 (Supplementary Table S14).”

6. You mentioned that the statistical power of your study will increase with more participants. Would this have implications for other national genomes that are making similar projects?

⇒ Probably yes and no. Yes, for other populations which are as homogenous as Koreans. The current study is very specific in that the population is very genetically homogenous. Koreans are probably the most homogenous population in East Asia. The diversity is even less than the Japanese archipelago (and the mainland China) because the Korean peninsula has been geographically isolated compared to other islandic populations. No, if a population is extremely heterogeneous with mixed ethnicities, the statistical power of our analysis would be much lower with the same sample size. In general, the statistical power will increase as the sample size goes up as you already know. However, we are unsure if the behavior would be exactly as our claim, and further studies are warranted.

Please elaborate on how your sensitivity analysis could apply to other populations outside Korea.

⇒ Thank you for a very interesting question. We find it very hard to answer because as mentioned above the Korean population is extremely homogeneous. It is a special population in terms of genetic and environmental diversity. Therefore, it is very difficult to estimate what kind of insight we may apply to other populations outside Korea. It could be an advantage or a disadvantage depending on the application of the population.

7. The paper acknowledges the sample size as not sufficiently large for detecting weak associations and admits that the sample size was not large enough to detect weak association signals. Have you considered statistical methods that can boost power in small samples?

⇒ We appreciate your comment. We could think of two statistical methods to improve statistical power: Meta-analysis and Gene-based association test. 1) Meta-analysis is one of the methods to boost statistical power for small samples by combining GWAS summary statistics of multiple independent studies. However, our study focuses specifically on the whole-genome-wide associations in the Korean population. Although a meta-analysis could improve the sensitivity of the weak association signals, the analysis might be able to introduce potential biases due to the genetic heterogeneity across populations. Thus, we have focused on our analysis of the Korean population. The Korean population’s genetic characteristics and their implications for clinical trait associations would provide an important aspect of our study, providing a unique and valuable. 2) Gene-based association test is another approach that increases the power to detect associations. This method aggregates the effects of multiple genetic variants within a gene to assess their collective impact. While the method is particularly advantageous for analyzing rare variants, we focused on identifying common variants associated with clinical traits in the Korean population. The application of gene-based association tests to our study could potentially yield additional insights, especially in the context of rare variant associations. We acknowledge the value of this approach and are considering its application in our subsequent study with a larger sample size.

8. Could you provide more details on the 107 clinical parameters used for the Korea4K phenome dataset? Were these parameters standardized across the different clinics and hospitals?

⇒ Yes, we standardized the parameters across the different clinics. For example, discrepancies in unit measurements, such as micrograms (ug) and nanograms (ng), were unified for specific traits, posing a direct challenge to the analytical process if these variations were not duly reconciled. Also, some parameters such as e-GFR were calculated by different equations across the clinics. We re-calculated such

parameters using a singular formula. More detailed methods were updated in the method section of the manuscript and Supplementary Table S13.

Page 28: "In the context of collecting data from over 200 diverse healthcare institutions, standardizing clinical information on 107 traits became imperative. We resolved discrepancies in unit measurements, such as micrograms and nanograms, for specific traits. Furthermore, certain clinical metrics, such as the estimated glomerular filtration rate (e-GFR), were found to exhibit variability contingent upon variables such as ethnicity, sex, and age. To maintain consistency and ensure methodological uniformity, we enforced the adoption of a singular clinical formula for the computation of e-GFR across all data samples. Such calculations were applied to 26 traits (Supplementary Table S13). Clinical traits that exhibited values characterized by inequalities likely due to the limit of detection (e.g., <5.0 and >99) were omitted from the analytical procedures, as such values have the potential to introduce disturbances to subsequent data analyses. Likewise, values that exhibited divergent formatting conventions across distinct healthcare institutions (e.g., 20 and a few or 999 and many) were harmonized to conform with prevailing standard criteria observed in most samples under investigation."

9. What criteria were used for initial sample filtering, particularly for excluding kinship? Could you clarify the steps taken to identify and filter the 64,301,272 SNVs and 8,776,608 Indels? How did you correct for batch effects arising from different Illumina NGS platforms and library preparations? Did you use specialized SNV calling software, or only GATK?

⇒ We have briefly mentioned this in our method section, we filtered out total 540 samples and the detailed filtering steps are noted in the method section as well. To exclude the samples who were in the kinship relationship, we first measured IBD values between the samples using Plink program (ver. 1.90b3n) and defined the family trees based on the IBD values which showed a PI_HAT value more than 0.05. Then, we filtered out samples in a family tree to have the maximum number of the remaining samples.

We also updated the manuscript to provide detailed procedures for defining the kinship relation between samples:

Page 25: "To explore kinship relations among the samples, we assessed Identical by Descent (IBD) using the Plink program (RRID:SCR_001757, ver. 1.90b3n) [30]. Samples with a PI_HAT value exceeding 0.05 were considered to be in a kinship relation."

About the variant calling, we did not use specialized SNV calling software. As we noted in the method section, we jointly genotyped the genotypes using only GATK 4.1.3 and identified the 64,301,272 SNVs and 8,776,608 Indels. With the jointly genotyped data, we measured allele balance of the loci. If the average allele balance of the loci was out of the range of $\pm 1 \times$ standard deviation (SD) from a genome-wide average of allele balance, the loci were treated as generated by possible batch effects due to different Illumina NGS platforms and library preparation and filtered out using an in-house script. A similar method was previously suggested by Muyas F, et. al., 2019. Following the filtering method, we excluded 12,713,580 variants and confirmed that the batch effects were removed through the PCA plots in Figure S1.

10. How were allele frequencies calculated and what considerations were made to interpret their biological significance? You mention that more than half of the singleton and doubleton variants were newly discovered. Could you elaborate on the methodology used to confirm these as novel variants?

⇒ The allele frequencies were calculated by the number of alternative alleles divided by the number of called alleles in a position. Generally, the allele frequency distribution can reflect the genomic diversity of the population. The definition we used to assign the variant as a novel variant is whether the variant was reported in the dbSNP database or not. We updated the figure legend of Figure 1 of the manuscript to provide the definition.

Page 9-10: “dbSNP indicates the variants were reported in dbSNP database. Novel indicates the variants were not reported in dbSNP.”

11. The section on phenotypic correlations mentions 2,274 trait-trait relationships. How would you address the potential for population stratification affecting the results of your genetic and phenotypic correlations?

⇒ The problem of population stratification in GWAS especially arises when conducting GWAS in multi-ethnic countries or meta-analyses across multiple sources of data with mixed ancestries. Here, our study exclusively deals with samples of Korean ancestry, which means our dataset is genetically homogeneous compared to other studies. To make sure, we excluded any samples with non-Korean genetic backgrounds based on PCA analysis as we noted in the “Sample and variant filtering” section in the Methods. Also, we included PC1~10 as covariates in our regression models during GWAS to avoid any latent ancestral effects from differential ethnic subgroups as we noted in the “Whole genome-wide association study (WGWAS)” section in the Methods.

We argue that such factors may add to reducing spurious correlations introduced by population stratification. Our claim is supported by values for the genomic inflation factor (λMedian) (Supplementary Figure S4-19). As you may already know, the value of lambda below 1.1 is generally considered acceptable indicating minimal false positives caused by gross population structure (and systematic biases) (please refer to Yang et. al., 2011, Eur J Hum Genet.)

We have now included the calculation of genomic inflation factor to estimate the gross population structure (and systematic biases) in our data.

Page 29: “Calculating the genomic inflation factor (λMedian), we found that all of the traits in the test reside below 1.1 indicating there are minimal false positives caused by gross population structure or systematic biases (Supplementary Figure S4-19) [48].”

How did you account for multiple comparisons in determining significant genetic correlations, and what corrections were applied to maintain the FDR?

⇒ We see that we were insufficient in our details as to how we corrected for multiple comparisons while calculating genetic correlation. We put our best effort to avoid false discoveries by conducting Benjamini-Hochberg correction to maintain the FDR well and below 0.05. Consistently, we applied the same correction method for setting FDR for phenotypic correlation as well.

We have now added the Methods for multiple testing correction for phenotypic correlation and genetic correlation:

Page 29: “Benjamini-Hochberg method was used to adjust for multiple comparisons when documenting confident phenotypic correlations with FDR.”

Page 30: “The correction for multiple tests was done by Benjamini-Hochberg approach when reporting confident GCs that suffice the threshold of FDR below 0.05.”

What measures were taken to ensure that the traits considered in this section were not subject to confounding and/or collider biases.

⇒ Thanks for pointing the important question. Confounding and collider biases are unavoidable when looking at multiple associations across numerous variables at once.

First, we employed covariate adjustment to reduce confounding biases by traits that are highly correlated with other clinical variables. We focused on Age, Sex, and BMI which have previously been suggested by Shungin et al. (2015) (Shungin D et. al., 2015, Nature). Second, we incorporated Mendelian Randomization (MR). MR is a statistical method to ascertain the direction of effect and imply possible causality devoid of confounders and colliders (Mitchell RE et. al., 2023, PLOS Genetics, and Ebrahim S and Davey Smith G, 2008, Human Genetics). To raise the confidence of our claim, we utilized three independent MR methods, namely IVW, MR-Egger, and MR-PRESSO, and documented the causal relationships if at least two of three were shown significant as noted in the Methods. However, we acknowledge that our methods of MR might still be prone to a collider bias, especially due to conditioning by covariates, which was to remove confounders and increase the power (Cai S et. al., 2022, Genetic Epidemiology). However, we would like to emphasize that such bias has minimal effect on the interpretation of phenotypic associations as previously reported by Pulit et al. (2019) (Pulit SL et. al., 2019, Hum Mol Genet).

We have added our responses to your comment into the Methods and Results, respectively:

Page 29: "Age and BMI were chosen especially due to their known shared associations with multiple traits as previously documented by Shungin and colleagues which could lead to confounding biases in the downstream interpretation of phenotypic relationships [47]."

Page 16: "In addition to the investigation on the general pleiotropic relationship, we employed Mendelian Randomization (MR) to detect vertical pleiotropy that can assert the direction of the phenotypic relationships [18]. This provides indirect evidence implying causality between the traits to discern spurious phenotypic associations, such as confounding and collider bias [19, 20]."

12. In your findings, Waist-Creatine showed opposite directions for genetic and phenotypic correlations. Could you elaborate on the potential implications or causes of this discrepancy?

⇒ Thanks for raising this point so that we could put attention here for a richer discussion. We suggest the discrepancy mainly comes from the shared environmental factors between Waist and Creatinine. This is well-reviewed by Sodini et al. (2018) (Sodini SM, et. al., 2018, Genetics). Most easy-to-understand case would be that from the dietary habits of individuals. It is well-known that the "meaty diet" readily elevates the serum creatinine level as well as the waist circumference (Khodayari S et. al., 2022, BMC Research Notes, and Pimenta E et. al., 2016, J Clin Epidemiol.). Hence, the higher the meat consumption, the higher the creatinine and wider the waist will be - a positive phenotypic correlation induced by the confounding effect of meat ingestion. We argue that the environmental effect would be exaggerated considering the relatively low heritability estimate of Creatinine.

We have now added the implication in our Results section:

Page 14: "Such discrepancies between the correlation estimates are possibly derived from the shared environmental factors between a pair of traits, such as dietary habits, that overwhelm the genotypic effects [12, 13]. This proves that the phenotypic correlation is not a mere proxy for the genetic correlation and consideration on the environmental effect is indispensable for the accurate interpretation of human phenomics [14]."

13. Were there any other surprising or unexpected correlations, and what are their potential implications?

⇒ Yes, there were a few surprising correlations, and we would like to emphasize the following:

1) Utility of Secondary Body Measures: WHtR, WWtR, BMI
WHtR (Waist-to-height Ratio) and WWtR (Waist-to-weight Ratio) are secondary body measures that come from combining two bodily measures, similar to Body Mass Index (BMI). Our phenomics results also depict distinguishable patterns of association between these secondary body measures with other phenotypes.

WHtR has a causal relationship with the C-reactive protein (CRP), Fat percentage, and HDL. On the other hand, WWtR showed associations with measures of lung capacity (FEV1 and FVC), liver function (AST), and inflammation (U_WBC). BMI positions as an intermediate phenotype, largely sharing its associations with WHtR and lightly with WWtR via left naked eyesight. These may reflect distinct biological mechanisms between the measurements warranting further studies. For example, WHtR is a well-known indicator of central adiposity, which serves as a better estimate obesity and related morbidities than BMI (Lee CMY et. al., 2008, J Clin Epidemiol.).

2) Complementary markers for cancer diagnosis: ALP and Amylase
CEA is a well-known biomarker for colon and lung cancer, while CA125 for ovarian. We found two independent causal relationships from these cancer biomarkers with other serum proteins that are seemingly irrelevant to cancerous phenotypes. Interestingly, our result suggests that alkaline phosphatase has influence on CEA. This implies that inflammation and dysfunction in the organs, such as liver or colon, precedes the alteration in the level of the cancer biomarker. It may be possible that ALP and CEA can both be used for detecting the presence of cancerous cells. Many early studies have reported their value in diagnosing cancer and monitoring metastasis in various types of cancer, including liver and colon, validating our finding (Aabo K et. al., 1986, Eur J Cancer Clin Oncol., Tartter PI et. al., 1981, Ann Surg., and Walach N et. al., 1989, J Surg Oncol.). Similarly, we could establish relationship between serum Amylase and CA125. Interestingly, we found cis-eQTLs underlying their pleiotropy are associated with the level of AMY2B expression in pancreas. Surprisingly, there have already been reports that patients with ovarian cancer manifest hyperamylasemia (Guo S et. al., 2018, Medicine, Shintani D, 2016, Eur J Gynaecol Oncol, and Zakrzewska I and Pietryńczak M, 1995). We argue that serum Amylase can be used as a complementary marker for ovarian cancer similar to ALP.

We elaborated our interesting correlations and their implications both in the Results and Discussion:

Page 18: "In our casual diagram (Figure 5, blue arrows), ALP and CEA showed potential causality, along with the shared genetic variants between them (pleiotropy near ABO gene). Numerous previous studies have consistently reported these markers together for diagnosing cancer and monitoring metastasis [21-23]. Similarly, CA125 and Amylase also displayed causality via shared genetic variants (pleiotropy near AMY2B gene). We propose that CA125 and Amylase might serve as complementary biomarkers for ovarian cancer, much like ALP and CEA. The biological relationships between these clinical blood measures remain unclear."

Page 18: "Our phenomics results also depicted distinguishable patterns of association between secondary body measures, such as WHtR (Waist-to-Height Ratio), WWtR (Waist-to-Weight Ratio), and BMI (Body Mass Index), with other phenotypes. WHtR exhibited a causal relationship with CRP (C-reactive protein), body fat percentage, and HDL. The result is concordant with previous reports that body fat percentage and CRP are correlated [24, 25]. Conversely, WWtR had casual associations with measures of lung capacity (FEV1 and FVC), liver function (AST), and inflammation (U_WBC). However, WWtR has yet to be proven its utility in clinical studies. BMI serves as an intermediate phenotype, sharing most of its associations with WHtR and, to a lesser extent, with WWtR via left naked eyesight. These findings suggest that the measurements reflect distinct biological mechanisms, warranting further studies. For instance, WHtR is a well-known indicator of central adiposity which provides a better estimate of obesity and related morbidities than BMI [26]."

Page 20: "Nevertheless, our findings bear important practical implications. We described the utility of secondary body measures, such as WHtR and WWtR, compared to BMI. We also elaborated on the diagnostic and prognostic value of other serum proteins, namely ALP and Amylase, in conjunction with the existing cancer biomarkers."

14. You mentioned that phenomics analyses were not powerful enough for novel discoveries. Could you elaborate more on what would be needed to make them more effective?
⇒ Yes, we can elaborate on a few points of improvement for a more effective study in the future. The current dataset of the Korea4K includes 4,157 healthy samples with no apparent disease onset at the time

of collection. Therefore, we could not see if our clinical variables, found from the phenomics analyses, have association on pathological conditions that is medically important. In future, we could use a wider variety of health-related categories to conduct a more powerful study, validating the current results with an enhanced scope which would bear invaluable medical and practical implications. Furthermore, collection of more samples for sequencing and health record data is also required for better chance of discovering new relationships.
We have added the following sentence in our Discussion:
Page 21: "However, we plan to collect more samples for sequencing and health record data with a wider variety of health-related categories to conduct a more powerful study in the future. This will allow us to not only validate our findings but also find correlations of medical importance that were missed in the present study."

15. For the future implications, in terms of healthcare and personalized medicine, what do you see as the most immediate applications of the Korea4K dataset?

⇒ Thank you for asking these important points. As we mentioned in "Potential Implications", the Korea4K dataset contains both whole-genome scale genotypes and matched clinical information. Thus, the dataset can immediately be applied to discover novel genetic markers that are associated with several phenotypes, diseases, or drug responses for Korean and East Asians. As a reference panel, the expanded genotype dataset (1K to 4K) can support more accurate genotyping imputation which is essential for DNA chip-based genotyping that is still widely used for healthcare (such as genetic tests). Furthermore, the Korea4K dataset can be used as a control data set across many different studies if the proper control samples are not applicable.
