# Supplementary material for: Korea4K: whole genome sequences of 4,157 Koreans with 107 phenotypes derived from extensive health check-ups
Source: Gigascience. 2024 Apr 16;13:giae014. doi: 10.1093/gigascience/giae014 (PMC11020240; doi:10.1093/gigascience/giae014)
Supplement: giae014_Reviewer_1_Report_Original_Submission — Pui-Yan Kwok -- 6/15/2023 Reviewed [file giae014_reviewer_1_report_original_submission.pdf]

Reviewer Report

Title: Korea4K: whole genome sequences of 4,157 Koreans with 107 phenotypes derived from extensive health check-ups

Version: Original Submission **Date:** 6/15/2023

Reviewer name: Pui-Yan Kwok

Reviewer Comments to Author:

This manuscript describes the second phase of the Korean Genome Project (KGP) with 4,157 sets of whole-genome data (designated Korea4K). After error correction and sequencing data curation, the whole-genome sequencing (WGS) data from 3,614 unrelated were used in the analyses. They also analyzed 107 types of clinical traits from 2,685 healthy participants' health check-up reports over a 4-year period (2016-2019). They performed a range of analyses and claimed that this new data performed better than Korea1K, the first phase KGP dataset, in a number of ways. A larger Korean dataset adds to the global genome resource and provides further insights into the Korean population. However, the results are mostly descriptive and serve as a catalog without significant new insights. The results are as expected (Korea4K is a better imputation reference panel than Korea1K, new variants are identified in the population, new variants are found in association with various phenotypes, etc.) and this dataset is sufficiently large to capture all the common variants found in the homogeneous Korean population.
The authors should address several issues:
1. The use of whole genome sequencing data in GWAS. The Bonferroni correction the authors used in their analysis was that for SNP array studies. They must do a formal correction with the many more variants found in WGS data and use a statistically sound correction for their analysis. The severe penalty for multiple testing using WGS data for GWAS is why few such studies have been done. I suspect that many of the associations will not reach statistical significance after proper correction, as the dataset is quite small for most traits under study.
2. The authors should use the new genome references for their variant calling (T2T reference and the Human Pangenome Reference), as the GRCh38 is no longer the gold standard and the results will be quite different with the most up-to-date references. Using the best human genome reference will make Korea4K more valuable.
3. The authors should clarify how many of the participants who contributed clinical data are unrelated.

Methods

Are the methods appropriate to the aims of the study, are they well described, and are necessary controls included? Choose an item.

Conclusions

Are the conclusions adequately supported by the data shown? Choose an item.

Reporting Standards

Does the manuscript adhere to the journal's guidelines on [minimum standards of reporting?]() Choose an item.

Choose an item.

Statistics

Are you able to assess all statistics in the manuscript, including the appropriateness of statistical tests used? Choose an item.

Quality of Written English

Please indicate the quality of language in the manuscript: Choose an item.

Declaration of Competing Interests

Please complete a declaration of competing interests, considering the following questions:

- Have you in the past five years received reimbursements, fees, funding, or salary from an organisation that may in any way gain or lose financially from the publication of this manuscript, either now or in the future?
- Do you hold any stocks or shares in an organisation that may in any way gain or lose financially from the publication of this manuscript, either now or in the future?
- Do you hold or are you currently applying for any patents relating to the content of the manuscript?
- Have you received reimbursements, fees, funding, or salary from an organization that holds or has applied for patents relating to the content of the manuscript?
- Do you have any other financial competing interests?
- Do you have any non-financial competing interests in relation to this paper?

If you can answer no to all of the above, write 'I declare that I have no competing interests' below. If your reply is yes to any, please give details below.

I declare that I have no competing interests.

I agree to the open peer review policy of the journal. I understand that my name will be included on my report to the authors and, if the manuscript is accepted for publication, my named report including any attachments I upload will be posted on the website along with the authors' responses. I agree for my report to be made available under an Open Access Creative Commons CC-BY license (http://creativecommons.org/licenses/by/4.0/). I understand that any comments which I do not wish to be included in my named report can be included as confidential comments to the editors, which will not be published.

Choose an item.

To further support our reviewers, we have joined with Publons, where you can gain additional credit to further highlight your hard work (see: https://publons.com/journal/530/gigascience). On publication of this paper, your review will be automatically added to Publons, you can then choose whether or not to claim your Publons credit. I understand this statement.

Yes Choose an item.
