# Supplementary material for: Korea4K: whole genome sequences of 4,157 Koreans with 107 phenotypes derived from extensive health check-ups
Source: Gigascience. 2024 Apr 16;13:giae014. doi: 10.1093/gigascience/giae014 (PMC11020240; doi:10.1093/gigascience/giae014)
Supplement: giae014_Reviewer_2_Report_Original_Submission — Taras K Oleksyk, Ph.D. -- 8/31/2023 Reviewed [file giae014_reviewer_2_report_original_submission.pdf]

Reviewer Report

Title: Korea4K: whole genome sequences of 4,157 Koreans with 107 phenotypes derived from extensive health check-ups

Version: Original Submission **Date:** 8/31/2023

Reviewer name: Taras K Oleksyk, Ph.D.

Reviewer Comments to Author:

The authors contribute 4,157 whole-genome sequences (Korea4K) coupled with 107 health check-up parameters as the largest genomic resource of the Korean Genome Project. It has likely characterized most of the common and very common genetic variants with commonly measured phenotypes for Koreans. It also discusses its applicability not only for the Korean population but also for other East Asian populations, and possibly to other national genome projects as well.

This work makes a significant contribution of data that can be used in future genome-wide association studies in the context of the Korean population. The manuscript appears to cover a lot of ground: from methodological issues to the real-world applications of the dataset in healthcare. The authors adopt innovative methods like GREML, which have been reported to have higher accuracy compared to older methods.

The authors are transparent about the limitations of their study, such as sample size and lack of sufficient data for rare diseases. They also acknowledge that phenomics analyses were not powerful enough for novel discoveries, indicating areas for future research. However, given the increasing importance of genomic data in healthcare and personalized medicine, the paper appears to be highly relevant.

While the paper is well formulated, there are some issues that need to be addressed before is accepted for publication.

See below:

1. You referred to the UK Biobank data for some of your analyses. Were there any limitations or caveats in comparing your dataset to the UK Biobank? What about other national genomic projects that are out there? How transferable do you think the Korea4K dataset would be to studies focusing on other populations outside East Asia?
2. Could you expand on any ethical considerations that were taken into account, especially in terms of data privacy and informed consent?
3. How was the data cleaned and preprocessed, and were there any missing data points? If so, how were these handled? What number of reads(before and after QC), and other quality metrics do the sequenced reads have? What was the average coverage across the genome? What was the read length?
4. How did you ensure the quality of the genomic data collected from different sources such as Korea1K and public data archives? The paper mentions mitigating batch effects through allele balance and manual checks. Could you provide more details on the methodology behind these checks and their efficiency?
5. Could you provide more information about the control group? Was it matched for age, sex, or other

variables? How was the sample size determined, and does it provide enough statistical power to support your conclusions?
6. You mentioned that the statistical power of your study will increase with more participants. Would this have implications for other national genomes that are making similar projects? Please elaborate on how your sensitivity analysis could apply to other populations outside Korea.
7. The paper acknowledges the sample size as not sufficiently large for detecting weak associations, and admits that the sample size was not large enough to detect weak association signals. Have you considered statistical methods that can boost power in small samples?
8. Could you provide more details on the 107 clinical parameters used for the Korea4K phenome dataset? Were these parameters standardized across the different clinics and hospitals?
9. What criteria were used for initial sample filtering, particularly for excluding kinship? Could you clarify the steps taken to identify and filter the 64,301,272 SNVs and 8,776,608 Indels? How did you correct for batch effects arising from different Illumina NGS platforms and library preparations? Did you use specialized SNV calling software, or only GATK?
10. How were allele frequencies calculated and what considerations were made to interpret their biological significance? You mention that more than half of the singleton and doubleton variants were newly discovered. Could you elaborate on the methodology used to confirm these as novel variants?
11. The section on phenotypic correlations mentions 2,274 trait-trait relationships. How would you address the potential for population stratification affecting the results of your genetic and phenotypic correlations? How did you account for multiple comparisons in determining significant genetic correlations, and what corrections were applied to maintain the FDR? What measures were taken to ensure that the traits considered in this section were not subject to confounding and/or collider biases.
12. In your findings, Waist-Creatine showed opposite directions for genetic and phenotypic correlations. Could you elaborate on the potential implications or causes of this discrepancy?
13. Were there any other surprising or unexpected correlations, and what are their potential implications?
14. You mentioned that phenomics analyses were not powerful enough for novel discoveries. Could you elaborate more on what would be needed to make them more effective?
15. For the future implications, in terms of healthcare and personalized medicine, what do you see as the most immediate applications of the Korea4K dataset?

Methods

Are the methods appropriate to the aims of the study, are they well described, and are necessary controls included? Choose an item.

Conclusions

Are the conclusions adequately supported by the data shown? Choose an item.

Reporting Standards

Does the manuscript adhere to the journal's guidelines on minimum standards of reporting? Choose an item.

Choose an item.

Statistics

Are you able to assess all statistics in the manuscript, including the appropriateness of statistical tests used? Choose an item.

Quality of Written English

Please indicate the quality of language in the manuscript: Choose an item.

Declaration of Competing Interests

Please complete a declaration of competing interests, considering the following questions:

- Have you in the past five years received reimbursements, fees, funding, or salary from an organisation that may in any way gain or lose financially from the publication of this manuscript, either now or in the future?
- Do you hold any stocks or shares in an organisation that may in any way gain or lose financially from the publication of this manuscript, either now or in the future?
- Do you hold or are you currently applying for any patents relating to the content of the manuscript?
- Have you received reimbursements, fees, funding, or salary from an organization that holds or has applied for patents relating to the content of the manuscript?
- Do you have any other financial competing interests?
- Do you have any non-financial competing interests in relation to this paper?

If you can answer no to all of the above, write 'I declare that I have no competing interests' below. If your reply is yes to any, please give details below.

I declare that I have no competing interests

I agree to the open peer review policy of the journal. I understand that my name will be included on my report to the authors and, if the manuscript is accepted for publication, my named report including any attachments I upload will be posted on the website along with the authors' responses. I agree for my report to be made available under an Open Access Creative Commons CC-BY license (http://creativecommons.org/licenses/by/4.0/). I understand that any comments which I do not wish to be included in my named report can be included as confidential comments to the editors, which will not be published.

Choose an item.

To further support our reviewers, we have joined with Publons, where you can gain additional credit to further highlight your hard work (see: https://publons.com/journal/530/gigascience). On publication of this paper, your review will be automatically added to Publons, you can then choose whether or not to claim your Publons credit. I understand this statement.

Yes Choose an item.
